# Supplementary material for: Considerations in the reliability and fairness audits of predictive models for advance care planning
Source: Front Digit Health. 2022 Sep 12;4:943768. doi: 10.3389/fdgth.2022.943768 (PMC9634737; doi:10.3389/fdgth.2022.943768)
Supplement: Supplementary file 1 [file Datasheet1.pdf]

# Supplement - Considerations in the Reliability and Fairness Audits of Predictive Models for Advance Care Planning

Jonathan Lu[1], Amelia Sattler[2†], Samantha Wang[3†], Ali Raza Khaki[4†], Alison Callahan[1], Scott Fleming[1], Rebecca Fong[5], Benjamin Ehlert[1], Ron C. Li[3], Lisa Shieh[3], Kavitha Ramchandran[4], Michael F. Gensheimer[6], Sarah Chobot[7], Stephen Pfohl[1], Siyun Li[1], Kenny Shum[8], Nitin Parikh[8], Priya Desai[8], Briththa Seevaratnam[5], Melanie Hanson[5], Margaret Smith[9], Yizhe Xu[1], Arjun Gokhale[1], Steven Lin[9], Michael A. Pfeffer[3,8], Winifred Teuteberg[5††], Nigam H. Shah[1,8,10††]

[†] These authors have contributed equally to this work.
[††] These authors have contributed equally to this work and share senior authorship.

[1] Center for Biomedical Informatics Research, Department of Medicine, Stanford University School of Medicine
[2] Division of Primary Care and Population Health, Department of Medicine, Stanford University School of Medicine
[3] Division of Hospital Medicine, Department of Medicine, Stanford University School of Medicine
[4] Division of Oncology, Department of Medicine, Stanford University School of Medicine
[5] Serious Illness Care Program, Department of Medicine, Stanford University School of Medicine
[6] Department of Radiation Oncology, Stanford University School of Medicine
[7] Stanford Health Care
[8] Technology & Digital Solutions, Stanford Health Care and Stanford University School of Medicine
[9] Stanford Healthcare AI Applied Research Team, Stanford University School of Medicine
[10] Clinical Excellence Research Center, Stanford University School of Medicine

**Supplemental Methods** 5
Survey Instrument 5

**Supplemental Files:** 17
Supplemental File 1: Time and Requirements to Generate Reliability/Fairness Audit. 17

**Supplemental Figures:** 17
Supplemental Figure 1: Message to Primary Care clinicians soliciting surprise questions, staff message. 17
Supplemental Figure 2: Message to Hospital Medicine physicians soliciting surprise questions, email. 18

**Supplemental Tables:** 19
Main Analysis 19
Epic EOL Low Threshold in Primary Care 19
Supplemental Table 1: Epic EOL Low Threshold in Primary Care: Reliability and Fairness Audit by Sex. Significant differences in prevalence, significantly lower performance, or significantly higher O/E are bolded. 19
Supplemental Table 2: Epic EOL Low Threshold in Primary Care: Reliability and Fairness Audit by Age. Significant differences in prevalence, significantly lower performance, or significantly higher O/E are bolded. 20

Supplemental Table 3: Epic EOL Low Threshold in Primary Care: Reliability and Fairness Audit by Ethnicity/Race. Significant differences in prevalence, significantly lower performance, or significantly higher O/E are bolded. 22

Supplemental Table 4: Epic EOL Low Threshold in Primary Care: Reliability and Fairness Audit by Ethnicity/Race and Sex. Significant differences in prevalence, significantly lower performance, or significantly higher O/E are bolded. 24

Epic EOL High Threshold in Inpatient Oncology 24

Supplemental Table 5: Epic EOL High Threshold in Inpatient Oncology: Reliability and Fairness Audit by Sex. Prevalence, performance and calibration is presented for the overall cohort and for subgroups with significant differences in prevalence, significantly lower performance, or significantly higher O/E (bolded). 25

Supplemental Table 6: Epic EOL High Threshold in Inpatient Oncology: Reliability and Fairness Audit by Age. Significant differences in prevalence, significantly lower performance, or significantly higher O/E are bolded. 26

Supplemental Table 7: Epic EOL High Threshold in Inpatient Oncology: Reliability and Fairness Audit by Ethnicity/Race. Significant differences in prevalence, significantly lower performance, or significantly higher O/E are bolded. 27

Supplemental Table 8: Epic EOL High Threshold in Inpatient Oncology: Reliability and Fairness Audit by Ethnicity/Race and Sex. Significant differences in prevalence, significantly lower performance, or significantly higher O/E are bolded. 29

Stanford HM ACP in Inpatient Oncology 29

Supplemental Table 9: Stanford HM ACP in Inpatient Oncology: Reliability and Fairness Audit by Sex. Significant differences in prevalence, significantly lower performance, or significantly higher O/E are bolded. 29

Supplemental Table 10: Stanford HM ACP in Inpatient Oncology: Reliability and Fairness Audit by Age. Significant differences in prevalence, significantly lower performance, or significantly higher O/E are bolded. 30

Supplemental Table 11: Stanford HM ACP in Inpatient Oncology: Reliability and Fairness Audit by Ethnicity/Race. Significant differences in prevalence, significantly lower performance, or significantly higher O/E are bolded. 31

Supplemental Table 12: Stanford HM ACP in Inpatient Oncology: Reliability and Fairness Audit by Ethnicity/Race and Sex. Significant differences in prevalence, significantly lower performance, or significantly higher O/E are bolded. 33

Epic EOL High Threshold in Hospital Medicine 33

Supplemental Table 13: Epic EOL High Threshold in Hospital Medicine: Reliability and Fairness Audit by Sex. Significant differences in prevalence, significantly lower performance, or significantly higher O/E are bolded. 33

Supplemental Table 14: Epic EOL High Threshold in Hospital Medicine: Reliability and Fairness Audit by Age. Significant differences in prevalence, significantly lower performance, or significantly higher O/E are bolded. 34

Supplemental Table 15: Epic EOL High Threshold in Hospital Medicine: Reliability and Fairness Audit by Ethnicity/Race. Significant differences in prevalence, significantly lower performance, or significantly higher O/E are bolded. 35

Supplemental Table 16: Epic EOL High Threshold in Hospital Medicine: Reliability and Fairness Audit by Ethnicity/Race and Sex. Significant differences in prevalence, significantly lower performance, or significantly higher O/E are bolded. 38
Stanford HM ACP in Hospital Medicine 38
Supplemental Table 17: Stanford HM ACP in Hospital Medicine: Reliability and Fairness Audit by Sex. Significant differences in prevalence, significantly lower performance, or significantly higher O/E are bolded. 38
Supplemental Table 18: Stanford HM ACP in Hospital Medicine: Reliability and Fairness Audit by Age. Significant differences in prevalence, significantly lower performance, or significantly higher O/E are bolded. 39
Supplemental Table 19: Stanford HM ACP in Hospital Medicine: Reliability and Fairness Audit by Ethnicity/Race. Significant differences in prevalence, significantly lower performance, or significantly higher O/E are bolded. 40
Supplemental Table 20: Stanford HM ACP in Hospital Medicine: Reliability and Fairness Audit by Ethnicity/Race and Sex. Significant differences in prevalence, significantly lower performance, or significantly higher O/E are bolded. 42
Clinical Decision Maker Survey Responses 43
Supplemental Table 21: Survey responses to “What are the first thoughts that came to your mind on seeing the results of the reliability and fairness audit?” 45
Supplemental Table 22: Survey responses to “Is there any other information you would want included in this audit to support your decision on whether to deploy a model? If so, what?” 46
Supplemental Table 23: Survey responses to “What are some key drivers to making these reliability and fairness audits standard practice?” 46
Supplemental Table 24: Survey responses to “What are some key barriers to making these reliability and fairness audits standard practice?” 48
Supplemental Table 25: Survey responses to “As a clinical decisionmaker, what pros do you see in using an AI model to support your work?” 48
Supplemental Table 26: Survey responses to “As a clinical decisionmaker, what cons do you see in using an AI model to support your work?” 49
Class-Balanced Analysis 50
Epic EOL Low Threshold in Primary Care 50
Supplemental Table 27: Epic EOL Low Threshold in Primary Care: Class Balanced Reliability and Fairness Audit by Sex. Significant differences in prevalence, significantly lower performance, or significantly higher O/E are bolded. 50
Supplemental Table 28: Epic EOL Low Threshold in Primary Care: Class Balanced Reliability and Fairness Audit by Age. Significant differences in prevalence, significantly lower performance, or significantly higher O/E are bolded. 51
Supplemental Table 29: Epic EOL Low Threshold in Primary Care: Class Balanced Reliability and Fairness Audit by Ethnicity/Race. Significant differences in prevalence, significantly lower performance, or significantly higher O/E are bolded. 53

Supplemental Table 30: Epic EOL Low Threshold in Primary Care: Class Balanced Reliability and Fairness Audit by Ethnicity/Race and Sex. Significant differences in prevalence, significantly lower performance, or significantly higher O/E are bolded. 55

Epic EOL High Threshold in Inpatient Oncology 55

Supplemental Table 31: Epic EOL High Threshold in Inpatient Oncology: Class Balanced Reliability and Fairness Audit by Sex. Prevalence, performance and calibration is presented for the overall cohort and for subgroups with significant differences in prevalence, significantly lower performance, or significantly higher O/E (bolded). 55

Supplemental Table 32: Epic EOL High Threshold in Inpatient Oncology: Class Balanced Reliability and Fairness Audit by Age. Significant differences in prevalence, significantly lower performance, or significantly higher O/E are bolded. 56

Supplemental Table 33: Epic EOL High Threshold in Inpatient Oncology: Class Balanced Reliability and Fairness Audit by Ethnicity/Race. Significant differences in prevalence, significantly lower performance, or significantly higher O/E are bolded. 57

Supplemental Table 34: Epic EOL High Threshold in Inpatient Oncology: Class Balanced Reliability and Fairness Audit by Ethnicity/Race and Sex. Significant differences in prevalence, significantly lower performance, or significantly higher O/E are bolded. 59

Stanford HM ACP in Inpatient Oncology 59

Supplemental Table 35: Stanford HM ACP in Inpatient Oncology: Class Balanced Reliability and Fairness Audit by Sex. Significant differences in prevalence, significantly lower performance, or significantly higher O/E are bolded. 60

Supplemental Table 36: Stanford HM ACP in Inpatient Oncology: Class Balanced Reliability and Fairness Audit by Age. Significant differences in prevalence, significantly lower performance, or significantly higher O/E are bolded. 61

Supplemental Table 37: Stanford HM ACP in Inpatient Oncology: Class Balanced Reliability and Fairness Audit by Ethnicity/Race. Significant differences in prevalence, significantly lower performance, or significantly higher O/E are bolded. 62

Supplemental Table 38: Stanford HM ACP in Inpatient Oncology: Class Balanced Reliability and Fairness Audit by Ethnicity/Race and Sex. Significant differences in prevalence, significantly lower performance, or significantly higher O/E are bolded. 64

Epic EOL High Threshold in Hospital Medicine 64

Supplemental Table 39: Epic EOL High Threshold in Hospital Medicine: Class Balanced Reliability and Fairness Audit by Sex. Significant differences in prevalence, significantly lower performance, or significantly higher O/E are bolded. 65

Supplemental Table 40: Epic EOL High Threshold in Hospital Medicine: Class Balanced Reliability and Fairness Audit by Age. Significant differences in

prevalence, significantly lower performance, or significantly higher O/E are bolded. 66

Supplemental Table 41: Epic EOL High Threshold in Hospital Medicine: Class Balanced Reliability and Fairness Audit by Ethnicity/Race. Significant differences in prevalence, significantly lower performance, or significantly higher O/E are bolded. 67

Supplemental Table 42: Epic EOL High Threshold in Hospital Medicine: Class Balanced Reliability and Fairness Audit by Ethnicity/Race and Sex. Significant differences in prevalence, significantly lower performance, or significantly higher O/E are bolded. 68

Stanford HM ACP in Hospital Medicine 69

Supplemental Table 43: Stanford HM ACP in Hospital Medicine: Class Balanced Reliability and Fairness Audit by Sex. Significant differences in prevalence, significantly lower performance, or significantly higher O/E are bolded. 69

Supplemental Table 44: Stanford HM ACP in Hospital Medicine: Class Balanced Reliability and Fairness Audit by Age. Significant differences in prevalence, significantly lower performance, or significantly higher O/E are bolded. 70

Supplemental Table 45: Stanford HM ACP in Hospital Medicine: Class Balanced Reliability and Fairness Audit by Ethnicity/Race. Significant differences in prevalence, significantly lower performance, or significantly higher O/E are bolded. 71

Supplemental Table 46: Stanford HM ACP in Hospital Medicine: Class Balanced Reliability and Fairness Audit by Ethnicity/Race and Sex. Significant differences in prevalence, significantly lower performance, or significantly higher O/E are bolded. 73

# Supplemental Methods

## *Survey Instrument*

# Reliability and Fairness Audit - Clinical Decision maker Survey

jhlu@stanford.edu (not shared) Switch account

* Required

As a clinical decisionmaker, what pros do you see in using an AI model to support your work? *

Please check all that apply.

- [ ] Helps triage patients and identify who would benefit the most
- [ ] Reduces work for me
- [ ] Shared understanding of patients for our whole care team
- [ ] I do not see any pros to using an AI model to support my work.
- [ ] Other:

As a clinical decisionmaker, what cons do you see in using an AI model to support your work? *

Please check all that apply.

- [ ] I disagree with the model
- [ ] Loss of my decisionmaking autonomy
- [ ] Pressure to act even if I disagree with the model
- [ ] Lack of transparency of the model
- [ ] Takes effort to maintain
- [ ] I do not see any cons to using an AI model to support my work.
- [ ] Other:

What does it mean to you for a model to be reliable? *

Your answer

What does it mean to you for a model to be fair? *

Your answer

What are the first thoughts that came to your mind on seeing the results of the reliability and fairness audit? *

Your answer

For each component of the audit, would it affect your decision to deploy a model? *

| | Yes, this would affect my decision to deploy the model. | No, this would not affect my decision to deploy the model. | Prefer not to answer |
|---|---|---|---|
| Summary Statistics | ○ | ○ | ○ |
| Performance | ○ | ○ | ○ |
| Calibration | ○ | ○ | ○ |
| Subgroup Performance | ○ | ○ | ○ |
| Subgroup Calibration | ○ | ○ | ○ |

Is there any other information you would want included in this audit to support your decision on whether to deploy a model? If so, what?

Your answer

How many minutes on average did it take you to answer the surprise question for one patient? *

Your answer

What are some key drivers to making these reliability and fairness audits standard practice? *

- [ ] Findings that AI models are not reliable
- [ ] Findings that AI models are not fair
- [ ] Academic medicine's push toward racial equity
- [ ] I do not see any key drivers to making reliability and fairness audits standard practice.
- [ ] Other:

What are some key barriers to making these reliability and fairness audits standard practice? *

- [ ] Lack of data access
- [ ] Poor data quality
- [ ] Poor demographic data quality
- [ ] Lack of knowledge about how to do an audit
- [ ] Lack of data science expertise in my practice setting
- [ ] The reliability of deployed AI models is not prioritized
- [ ] The fairness of deployed AI models is not prioritized
- [ ] Audits are not built into our incentives
- [ ] I do not see any barriers to making reliability and fairness audits standard practice.
- [ ] Other:

Please describe your role as a clinical decisionmaker in your setting. *

Your answer

Any other questions or comments related to this audit?

Your answer

Name *

Your answer

Setting *

- Inpatient Oncology
- Primary Care
- Hospital Medicine
- Other:

Submit Clear form

Never submit passwords through Google Forms.

This form was created inside of Stanford University. Report Abuse

Google Forms

# Supplemental Results

## Reliability and Fairness Audit using AUROC and Accuracy metrics

We report the reliability and fairness audits using AUROC and Accuracy as metrics. For the fairness audit, we report metrics only for subgroups that were significantly different from the overall group. All values and confidence Intervals can be found in Supplemental Tables 1-25.

### *Primary Care*

#### Epic EOL Low Threshold in Primary Care (AUROC/Accuracy)

The Epic EOL Low Threshold model had an AUROC of 0.91 and accuracy of 0.86. Accuracy was lower for Age: (80, 90] at 0.61. There were no statistically significant differences in AUROC (Supplemental Tables 1-4).

### *Inpatient Oncology*

#### Epic EOL High Threshold in Inpatient Oncology (AUROC/Accuracy)

The Epic EOL High Threshold model had an AUROC of 0.70 and an accuracy of 0.46 (Supplemental Tables 5-8).

There were statistically significant differences for several subgroups that had less than 10 people in the data set, making results inconclusive. AUROC was lower for Ethnicity: Hispanic or Latino, Race: White (n = 3) at 0. Accuracy was lower for Ethnicity: Hispanic or Latino, Race: White, Sex: Female (n = 1) at 0 (Supplemental Tables 5-8).

#### Stanford HM ACP in Inpatient Oncology (AUROC/Accuracy)

The Stanford HM ACP model had an AUROC of 0.85 and an accuracy of 0.79. AUROC was lower for Age: (80, 90] at 0.3 (Supplemental Tables 9-12).

There were statistically significant differences for several subgroups that had less than 10 people in the data set, making results inconclusive. AUROC was lower for Ethnicity: Not Hispanic or Latino, Race: Black or African American (n = 3) at 0 and Ethnicity: Not Hispanic or Latino, Race: Black or African American, Sex: Female (n = 2) at 0. Accuracy was lower for Ethnicity: Hispanic or Latino, Race: White (n = 3) at 0.33, Ethnicity: Not Hispanic or Latino, Race: Black or African American (n = 3) at 0.33, Ethnicity: Not Hispanic or Latino, Race: American Indian or Alaska Native (n = 1) at 0, Ethnicity: Hispanic or Latino, Race: White, Sex: Male (n = 2) at 0, Ethnicity: Not Hispanic or Latino, Race: Black or African American, Sex: Female (n = 2) at 0, and Ethnicity: Not Hispanic or Latino, Race: American Indian or Alaska Native, Sex: Male (n = 1) at 0 (Supplemental Tables 9-12).

#### Model Comparison in Inpatient Oncology (AUROC/Accuracy)

The Stanford HM ACP model had a significantly higher accuracy (0.79) than the Epic EOL High Threshold (0.46). There was no statistically significant difference between the models in AUROC.

### *Hospital Medicine*

### Epic EOL High Threshold in Hospital Medicine (AUROC/Accuracy)

The Epic EOL High Threshold model had an AUROC of 0.78 and an accuracy of 0.62. AUROC was lower for Age: (30, 40] at 0.49 and Age: (90, 100] at 0. Accuracy was lower for Age: (80, 90] at 0.35 and Age: (90, 100] at 0.22 (Supplemental Tables 13-16).

There were statistically significant differences for several subgroups that had less than 10 people in the data set, making results inconclusive. AUROC was lower for Age: (10, 20] (n = 3) at 0.5, Ethnicity: Not Hispanic or Latino, Race: Other, Sex: Male (n = 9) at 0.12, Ethnicity: Not Hispanic or Latino, Race: Native Hawaiian or Other Pacific Islander, Sex: Female (n = 6) at 0.4, and Ethnicity: Not Hispanic or Latino, Race: Native Hawaiian or Other Pacific Islander, Sex: Male (n = 4) at 0.25 (Supplemental Tables 13-16).

### Stanford HM ACP in Hospital Medicine (AUROC/Accuracy)

The Stanford HM ACP model had an AUROC of 0.86 and an accuracy of 0.79. AUROC was lower for Ethnicity: Hispanic or Latino, Race: Other, Sex: Male at 0.38 (Supplemental Tables 17-20).

There were statistically significant differences for several subgroups that had less than 10 people in the data set, making results inconclusive. AUROC was lower for Age: (10, 20] (n = 3) at 0.5, Ethnicity: Not Hispanic or Latino, Race: Black or African American, Sex: Male (n = 9) at 0.25, and Ethnicity: Not Hispanic or Latino, Race: Other, Sex: Male (n = 6) at 0.39 (Supplemental Tables 17-20).

### Model Comparison in Hospital Medicine (AUROC/Accuracy)

The Stanford HM ACP model had a significantly higher accuracy (0.79) than the Epic EOL High Threshold (0.62). There was no statistically significant difference between the models in AUROC.

## Class Balanced Reliability and Fairness Audit

We report the class balanced reliability and fairness audits, i.e. after using Random Oversampling on the class to ensure a 50% prevalence. We compare the class balanced analyses to the original analyses in the main manuscript, which we refer to as “unbalanced.” All values and confidence intervals are listed in Supplemental Tables 27-46.

## *Primary Care*

### Epic EOL Low Threshold in Primary Care - Class Balanced Analysis

Before oversampling, the data set size for the Epic EOL - Low Threshold model in Primary Care was 338 with 68 positive labels. After oversampling the positive labels, the data set size was 540.

The overall prevalence was 0.5. There was significantly higher prevalence for Age: (80, 90] at 0.84 and Age: (90, 100] at 0.95. There was significantly lower prevalence for Age: (40,50] at 0.3, Age: (50,60] at 0.13, Hispanic or Latino patients with Race “Other” at 0.1, and specifically Hispanic or Latino Females with Race “Other” at 0. There were no significant differences in prevalence found by Sex (Supplemental Tables 27-30).

The model flagged 107 patients out of 540 (20%), exhibiting low sensitivity (0.38), high specificity (0.98), and high PPV (0.95). The model also underpredicted events relative to clinicians by a factor of

O/E = 5.3. There was significantly lower sensitivity for Age: (60, 70] at 0.09 and Age: (70, 80] at 0.07. The model also underpredicted events more for Age: (40, 50] by a factor of O/E = 15.0, Age: (60, 70], by a factor of O/E = 19.3, and Age: (70, 80] by a factor of 9.8 (Supplemental Tables 27-30).

For several other groups, there were statistically significant differences in prevalence, performance or O/E, but these subgroups had less than 10 patients to calculate the metric for, making results inconclusive (Supplemental Tables 27-30).

*Comparison of Class Balanced Analysis with Unbalanced Analysis*

Prevalence was higher in the class balanced analysis (0.5) compared with the unbalanced analysis (0.2), due to the random oversampling of the positive label used to generate the class balanced data set. In both the class balanced analysis and the unbalanced analysis, prevalence was significantly higher for Age: (80, 90]. In the class balanced analysis only, prevalence was higher for Age: (90, 100] and lower for Age: (40,50], Age: (50,60], Hispanic patients with Race listed as "Other", and Hispanic female patients with Race listed as "Other". In the unbalanced analysis only, prevalence was lower for Age: (20,30] and Age: (30, 40].

In the class balanced analysis, the Epic EOL - Low Threshold model for Primary Care flagged more patients (20% vs 9%), had a higher PPV (0.95 vs 0.85), and had a higher O/E (5.3 vs 4.1) than in the unbalanced analysis. Otherwise, sensitivity was similar (0.38 vs 0.37), as was specificity (0.98 for both). In both the class balanced analysis and the unbalanced analysis, sensitivity was significantly lower for Age: (60, 70] and Age: (70, 80], and the model underpredicted events more for Age: (60, 70]. In the class balanced analysis only, the model underpredicted events more for Age: (40, 50] and Age: (70, 80].

## *Inpatient Oncology*

### Epic EOL High Threshold in Inpatient Oncology - Class Balanced Analysis

Before oversampling, the data set size for the Epic EOL - High Threshold model in Inpatient Oncology, was 150 with 105 positive labels. After oversampling the negative labels, the data set size was 210.

The overall prevalence was 0.5. There was significantly lower prevalence for Age: (20, 30] at 0.11. There were no significant differences in prevalence by Sex, Ethnicity/Race, and the intersection of Ethnicity/Race and Sex (Supplemental Tables 31-34).

The model flagged 40 patients out of 210 (19%) with a sensitivity of 0.27, specificity of 0.89, and PPV of 0.7. The model predicted fewer events relative to the number of positive clinician labels, with an O/E ratio of 2.4. There was significantly lower sensitivity for Ethnicity: Hispanic or Latino, Race: Other at 0.09; and Ethnicity: Hispanic or Latino, Race: Other, Sex: Male at 0. There was significantly lower specificity for Age: (60, 70] at 0.33 and Ethnicity: Not Hispanic or Latino, Race: White, Sex: Male at 0.59. The model significantly underpredicted events for Ethnicity: Hispanic or Latino, Race: Other at 5.3; and Ethnicity: Hispanic or Latino, Race: Other, Sex: Male at 6.3. Several other subgroups exhibited statistically significant differences in model sensitivity, specificity or O/E, but these subgroups had less than 10 patients to calculate the metric for, making such claims inconclusive. See Supplemental Tables 31-34 for details.

*Comparison of Class Balanced Analysis with Unbalanced Analysis*

Prevalence was lower in the class balanced analysis (0.5) compared with the unbalanced analysis (0.7), due to the random oversampling of the negative label used to generate the class balanced data set. In both

the class balanced analysis and the unbalanced analysis, prevalence was significantly lower for Age: (20, 30].

In the class balanced analysis, Epic EOL High Threshold model in Inpatient Oncology flagged less patients (19% vs 21%), had a lower PPV (0.7 vs 0.88), and had a lower O/E (2.4 vs 3) than in the unbalanced analysis. Otherwise, sensitivity was similar (0.27 vs 0.27), as was specificity (0.89 vs 0.91). In both the class balanced analysis and the unbalanced analysis, sensitivity was significantly lower for Hispanic or Latino patients with Race "Other" and in particular Hispanic or Latino males with Race "Other"; these two groups also both had significant underprediction of events. In the class balanced analysis only, the model had lower specificity for Age: (60, 70] and Ethnicity: Not Hispanic or Latino, Race: White, Sex: Male.

## Stanford HM ACP in Inpatient Oncology - Class Balanced Analysis

Before oversampling, the data set size for the Stanford HM ACP model in Inpatient Oncology was 114 with 79 positive labels. After oversampling the negative labels, the data set size was 158.

The overall prevalence was 0.5. There was significantly lower prevalence for Age: (20, 30] at 0.16, and significantly higher prevalence for Age: (60, 70] at 0.85. There were no significant differences in prevalence amongst Sex, Ethnicitiy/Race, or Ethnicity/Race and Sex (Supplemental Tables 35-38).

The Stanford HM ACP model flagged 105 patients out of 158 (66%) with sensitivity 0.89, specificity 0.56, and PPV 0.67. The model moderately underestimated events relative to clinicians, with an O/E of 1.4. For Age: (40, 50], there was significantly lower specificity at 0.18 and significantly lower PPV at 0.26 (Supplemental Tables 35-38).

Model performance and O/E appeared to differ for some other subgroups, but these subgroups had less than 10 patients to calculate the metric for, making any associated claims inconclusive. See Supplemental Tables 35-38 for details.

*Comparison of Class Balanced Analysis with Unbalanced Analysis*

Prevalence was lower in the class balanced analysis (0.5) compared with the unbalanced analysis (0.69), due to the random oversampling of the negative label used to generate the class balanced data set. In the class balanced analysis only, prevalence was significantly lower for Age: (20, 30] and significantly higher for Age: (60, 70].

In the class balanced analysis, the Stanford HM ACP model in Inpatient Oncology flagged less patients (66% vs 75%), had a lower PPV (0.67 vs 0.82), and had a lower O/E (1.4 vs 1.7) than in the unbalanced analysis. Otherwise, sensitivity was similar (0.89 vs 0.89), as was specificity (0.56 vs 0.57). In the class balanced analysis only, the model had significantly lower specificity and significantly lower PPV for Age: (40, 50].

## Model Comparison in Inpatient Oncology - Class Balanced Analysis

Comparing model performance in Inpatient Oncology, the Stanford HM ACP model flagged more patients (66% vs 19%), had significantly higher sensitivity (0.89 vs 0.27), and exhibited similar PPV (0.67 vs 0.7, 95% confidence intervals overlap). The Epic EOL High Threshold model had significantly higher specificity (0.89 vs 0.56). Comparing model calibration, the Stanford HM ACP model had significantly better calibration in terms of O/E (1.4 vs 2.4).

*Comparison of Class Balanced Analysis with Unbalanced Analysis*

In both the class balanced analysis and unbalanced analysis in Inpatient Oncology, the Stanford HM ACP model flagged more patients, had significantly higher sensitivity, exhibited similar PPV, and had significantly better calibration in terms of O/E compared to the Epic EOL High Threshold model, while the Epic EOL High Threshold model had significantly higher specificity than the Stanford HM ACP model.

## *Hospital Medicine*

### Epic EOL High Threshold in Hospital Medicine - Class Balanced Analysis

The final data set size for the Epic EOL - High Threshold model in Hospital Medicine, was 305 with 133 positive labels. After oversampling the positive labels, the data set size was 344.

The overall prevalence was 0.5. Prevalence did not differ by sex, but was significantly lower for younger patients (0.22 for Age: (20, 30] and 0.15 for Age: (30, 40]). Prevalence was also significantly higher for Non-Hispanic Asian patients (0.76) and, in particular, Non-Hispanic Asian Males (0.79). Prevalence was significantly lower for Hispanic or Latino patients with Race "Other" (0.23) and, in particular, Hispanic or Latino Males of Race "Other" (0.17) Supplemental Tables 39-42.

The model flagged 44 out of 344 patients (13%). The model demonstrated a sensitivity of 0.21, specificity of 0.95, and PPV of 0.82. The model underpredicted events relative to clinicians (O/E ratio of 2.6). The model underestimated events relative to clinicians significantly more for Non-Hispanic White patients (O/E = 3.4) and in particular for Non-Hispanic White Females (O/E = 4.2). Differences in performance and O/E were statistically significant for other subgroups, but these subgroups had less than 10 patients to calculate the metric for, preventing conclusive statements regarding disparate performance. See Supplemental Tables 39-42 for details.

*Comparison of Class Balanced Analysis with Unbalanced Analysis*

Prevalence was higher in the class balanced analysis (0.5) compared with the unbalanced analysis (0.44), due to the random oversampling of the positive label used to generate the class balanced data set. In both the class balanced analysis and the unbalanced analysis, prevalence was significantly lower for Age: (20, 30], Age: (30, 40], Hispanic or Latino patients with Race "Other" and, in particular, Hispanic or Latino Males of Race "Other", and was significantly higher for Non-Hispanic Asian patients. In the class balanced analysis only, prevalence was significantly higher for Non-Hispanic Asian Males. In the unbalanced analysis only, prevalence was significantly higher for older patients (Age: (80, 90], Age: (90, 100])

In the class balanced analysis, Epic EOL High Threshold model in Hospital Medicine flagged more patients (13% vs 11%) and had a higher PPV (0.82 vs 0.76) than in the unbalanced analysis. Otherwise, sensitivity was similar (0.21 vs 0.2), as was specificity (0.95 vs 0.95) and O/E (2.6 vs 2.5). In both the class balanced analysis and the unbalanced analysis, the model underestimated events significantly more for Non-Hispanic White Females. In the class balanced analysis only, the model underestimated events relative to clinicians significantly more for Non-Hispanic White patients. In the unbalanced analysis only, the model had lower sensitivity for Age: (50,60].

## Stanford HM ACP in Hospital Medicine - Class Balanced Analysis

The final data set size for the Stanford HM ACP model in Hospital Medicine, was 225 with 99 positive labels. After oversampling the positive labels, the data set size was 252.

The overall prevalence was 0.5. Prevalence was significantly higher for older patients (0.83 for Age: (80, 90], 0.94 for Age: (90, 100]) and significantly lower for younger patients (0.14 for Age: (30, 40]). Prevalence was also significantly lower for Hispanic or Latino patients with Race “Other” (0.18), and including both Hispanic or Latino Females with Race “Other” (0.22) and Hispanic or Latino Males with Race “Other” (0.12). Prevalence was significantly higher for Non-Hispanic Asian patients (0.73), especially Non-Hispanic Asian Males (0.83) (Supplemental Tables 43-46).

The Stanford HM ACP model flagged 106 out of 252 patients (42%), with sensitivity 0.71, specificity 0.87, and PPV 0.84. Relative to clinicians, the model underestimated events by a factor of O/E = 1.6. For patients Age: (80, 90] and (90, 100], this underestimation was even more substantial with O/E ratios of 2.2 and 2.7, respectively. Specificity was lower (0.57) for Age: (70, 80]. Model performance disparities in other subgroups were inconclusive given they had less than 10 patients to calculate the metric for. See Supplemental Tables 43-46 for details.

*Comparison of Class Balanced Analysis with Unbalanced Analysis*

Prevalence was higher in the class balanced analysis (0.5) compared with the unbalanced analysis (0.44), due to the random oversampling of the positive label used to generate the class balanced data set. In both the class balanced analysis and the unbalanced analysis, Prevalence was significantly higher for older patients (Age: (80, 90], Age: (90, 100]) and Non-Hispanic Asian patients, especially Non-Hispanic Asian Males, and significantly lower for younger patients (Age: (30, 40]) and for Hispanic or Latino patients with Race “Other.” In the class balanced analysis only, prevalence was significantly lower for specifically both Hispanic or Latino Females with Race “Other” and Hispanic or Latino Males with Race “Other”.

In the class balanced analysis, the Stanford HM ACP model in Hospital Medicine flagged more patients (42% vs 38%) and had a higher PPV (0.84 vs 0.8) than in the unbalanced analysis. Otherwise, sensitivity was similar (0.71 vs 0.69), as was specificity (0.87 vs 0.87) and O/E (1.6 vs 1.5). In both the class balanced analysis and the unbalanced analysis, the model had lower specificity for Age: (70, 80] and had a higher underestimation of events for Age: (90, 100]. In the class balanced analysis only, the model had higher underestimation of events for Age: (80, 90. In the unbalanced analysis only, the model had significantly lower PPV for Hispanic or Latino patients with Race “Other.”

## Model Comparison in Hospital Medicine - Class Balanced Analysis

Comparing model performance in Hospital Medicine, relative to the Epic EOL – High Threshold model the Stanford HM ACP model flagged more patients (42% vs 13%), had significantly higher sensitivity (0.71 vs 0.21), similar specificity (0.87 vs 0.95, 95% confidence intervals overlap), and similar PPV (0.84 vs 0.82, 95% confidence intervals overlap). Comparing model calibration, the Stanford HM ACP model had significantly better calibration in O/E (1.6 vs 2.6).

*Comparison of Class Balanced Analysis with Original Analysis*

In both the class balanced analysis and unbalanced analysis in Hospital Medicine, the Stanford HM ACP model flagged more patients, had significantly higher sensitivity, similar specificity, similar PPV, and better calibration in terms of O/E compared to the Epic EOL High Threshold model.

# Supplemental Files:

**Supplemental File 1: Time and Requirements to Generate Reliability/Fairness Audit.**
This file is available as a Google Sheets file at
https://docs.google.com/spreadsheets/d/12qKv8iguvnyO5WWT5vGt4M_Kwv6yRVpCs3Mv-3RVUl4/edit?usp=sharing

# Supplemental Figures:

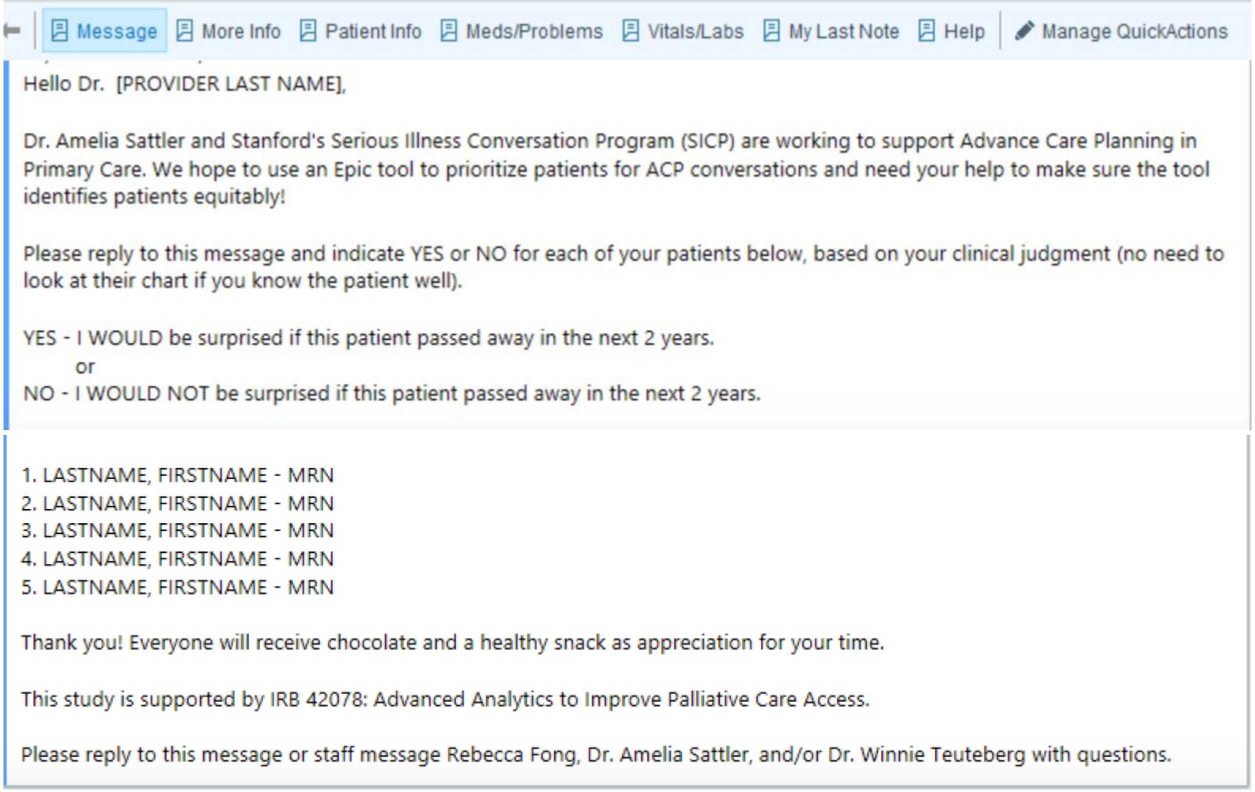
Message | More Info | Patient Info | Meds/Problems | Vitals/Labs | My Last Note | Help | Manage QuickActions

Hello Dr. [PROVIDER LAST NAME],

Dr. Amelia Sattler and Stanford's Serious Illness Conversation Program (SICP) are working to support Advance Care Planning in Primary Care. We hope to use an Epic tool to prioritize patients for ACP conversations and need your help to make sure the tool identifies patients equitably!

Please reply to this message and indicate YES or NO for each of your patients below, based on your clinical judgment (no need to look at their chart if you know the patient well).

YES - I WOULD be surprised if this patient passed away in the next 2 years.
or
NO - I WOULD NOT be surprised if this patient passed away in the next 2 years.

1. LASTNAME, FIRSTNAME - MRN
2. LASTNAME, FIRSTNAME - MRN
3. LASTNAME, FIRSTNAME - MRN
4. LASTNAME, FIRSTNAME - MRN
5. LASTNAME, FIRSTNAME - MRN

Thank you! Everyone will receive chocolate and a healthy snack as appreciation for your time.

This study is supported by IRB 42078: Advanced Analytics to Improve Palliative Care Access.

Please reply to this message or staff message Rebecca Fong, Dr. Amelia Sattler, and/or Dr. Winnie Teuteberg with questions.

**Supplemental Figure 1: Message to Primary Care clinicians soliciting surprise questions, staff message.**

Dear Dr. Wang,

Dr. Samantha Wang and Stanford's Serious Illness Conversation Program (SICP) are working to support Advance Care Planning in Hospital Medicine, using an AI model.

We need your help to make sure the tool identifies patients equitably!

Please reply to this message and indicate YES or NO for each of your patients below, based on your clinical judgment (no need to look at their chart if you know the patient well).

YES - I WOULD be surprised if this patient passed away in 1 year. (GOOD PROGNOSIS)

or

NO - I WOULD NOT be surprised if this patient passed away in 1 year. (POOR PROGNOSIS)

Please also mark an x by the 3 patients you would most prioritize for ACP.

| Name | MRN | Age | Bed | Team | Would I be surprised if pt passed away in 1 yr? | Top 3 for ACP Priority? |
|---|---|---|---|---|---|---|
| [redacted] | | | | MED UNIV 3 | | |
| [redacted] | | | | MED UNIV 3 | | |
| [redacted] | | | | MED UNIV 3 | | |
| [redacted] | | | | MED UNIV 3 | | |
| [redacted] | | | | MED UNIV 3 | | |
| [redacted] | | | | MED UNIV 3 | | |
| [redacted] | | | | MED UNIV 3 | | |
| [redacted] | | | | MED UNIV 3 | | |
| [redacted] | | | | MED UNIV 3 | | |
| [redacted] | | | | MED UNIV 3 | | |
| [redacted] | | | | MED UNIV 3 | | |
| [redacted] | | | | MED UNIV 3 | | |

Thank you! Everyone will receive chocolate as appreciation for your time.

This study is supported by IRB 42078: Advanced Analytics to Improve Palliative Care Access.

Please reply to this message or staff message Jonathan Lu, Dr. Samantha Wang, and/or Dr. Winnie Teuteberg with questions.

**Supplemental Figure 2: Message to Hospital Medicine physicians soliciting surprise questions, email.**

# Supplemental Tables:

## Main Analysis

### *Epic EOL Low Threshold in Primary Care*

| Group | Sample Size | Prevalence (Fraction) | Prevalence [95% CI] | Sensitivity (Fraction) | Sensitivity [95% CI] | Specificity (Fraction) | Specificity [95% CI] | PPV (Fraction) | PPV [95% CI] | O/E (Fraction) | O/E [95% CI] | AUROC | AUROC [95% CI] | Accuracy (Fraction) | Accuracy [95% CI] |
|---|---|---|---|---|---|---|---|---|---|---|---|---|---|---|---|
| Overall | 338 | 0.2 (68/338) | [0.16, 0.25] | 0.37 (25/68) | [0.26, 0.49] | 0.98 (265/270) | [0.97, 1.0] | 0.83 (25/30) | [0.7, 0.98] | 4.1 (68/16.4) | [3.3, 5.1] | 0.91 | [0.87, 0.94] | 0.86 (290/338) | [0.82, 0.9] |
| Sex: Female | 201 | 0.19 (39/201) | [0.14, 0.26] | 0.38 (15/39) | [0.24, 0.53] | 0.98 (158/162) | [0.96, 1.0] | 0.79 (15/19) | [0.63, 0.99] | 4.0 (39/9.8) | [3.0, 5.3] | 0.89 | [0.85, 0.95] | 0.86 (173/201) | [0.82, 0.91] |
| Sex: Male | 137 | 0.21 (29/137) | [0.15, 0.29] | 0.34 (10/29) | [0.15, 0.51] | 0.99 (107/108) | [0.98, 1.01] | 0.91 (10/11) | [0.82, 1.1] | 4.4 (29/6.6) | [3.2, 6.0] | 0.93 | [0.88, 0.97] | 0.85 (117/137) | [0.8, 0.91] |

**Supplemental Table 1: Epic EOL Low Threshold in Primary Care: Reliability and Fairness Audit by Sex.** Significant differences in prevalence, significantly lower performance, or significantly higher O/E are bolded.

| Group | Sample Size | Prevalence (Fraction) | Prevalence [95% CI] | Sensitivity (Fraction) | Sensitivity [95% CI] | Specificity (Fraction) | Specificity [95% CI] | PPV (Fraction) | PPV [95% CI] | O/E (Fraction) | O/E [95% CI] | AUROC | AUROC [95% CI] | Accuracy (Fraction) | Accuracy [95% CI] |
|---|---|---|---|---|---|---|---|---|---|---|---|---|---|---|---|
| Overall | 338 | 0.2 (68/338) | [0.16, 0.25] | 0.37 (25/68) | [0.26, 0.49] | 0.98 (265/270) | [0.97, 1.0] | 0.83 (25/30) | [0.7, 0.98] | 4.1 (68/16.4) | [3.3, 5.1] | 0.91 | [0.87, 0.94] | 0.86 (290/338) | [0.82, 0.9] |
| Age: (10, 20] | 3 | 0.0 (0/3) | [0, 0.71] | nan (0/0) | N/A | 1.0 (1/1) | [1.0, 1.0] | nan (0/0) | N/A | nan (0/0.0) | N/A | nan | N/A | 1.0 (3/3) | [1.0, 1.0] |

| | | | | | | | | | | | | | | | |
|---|---|---|---|---|---|---|---|---|---|---|---|---|---|---|---|
| Age: (20, 30] | 27 | **0.0 (0/27)** | **[0, 0.13]** | nan (0/0) | N/A | 1.0 (1/1) | [1.0, 1.0] | nan (0/0) | N/A | nan (0/0.0) | N/A | nan | N/A | 1.0 (27/27) | [1.0, 1.0] |
| Age: (30, 40] | 61 | **0.0 (0/61)** | **[0, 0.06]** | nan (0/0) | N/A | 1.0 (1/1) | [1.0, 1.0] | nan (0/0) | N/A | 0.0 (0/0.0) | N/A | nan | N/A | 1.0 (61/61) | [1.0, 1.0] |
| Age: (40, 50] | 43 | 0.12 (5/43) | [0.04, 0.25] | 0.2 (1/5) | [-0.27, 0.4] | 1.0 (38/38) | [1.0, 1.0] | 1.0 (1/1) | [1.0, 1.0] | 11.1 (5/0.5) | [4.9, 25.3] | 0.89 | [0.8, 0.99] | 0.91 (39/43) | [0.84, 1.0] |
| Age: (50, 60] | 48 | 0.04 (2/48) | [0.01, 0.14] | **0.0 (0/2)** | **[0.0, 0.0]** | 1.0 (46/46) | [1.0, 1.0] | nan (0/0) | N/A | 4.7 (2/0.4) | [1.2, 18.1] | 0.78 | [0.57, 1.03] | 0.96 (46/48) | [0.92, 1.02] |
| Age: (60, 70] | 51 | 0.2 (10/51) | [0.1, 0.33] | **0.1 (1/10)** | **[-0.13, 0.2]** | 1.0 (41/41) | [1.0, 1.0] | 1.0 (1/1) | [1.0, 1.0] | **9.3 (10/1.1)** | **[5.3, 16.1]** | **0.66** | **[0.44, 0.88]** | **0.82 (42/51)** | **[0.73, 0.94]** |
| Age: (70, 80] | 51 | 0.29 (15/51) | [0.17, 0.44] | **0.07 (1/15)** | **[-0.09, 0.13]** | 0.97 (35/36) | [0.94, 1.03] | 0.5 (1/2) | [0.0, 1.0] | 6.3 (15/2.4) | [4.1, 9.6] | 0.8 | [0.68, 0.93] | 0.71 (36/51) | [0.59, 0.84] |
| Age: (80, 90] | 33 | **0.55 (18/33)** | **[0.36, 0.72]** | 0.39 (7/18) | [0.15, 0.61] | 0.87 (13/15) | [0.73, 1.07] | 0.78 (7/9) | [0.56, 1.11] | 3.4 (18/5.3) | [2.5, 4.6] | 0.68 | [0.5, 0.88] | 0.61 (20/33) | [0.45, 0.76] |
| Age: (90, 100] | 19 | **0.84 (16/19)** | **[0.6, 0.97]** | 0.81 (13/16) | [0.62, 1.0] | **0.33 (1/3)** | **[-0.33, 0.67]** | 0.87 (13/15) | [0.73, 1.05] | 2.7 (16/5.9) | [2.2, 3.3] | 0.73 | [0.46, 1.12] | 0.74 (14/19) | [0.58, 0.95] |
| Age: (100, 110] | 2 | 1.0 (2/2) | [0.16, 1] | 1.0 (1/1) | [1.0, 1.0] | nan (0/0) | N/A | 1.0 (1/1) | [1.0, 1.0] | 2.4 (2/0.8) | [2.4, 2.4] | nan | N/A | 1.0 (2/2) | [1.0, 1.0] |

**Supplemental Table 2: Epic EOL Low Threshold in Primary Care: Reliability and Fairness Audit by Age.** Significant differences in prevalence, significantly lower performance, or significantly higher O/E are bolded.

| Group | Sample Size | Prevalence (Fraction) | Prevalence [95% CI] | Sensitivity (Fraction) | Sensitivity [95% CI] | Specificity (Fraction) | Specificity [95% CI] | PPV (Fraction ) | PPV [95% CI] | O/E (Fraction) | O/E [95% CI] | AUROC | AUROC [95% CI] | Accuracy (Fraction) | Accuracy [95% CI] |
|---|---|---|---|---|---|---|---|---|---|---|---|---|---|---|---|
| Overall | 338 | 0.2 (68/338) | [0.16, 0.25] | 0.37 (25/68) | [0.26, 0.49] | 0.98 (265/270) | [0.97, 1.0] | 0.83 (25/30) | [0.7, 0.98] | 4.1 (68/16.4) | [3.3, 5.1] | 0.91 | [0.87, 0.94] | 0.86 (290/338) | [0.82, 0.9] |
| Ethnicity: Not Hispanic or Latino, | 154 | 0.25 (39/154) | [0.19, 0.33] | 0.44 (17/39) | [0.28, 0.59] | 0.97 (112/115) | [0.95, 1.01] | 0.85 (17/20) | [0.7, 1.01] | 4.0 (39/9.6) | [3.1, 5.3] | 0.93 | [0.9, 0.98] | 0.84 (129/154) | [0.79, 0.9] |

| | | | | | | | | | | | | | | | |
|---|---|---|---|---|---|---|---|---|---|---|---|---|---|---|---|
| Race: White | | | | | | | | | | | | | | | |
| Ethnicity: Not Hispanic or Latino, Race: Asian | 103 | 0.19 (20/103) | [0.12, 0.28] | 0.3 (6/20) | [0.07, 0.49] | 0.99 (82/83) | [0.98, 1.01] | 0.86 (6/7) | [0.71, 1.21] | 4.7 (20/4.2) | [3.2, 7.0] | 0.89 | [0.83, 0.97] | 0.85 (88/103) | [0.79, 0.92] |
| Ethnicity: Not Hispanic or Latino, Race: Other | 24 | 0.25 (6/24) | [0.1, 0.47] | 0.33 (2/6) | [-0.13, 0.67] | 0.94 (17/18) | [0.89, 1.08] | 0.67 (2/3) | [0.33, 1.33] | 3.1 (6/1.9) | [1.6, 6.2] | 0.87 | [0.74, 1.05] | 0.79 (19/24) | [0.62, 0.96] |
| Ethnicity: Hispanic or Latino, Race: Other | 20 | 0.05 (1/20) | [0.0, 0.25] | **0.0 (0/1)** | **[0.0, 0.0]** | 1.0 (19/19) | [1.0, 1.0] | nan (0/0) | N/A | 4.2 (1/0.2) | [0.6, 28.1] | 0.87 | [0.74, 1.03] | 0.95 (19/20) | [0.9, 1.05] |
| Ethnicity: Not Hispanic or Latino, Race: Black or African American | 9 | 0.0 (0/9) | [0, 0.34] | nan (0/0) | N/A | 1.0 (9/9) | [1.0, 1.0] | nan (0/0) | N/A | 0.0 (0/0.2) | N/A | nan | N/A | 1.0 (9/9) | [1.0, 1.0] |
| Ethnicity: Hispanic or Latino, Race: White | 5 | 0.0 (0/5) | [0, 0.52] | nan (0/0) | N/A | 1.0 (5/5) | [1.0, 1.0] | nan (0/0) | N/A | 0.0 (0/0.0) | N/A | nan | N/A | 1.0 (5/5) | [1.0, 1.0] |
| Ethnicity: Not Hispanic or Latino, Race: Native Hawaiian or Other Pacific Islander | 3 | 0.33 (1/3) | [0.01, 0.91] | **0.0 (0/1)** | **[0.0, 0.0]** | 1.0 (2/2) | [1.0, 1.0] | nan (0/0) | N/A | **50.0 (1/0.0)** | **[10.1, 247.7]** | **0.75** | **[0.5, 1.0]** | **0.67 (2/3)** | **[0.33, 1.33]** |
| Ethnicity: Not Hispanic or Latino, Race: Unknown | 3 | 0.0 (0/3) | [0, 0.71] | nan (0/0) | N/A | 1.0 (3/3) | [1.0, 1.0] | nan (0/0) | N/A | 0.0 (0/0.0) | N/A | nan | N/A | 1.0 (3/3) | [1.0, 1.0] |
| Ethnicity: Hispanic or Latino, Race: Asian | 2 | 0.0 (0/2) | [0, 0.84] | nan (0/0) | N/A | 1.0 (2/2) | [1.0, 1.0] | nan (0/0) | N/A | nan (0/0.0) | N/A | nan | N/A | 1.0 (2/2) | [1.0, 1.0] |
| Ethnicity: Not Hispanic or Latino, Race: American Indian or Alaska Native | 1 | 0.0 (0/1) | [0, 0.98] | nan (0/0) | N/A | 1.0 (1/1) | [1.0, 1.0] | nan (0/0) | N/A | 0.0 (0/0.0) | N/A | nan | N/A | 1.0 (1/1) | [1.0, 1.0] |
| Ethnicity: Not Hispanic or Latino, | 1 | 0.0 (0/1) | [0, 0.98] | nan (0/0) | N/A | 1.0 (1/1) | [1.0, 1.0] | nan (0/0) | N/A | nan (0/0.0) | N/A | nan | N/A | 1.0 (1/1) | [1.0, 1.0] |

| | | | | | | | | | | | | | | | |
|---|---|---|---|---|---|---|---|---|---|---|---|---|---|---|---|
| Race: Patient Refused | | | | | | | | | | | | | | | |

**Supplemental Table 3: Epic EOL Low Threshold in Primary Care: Reliability and Fairness Audit by Ethnicity/Race.** Significant differences in prevalence, significantly lower performance, or significantly higher O/E are bolded.

| Group | Sample Size | Prevalence (Fraction) | Prevalence [95% CI] | Sensitivity (Fraction) | Sensitivity [95% CI] | Specificity (Fraction) | Specificity [95% CI] | PPV (Fraction) | PPV [95% CI] | O/E (Fraction) | O/E [95% CI] | AUROC | AUROC [95% CI] | Accuracy (Fraction) | Accuracy [95% CI] |
|---|---|---|---|---|---|---|---|---|---|---|---|---|---|---|---|
| Overall | 338 | 0.2 (68/338) | [0.16, 0.25] | 0.37 (25/68) | [0.26, 0.49] | 0.98 (265/270) | [0.97, 1.0] | 0.83 (25/30) | [0.7, 0.98] | 4.1 (68/16.4) | [3.3, 5.1] | 0.91 | [0.87, 0.94] | 0.86 (290/338) | [0.82, 0.9] |
| Ethnicity: Not Hispanic or Latino, Race: White, Sex: Female | 90 | 0.24 (22/90) | [0.16, 0.35] | 0.5 (11/22) | [0.29, 0.71] | 0.97 (66/68) | [0.94, 1.01] | 0.85 (11/13) | [0.69, 1.07] | 4.0 (22/5.5) | [2.8, 5.8] | 0.94 | [0.89, 1.0] | 0.86 (77/90) | [0.79, 0.93] |
| Ethnicity: Not Hispanic or Latino, Race: White, Sex: Male | 64 | 0.27 (17/64) | [0.16, 0.39] | 0.35 (6/17) | [0.12, 0.56] | 0.98 (46/47) | [0.96, 1.03] | 0.86 (6/7) | [0.71, 1.14] | 4.1 (17/4.1) | [2.7, 6.2] | 0.92 | [0.86, 0.99] | 0.81 (52/64) | [0.72, 0.92] |
| Ethnicity: Not Hispanic or Latino, Race: Asian, Sex: Female | 61 | 0.18 (11/61) | [0.09, 0.3] | 0.27 (3/11) | [-0.01, 0.55] | 0.98 (49/50) | [0.96, 1.02] | 0.75 (3/4) | [0.5, 1.5] | 4.2 (11/2.6) | [2.4, 7.1] | 0.85 | [0.76, 0.98] | 0.85 (52/61) | [0.77, 0.95] |
| Ethnicity: Not Hispanic or Latino, Race: Asian, Sex: Male | 42 | 0.21 (9/42) | [0.1, 0.37] | 0.33 (3/9) | [0.0, 0.67] | 1.0 (33/33) | [1.0, 1.0] | 1.0 (3/3) | [1.0, 1.0] | 5.6 (9/1.6) | [3.2, 10.0] | 0.94 | [0.88, 1.02] | 0.86 (36/42) | [0.76, 0.98] |
| Ethnicity: Not Hispanic or Latino, Race: Other, Sex: Female | 17 | 0.24 (4/17) | [0.07, 0.5] | 0.25 (1/4) | [-0.4, 0.5] | 0.92 (12/13) | [0.85, 1.1] | 0.5 (1/2) | [0.0, 1.0] | 3.0 (4/1.3) | [1.3, 7.0] | 0.88 | [0.77, 1.08] | 0.76 (13/17) | [0.59, 1.0] |

| | | | | | | | | | | | | | | | |
|---|---|---|---|---|---|---|---|---|---|---|---|---|---|---|---|
| Ethnicity: Hispanic or Latino, Race: Other, Sex: Female | 11 | 0.0 (0/11) | [0, 0.28] | nan (0/0) | N/A | 1.0 (11/11) | [1.0, 1.0] | nan (0/0) | N/A | 0.0 (0/0.1) | N/A | nan | N/A | 1.0 (11/11) | [1.0, 1.0] |
| Ethnicity: Hispanic or Latino, Race: Other, Sex: Male | 9 | 0.11 (1/9) | [0.0, 0.48] | **0.0 (0/1)** | **[0.0, 0.0]** | 1.0 (8/8) | [1.0, 1.0] | nan (0/0) | N/A | 7.1 (1/0.1) | [1.1, 45.3] | 0.75 | [0.5, 1.07] | 0.89 (8/9) | [0.78, 1.11] |
| Ethnicity: Not Hispanic or Latino, Race: Other, Sex: Male | 7 | 0.29 (2/7) | [0.04, 0.71] | 0.5 (1/2) | [0.0, 1.0] | 1.0 (5/5) | [1.0, 1.0] | 1.0 (1/1) | [1.0, 1.0] | 3.4 (2/0.6) | [1.1, 11.1] | 0.8 | [0.6, 1.35] | 0.86 (6/7) | [0.71, 1.14] |
| Ethnicity: Not Hispanic or Latino, Race: Black or African American, Sex: Female | 7 | 0.0 (0/7) | [0, 0.41] | nan (0/0) | N/A | 1.0 (7/7) | [1.0, 1.0] | nan (0/0) | N/A | 0.0 (0/0.2) | N/A | nan | N/A | 1.0 (7/7) | [1.0, 1.0] |
| Ethnicity: Hispanic or Latino, Race: White, Sex: Male | 4 | 0.0 (0/4) | [0, 0.6] | nan (0/0) | N/A | 1.0 (4/4) | [1.0, 1.0] | nan (0/0) | N/A | 0.0 (0/0.0) | N/A | nan | N/A | 1.0 (4/4) | [1.0, 1.0] |
| Ethnicity: Not Hispanic or Latino, Race: Native Hawaiian or Other Pacific Islander, Sex: Female | 3 | 0.33 (1/3) | [0.01, 0.91] | **0.0 (0/1)** | **[0.0, 0.0]** | 1.0 (2/2) | [1.0, 1.0] | nan (0/0) | N/A | **50.0 (1/0.0)** | **[10.1, 247.7]** | **0.75** | **[0.5, 1.0]** | **0.67 (2/3)** | **[0.33, 1.33]** |
| Ethnicity: Not Hispanic or Latino, Race: Black or African American, Sex: Male | 2 | 0.0 (0/2) | [0, 0.84] | nan (0/0) | N/A | 1.0 (2/2) | [1.0, 1.0] | nan (0/0) | N/A | 0.0 (0/0.0) | N/A | nan | N/A | 1.0 (2/2) | [1.0, 1.0] |
| Ethnicity: Not Hispanic or | 2 | 0.0 (0/2) | [0, 0.84] | nan (0/0) | N/A | 1.0 (2/2) | [1.0, 1.0] | nan (0/0) | N/A | 0.0 (0/0.0) | N/A | nan | N/A | 1.0 (2/2) | [1.0, 1.0] |

| | | | | | | | | | | | | | | | |
|---|---|---|---|---|---|---|---|---|---|---|---|---|---|---|---|
| Latino, Race: Unknown, Sex: Male | | | | | | | | | | | | | | | |
| Ethnicity: Hispanic or Latino, Race: Asian, Sex: Female | 2 | 0.0 (0/2) | [0, 0.84] | nan (0/0) | N/A | 1.0 (2/2) | [1.0, 1.0] | nan (0/0) | N/A | nan (0/0.0) | N/A | nan | N/A | 1.0 (2/2) | [1.0, 1.0] |
| Ethnicity: Hispanic or Latino, Race: White, Sex: Female | 1 | 0.0 (0/1) | [0, 0.98] | nan (0/0) | N/A | 1.0 (1/1) | [1.0, 1.0] | nan (0/0) | N/A | nan (0/0.0) | N/A | nan | N/A | 1.0 (1/1) | [1.0, 1.0] |
| Ethnicity: Not Hispanic or Latino, Race: Unknown, Sex: Female | 1 | 0.0 (0/1) | [0, 0.98] | nan (0/0) | N/A | 1.0 (1/1) | [1.0, 1.0] | nan (0/0) | N/A | nan (0/0.0) | N/A | nan | N/A | 1.0 (1/1) | [1.0, 1.0] |
| Ethnicity: Not Hispanic or Latino, Race: American Indian or Alaska Native, Sex: Male | 1 | 0.0 (0/1) | [0, 0.98] | nan (0/0) | N/A | 1.0 (1/1) | [1.0, 1.0] | nan (0/0) | N/A | 0.0 (0/0.0) | N/A | nan | N/A | 1.0 (1/1) | [1.0, 1.0] |
| Ethnicity: Not Hispanic or Latino, Race: Patient Refused, Sex: Male | 1 | 0.0 (0/1) | [0, 0.98] | nan (0/0) | N/A | 1.0 (1/1) | [1.0, 1.0] | nan (0/0) | N/A | nan (0/0.0) | N/A | nan | N/A | 1.0 (1/1) | [1.0, 1.0] |

**Supplemental Table 4: Epic EOL Low Threshold in Primary Care: Reliability and Fairness Audit by Ethnicity/Race and Sex.** Significant differences in prevalence, significantly lower performance, or significantly higher O/E are bolded.

*Epic EOL High Threshold in Inpatient Oncology*

| Group | Sample Size | Prevalence (Fraction) | Prevalence [95% CI] | Sensitivity (Fraction) | Sensitivity [95% CI] | Specificity (Fraction) | Specificity [95% CI] | PPV (Fraction) | PPV [95% CI] | O/E (Fraction) | O/E [95% CI] | AUROC | AUROC [95% CI] | Accuracy (Fraction) | Accuracy [95% CI] |
|---|---|---|---|---|---|---|---|---|---|---|---|---|---|---|---|
| Overall | 150 | 0.7 (105/150) | [0.62, 0.77] | 0.27 (28/105) | [0.18, 0.34] | 0.91 (41/45) | [0.84, 1.0] | 0.88 (28/32) | [0.78, 1.01] | 3.0 (105/34.8) | [2.7, 3.4] | 0.7 | [0.61, 0.79] | 0.46 (69/150) | [0.38, 0.53] |
| Sex: Female | 61 | 0.72 (44/61) | [0.59, 0.83] | 0.27 (12/44) | [0.14, 0.4] | 1.0 (17/17) | [1.0, 1.0] | 1.0 (12/12) | [1.0, 1.0] | 3.5 (44/12.7) | [3.0, 4.0] | 0.74 | [0.62, 0.88] | 0.48 (29/61) | [0.34, 0.61] |
| Sex: Male | 89 | 0.69 (61/89) | [0.58, 0.78] | 0.26 (16/61) | [0.15, 0.37] | 0.86 (24/28) | [0.75, 0.99] | 0.8 (16/20) | [0.65, 1.0] | 2.8 (61/22.1) | [2.4, 3.2] | 0.67 | [0.54, 0.79] | 0.45 (40/89) | [0.35, 0.55] |

**Supplemental Table 5: Epic EOL High Threshold in Inpatient Oncology: Reliability and Fairness Audit by Sex.** Prevalence, performance and calibration is presented for the overall cohort and for subgroups with significant differences in prevalence, significantly lower performance, or significantly higher O/E (bolded).

| Group | Sample Size | Prevalence (Fraction) | Prevalence [95% CI] | Sensitivity (Fraction) | Sensitivity [95% CI] | Specificity (Fraction) | Specificity [95% CI] | PPV (Fraction) | PPV [95% CI] | O/E (Fraction) | O/E [95% CI] | AUROC | AUROC [95% CI] | Accuracy (Fraction) | Accuracy [95% CI] |
|---|---|---|---|---|---|---|---|---|---|---|---|---|---|---|---|
| Overall | 150 | 0.7 (105/150) | [0.62, 0.77] | 0.27 (28/105) | [0.18, 0.34] | 0.91 (41/45) | [0.84, 1.0] | 0.88 (28/32) | [0.78, 1.01] | 3.0 (105/34.8) | [2.7, 3.4] | 0.7 | [0.61, 0.79] | 0.46 (69/150) | [0.38, 0.53] |
| Age: (20, 30] | 13 | **0.23 (3/13)** | **[0.05, 0.54]** | **0.0 (0/3)** | **[0.0, 0.0]** | 1.0 (10/10) | [1.0, 1.0] | nan (0/0) | N/A | 5.0 (3/0.6) | [1.9, 13.5] | 0.67 | [0.33, 1.2] | 0.77 (10/13) | [0.54, 1.0] |
| Age: (30, 40] | 14 | 0.57 (8/14) | [0.29, 0.82] | **0.0 (0/8)** | **[0.0, 0.0]** | 1.0 (6/6) | [1.0, 1.0] | nan (0/0) | N/A | **7.5 (8/1.1)** | **[4.8, 11.9]** | **0.47** | **[0.17, 0.74]** | **0.43 (6/14)** | **[0.14, 0.64]** |
| Age: (40, 50] | 14 | 0.57 (8/14) | [0.29, 0.82] | 0.12 (1/8) | [-0.15, 0.25] | 1.0 (6/6) | [1.0, 1.0] | 1.0 (1/1) | [1.0, 1.0] | 3.8 (8/2.1) | [2.4, 5.9] | 0.81 | [0.62, 1.12] | 0.5 (7/14) | [0.21, 0.79] |
| Age: (50, 60] | 27 | 0.67 (18/27) | [0.46, 0.83] | 0.28 (5/18) | [0.06, 0.46] | 1.0 (9/9) | [1.0, 1.0] | 1.0 (5/5) | [1.0, 1.0] | 2.8 (18/6.5) | [2.1, 3.6] | 0.83 | [0.69, 1.01] | 0.52 (14/27) | [0.33, 0.7] |
| Age: (60, 70] | 34 | 0.85 (29/34) | [0.69, 0.95] | 0.24 (7/29) | [0.07, 0.39] | **0.4 (2/5)** | **[-0.2, 0.8]** | 0.7 (7/10) | [0.4, 1.02] | 3.0 (29/9.8) | [2.6, 3.4] | 0.34 | [-0.02, 0.63] | 0.26 (9/34) | [0.12, 0.41] |
| Age: (70, 80] | 31 | 0.77 (24/31) | [0.59, 0.9] | 0.38 (9/24) | [0.18, 0.56] | 0.86 (6/7) | [0.71, 1.14] | 0.9 (9/10) | [0.8, 1.13] | 2.8 (24/8.4) | [2.4, 3.4] | 0.58 | [0.32, 0.85] | 0.48 (15/31) | [0.32, 0.65] |
| Age: (80, 90] | 15 | 0.87 (13/15) | [0.6, 0.98] | 0.38 (5/13) | [0.13, 0.64] | 1.0 (2/2) | [1.0, 1.0] | 1.0 (5/5) | [1.0, 1.0] | 2.5 (13/5.2) | [2.0, 3.0] | 0.73 | [0.5, 1.1] | 0.47 (7/15) | [0.2, 0.73] |

| | | | | | | | | | | | | | | | |
|---|---|---|---|---|---|---|---|---|---|---|---|---|---|---|---|
| Age: (90, 100] | 2 | 1.0 (2/2) | [0.16, 1] | 0.5 (1/2) | [0.0, 1.0] | nan (0/0) | N/A | 1.0 (1/1) | [1.0, 1.0] | 1.8 (2/1.1) | [1.8, 1.8] | nan | N/A | 0.5 (1/2) | [0.0, 1.0] |

**Supplemental Table 6: Epic EOL High Threshold in Inpatient Oncology: Reliability and Fairness Audit by Age.** Significant differences in prevalence, significantly lower performance, or significantly higher O/E are bolded.

| Group | Sample Size | Prevalence (Fraction) | Prevalence [95% CI] | Sensitivity (Fraction) | Sensitivity [95% CI] | Specificity (Fraction) | Specificity [95% CI] | PPV (Fraction) | PPV [95% CI] | O/E (Fraction) | O/E [95% CI] | AUROC | AUROC [95% CI] | Accuracy (Fraction) | Accuracy [95% CI] |
|---|---|---|---|---|---|---|---|---|---|---|---|---|---|---|---|
| Overall | 150 | 0.7 (105/150) | [0.62, 0.77] | 0.27 (28/105) | [0.18, 0.34] | 0.91 (41/45) | [0.84, 1.0] | 0.88 (28/32) | [0.78, 1.01] | 3.0 (105/34.8) | [2.7, 3.4] | 0.7 | [0.61, 0.79] | 0.46 (69/150) | [0.38, 0.53] |
| Ethnicity: Not Hispanic or Latino, Race: White | 57 | 0.67 (38/57) | [0.53, 0.79] | 0.24 (9/38) | [0.1, 0.37] | 0.84 (16/19) | [0.68, 1.03] | 0.75 (9/12) | [0.5, 1.04] | 2.7 (38/14.1) | [2.2, 3.2] | 0.65 | [0.49, 0.82] | 0.44 (25/57) | [0.32, 0.58] |
| Ethnicity: Not Hispanic or Latino, Race: Asian | 38 | 0.82 (31/38) | [0.66, 0.92] | 0.39 (12/31) | [0.21, 0.56] | 1.0 (7/7) | [1.0, 1.0] | 1.0 (12/12) | [1.0, 1.0] | 2.6 (31/11.9) | [2.2, 3.0] | 0.83 | [0.72, 0.97] | 0.5 (19/38) | [0.34, 0.66] |
| Ethnicity: Hispanic or Latino, Race: Other | 30 | 0.73 (22/30) | [0.54, 0.88] | **0.09 (2/22)** | **[-0.05, 0.18]** | 1.0 (8/8) | [1.0, 1.0] | 1.0 (2/2) | [1.0, 1.0] | **6.9 (22/3.2)** | **[5.6, 8.6]** | **0.63** | **[0.4, 0.89]** | **0.33 (10/30)** | **[0.17, 0.5]** |
| Ethnicity: Not Hispanic or Latino, Race: Other | 14 | 0.64 (9/14) | [0.35, 0.87] | 0.33 (3/9) | [0.0, 0.59] | 1.0 (5/5) | [1.0, 1.0] | 1.0 (3/3) | [1.0, 1.0] | 2.9 (9/3.1) | [2.0, 4.3] | 0.96 | [0.91, 1.11] | 0.57 (8/14) | [0.36, 0.86] |
| Ethnicity: Not Hispanic or Latino, Race: Black or African American | 5 | 0.4 (2/5) | [0.05, 0.85] | 0.5 (1/2) | [0.0, 1.0] | 0.67 (2/3) | [0.33, 1.33] | 0.5 (1/2) | [0.0, 1.0] | 1.3 (2/1.5) | [0.4, 3.8] | 0.83 | [0.67, 1.42] | 0.6 (3/5) | [0.2, 1.0] |
| Ethnicity: Hispanic or Latino, Race: White | 3 | 0.33 (1/3) | [0.01, 0.91] | **0.0 (0/1)** | **[0.0, 0.0]** | 1.0 (2/2) | [1.0, 1.0] | nan (0/0) | N/A | 2.9 (1/0.3) | [0.6, 14.6] | 0 | [0.0, 0.0] | 0.67 (2/3) | [0.33, 1.33] |
| Ethnicity: Hispanic or Latino, Race: American Indian or Alaska Native | 1 | 0.0 (0/1) | [0, 0.98] | nan (0/0) | N/A | 1.0 (1/1) | [1.0, 1.0] | nan (0/0) | N/A | 0.0 (0/0.0) | N/A | nan | N/A | 1.0 (1/1) | [1.0, 1.0] |

| | | | | | | | | | | | | | | | |
|---|---|---|---|---|---|---|---|---|---|---|---|---|---|---|---|
| Ethnicity: Not Hispanic or Latino, Race: American Indian or Alaska Native | 1 | 1.0 (1/1) | [0.03, 1] | 1.0 (1/1) | [1.0, 1.0] | nan (0/0) | N/A | 1.0 (1/1) | [1.0, 1.0] | 1.6 (1/0.6) | [1.6, 1.6] | nan | N/A | 1.0 (1/1) | [1.0, 1.0] |

**Supplemental Table 7: Epic EOL High Threshold in Inpatient Oncology: Reliability and Fairness Audit by Ethnicity/Race.** Significant differences in prevalence, significantly lower performance, or significantly higher O/E are bolded.

| Group | Sample Size | Prevalence (Fraction) | Prevalence [95% CI] | Sensitivity (Fraction) | Sensitivity [95% CI] | Specificity (Fraction) | Specificity [95% CI] | PPV (Fraction) | PPV [95% CI] | O/E (Fraction) | O/E [95% CI] | AUROC | AUROC [95% CI] | Accuracy (Fraction) | Accuracy [95% CI] |
|---|---|---|---|---|---|---|---|---|---|---|---|---|---|---|---|
| Overall | 150 | 0.7 (105/150) | [0.62, 0.77] | 0.27 (28/105) | [0.18, 0.34] | 0.91 (41/45) | [0.84, 1.0] | 0.88 (28/32) | [0.78, 1.01] | 3.0 (105/34.8) | [2.7, 3.4] | 0.7 | [0.61, 0.79] | 0.46 (69/150) | [0.38, 0.53] |
| Ethnicity: Not Hispanic or Latino, Race: White, Sex: Male | 32 | 0.69 (22/32) | [0.5, 0.84] | 0.23 (5/22) | [0.05, 0.38] | 0.7 (7/10) | [0.4, 1.02] | 0.62 (5/8) | [0.25, 1.0] | 2.7 (22/8.3) | [2.1, 3.4] | 0.58 | [0.32, 0.86] | 0.38 (12/32) | [0.22, 0.53] |
| Ethnicity: Not Hispanic or Latino, Race: White, Sex: Female | 25 | 0.64 (16/25) | [0.43, 0.82] | 0.25 (4/16) | [0.03, 0.44] | 1.0 (9/9) | [1.0, 1.0] | 1.0 (4/4) | [1.0, 1.0] | 2.8 (16/5.8) | [2.1, 3.7] | 0.75 | [0.57, 0.97] | 0.52 (13/25) | [0.32, 0.72] |
| Ethnicity: Not Hispanic or Latino, Race: Asian, Sex: Male | 23 | 0.83 (19/23) | [0.61, 0.95] | 0.37 (7/19) | [0.15, 0.58] | 1.0 (4/4) | [1.0, 1.0] | 1.0 (7/7) | [1.0, 1.0] | 2.4 (19/8.0) | [2.0, 2.9] | 0.84 | [0.69, 1.04] | 0.48 (11/23) | [0.26, 0.7] |
| Ethnicity: Hispanic or Latino, Race: Other, Sex: Male | 17 | 0.76 (13/17) | [0.5, 0.93] | **0.0 (0/13)** | **[0.0, 0.0]** | 1.0 (4/4) | [1.0, 1.0] | nan (0/0) | N/A | **9.0 (13/1.4)** | **[6.9, 11.8]** | **0.59** | **[0.22, 1.04]** | **0.24 (4/17)** | **[0.0, 0.41]** |
| Ethnicity: Not Hispanic or Latino, Race: Asian, Sex: Female | 15 | 0.8 (12/15) | [0.52, 0.96] | 0.42 (5/12) | [0.13, 0.68] | 1.0 (3/3) | [1.0, 1.0] | 1.0 (5/5) | [1.0, 1.0] | 3.1 (12/3.9) | [2.4, 3.9] | 0.83 | [0.67, 1.09] | 0.53 (8/15) | [0.27, 0.8] |
| Ethnicity: Hispanic or Latino, Race: Other, Sex: Female | 13 | 0.69 (9/13) | [0.39, 0.91] | 0.22 (2/9) | [-0.06, 0.44] | 1.0 (4/4) | [1.0, 1.0] | 1.0 (2/2) | [1.0, 1.0] | **5.2 (9/1.7)** | **[3.6, 7.4]** | **0.69** | **[0.39, 1.12]** | **0.46 (6/13)** | **[0.23, 0.77]** |

| | | | | | | | | | | | | | | | |
|---|---|---|---|---|---|---|---|---|---|---|---|---|---|---|---|
| Ethnicity: Not Hispanic or Latino, Race: Other, Sex: Male | 9 | 0.44 (4/9) | [0.14, 0.79] | 0.5 (2/4) | [0.0, 1.0] | 1.0 (5/5) | [1.0, 1.0] | 1.0 (2/2) | [1.0, 1.0] | 1.9 (4/2.1) | [0.9, 4.0] | 0.95 | [0.9, 1.16] | 0.78 (7/9) | [0.56, 1.11] |
| Ethnicity: Not Hispanic or Latino, Race: Other, Sex: Female | 5 | 1.0 (5/5) | [0.48, 1] | 0.2 (1/5) | [-0.2, 0.4] | nan (0/0) | N/A | 1.0 (1/1) | [1.0, 1.0] | **4.9 (5/1.0)** | **[4.9, 4.9]** | **nan** | **N/A** | **0.2 (1/5)** | **[-0.2, 0.4]** |
| Ethnicity: Not Hispanic or Latino, Race: Black or African American, Sex: Male | 3 | 0.33 (1/3) | [0.01, 0.91] | 1.0 (1/1) | [1.0, 1.0] | 0.5 (1/2) | [0.0, 1.0] | 0.5 (1/2) | [0.0, 1.0] | 0.8 (1/1.3) | [0.2, 3.8] | 1 | [1.0, 1.0] | 0.67 (2/3) | [0.33, 1.33] |
| Ethnicity: Not Hispanic or Latino, Race: Black or African American, Sex: Female | 2 | 0.5 (1/2) | [0.01, 0.99] | **0.0 (0/1)** | **[0.0, 0.0]** | 1.0 (1/1) | [1.0, 1.0] | nan (0/0) | N/A | 4.2 (1/0.2) | [1.0, 16.7] | 1 | [1.0, 1.0] | 0.5 (1/2) | [0.0, 1.0] |
| Ethnicity: Hispanic or Latino, Race: White, Sex: Male | 2 | 0.0 (0/2) | [0, 0.84] | nan (0/0) | N/A | 1.0 (1/1) | [1.0, 1.0] | nan (0/0) | N/A | 0.0 (0/0.3) | N/A | nan | N/A | 1.0 (2/2) | [1.0, 1.0] |
| Ethnicity: Hispanic or Latino, Race: White, Sex: Female | 1 | 1.0 (1/1) | [0.03, 1] | **0.0 (0/1)** | **[0.0, 0.0]** | nan (0/0) | N/A | nan (0/0) | N/A | **inf (1/0.0)** | **[inf, inf]** | **nan** | **N/A** | **0.0 (0/1)** | **[0.0, 0.0]** |
| Ethnicity: Hispanic or Latino, Race: American Indian or Alaska Native, Sex: Male | 1 | 0.0 (0/1) | [0, 0.98] | nan (0/0) | N/A | 1.0 (1/1) | [1.0, 1.0] | nan (0/0) | N/A | 0.0 (0/0.0) | N/A | nan | N/A | 1.0 (1/1) | [1.0, 1.0] |
| Ethnicity: Not Hispanic or Latino, Race: American Indian or Alaska Native, Sex: Male | 1 | 1.0 (1/1) | [0.03, 1] | 1.0 (1/1) | [1.0, 1.0] | nan (0/0) | N/A | 1.0 (1/1) | [1.0, 1.0] | 1.6 (1/0.6) | [1.6, 1.6] | nan | N/A | 1.0 (1/1) | [1.0, 1.0] |

**Supplemental Table 8: Epic EOL High Threshold in Inpatient Oncology: Reliability and Fairness Audit by Ethnicity/Race and Sex.** Significant differences in prevalence, significantly lower performance, or significantly higher O/E are bolded.

*Stanford HM ACP in Inpatient Oncology*

| Group | Sample Size | Prevalence (Fraction) | Prevalence [95% CI] | Sensitivity (Fraction) | Sensitivity [95% CI] | Specificity (Fraction) | Specificity [95% CI] | PPV (Fraction) | PPV [95% CI] | O/E (Fraction) | O/E [95% CI] | AUROC | AUROC [95% CI] | Accuracy (Fraction) | Accuracy [95% CI] |
|---|---|---|---|---|---|---|---|---|---|---|---|---|---|---|---|
| Overall | 114 | 0.69 (79/114) | [0.6, 0.78] | 0.89 (70/79) | [0.82, 0.96] | 0.57 (20/35) | [0.4, 0.74] | 0.82 (70/85) | [0.74, 0.91] | 1.7 (79/46.2) | [1.5, 1.9] | 0.85 | [0.77, 0.94] | 0.79 (90/114) | [0.72, 0.87] |
| Sex: Female | 48 | 0.67 (32/48) | [0.52, 0.8] | 0.91 (29/32) | [0.81, 1.01] | 0.5 (8/16) | [0.25, 0.75] | 0.78 (29/37) | [0.65, 0.91] | 1.6 (32/20.4) | [1.3, 1.9] | 0.77 | [0.63, 0.93] | 0.77 (37/48) | [0.67, 0.9] |
| Sex: Male | 66 | 0.71 (47/66) | [0.59, 0.82] | 0.87 (41/47) | [0.78, 0.97] | 0.63 (12/19) | [0.43, 0.86] | 0.85 (41/48) | [0.77, 0.96] | 1.8 (47/25.7) | [1.6, 2.1] | 0.92 | [0.86, 0.99] | 0.8 (53/66) | [0.71, 0.91] |

**Supplemental Table 9: Stanford HM ACP in Inpatient Oncology: Reliability and Fairness Audit by Sex.** Significant differences in prevalence, significantly lower performance, or significantly higher O/E are bolded.

| Group | Sample Size | Prevalence (Fraction) | Prevalence [95% CI] | Sensitivity (Fraction) | Sensitivity [95% CI] | Specificity (Fraction) | Specificity [95% CI] | PPV (Fraction) | PPV [95% CI] | O/E (Fraction) | O/E [95% CI] | AUROC | AUROC [95% CI] | Accuracy (Fraction) | Accuracy [95% CI] |
|---|---|---|---|---|---|---|---|---|---|---|---|---|---|---|---|
| Overall | 114 | 0.69 (79/114) | [0.6, 0.78] | 0.89 (70/79) | [0.82, 0.96] | 0.57 (20/35) | [0.4, 0.74] | 0.82 (70/85) | [0.74, 0.91] | 1.7 (79/46.2) | [1.5, 1.9] | 0.85 | [0.77, 0.94] | 0.79 (90/114) | [0.72, 0.87] |
| Age: (20, 30] | 11 | 0.27 (3/11) | [0.06, 0.61] | 1.0 (3/3) | [1.0, 1.0] | 0.88 (7/8) | [0.75, 1.15] | 0.75 (3/4) | [0.5, 1.5] | 1.1 (3/2.7) | [0.4, 2.9] | 1 | [1.0, 1.0] | 0.91 (10/11) | [0.82, 1.09] |
| Age: (30, 40] | 12 | 0.5 (6/12) | [0.21, 0.79] | 0.83 (5/6) | [0.67, 1.17] | 0.5 (3/6) | [0.0, 1.0] | 0.62 (5/8) | [0.25, 1.0] | 1.5 (6/4.0) | [0.8, 2.6] | 0.86 | [0.72, 1.13] | 0.67 (8/12) | [0.42, 0.92] |
| Age: (40, 50] | 11 | 0.55 (6/11) | [0.23, 0.83] | 0.83 (5/6) | [0.67, 1.17] | **0.2 (1/5)** | **[-0.2, 0.4]** | 0.56 (5/9) | [0.24, 0.89] | 1.5 (6/4.0) | [0.9, 2.6] | 0.77 | [0.53, 1.13] | 0.55 (6/11) | [0.27, 0.82] |

| | | | | | | | | | | | | | | | |
|---|---|---|---|---|---|---|---|---|---|---|---|---|---|---|---|
| Age: (50, 60] | 22 | 0.64 (14/22) | [0.41, 0.83] | 0.93 (13/14) | [0.86, 1.08] | 0.75 (6/8) | [0.5, 1.1] | 0.87 (13/15) | [0.73, 1.07] | 1.8 (14/7.8) | [1.3, 2.5] | 0.98 | [0.96, 1.04] | 0.86 (19/22) | [0.73, 1.05] |
| Age: (60, 70] | 25 | 0.92 (23/25) | [0.74, 0.99] | 0.96 (22/23) | [0.91, 1.05] | 0.5 (1/2) | [0.0, 1.0] | 0.96 (22/23) | [0.91, 1.05] | 1.8 (23/12.6) | [1.6, 2.1] | 0.76 | [0.52, 1.15] | 0.92 (23/25) | [0.84, 1.04] |
| Age: (70, 80] | 20 | 0.8 (16/20) | [0.56, 0.94] | 0.75 (12/16) | [0.56, 0.97] | 0.5 (2/4) | [0.0, 1.0] | 0.86 (12/14) | [0.71, 1.08] | 1.9 (16/8.3) | [1.5, 2.4] | 0.69 | [0.38, 1.1] | 0.7 (14/20) | [0.5, 0.9] |
| Age: (80, 90] | 12 | 0.83 (10/12) | [0.52, 0.98] | 0.9 (9/10) | [0.8, 1.1] | **0.0 (0/2)** | **[0.0, 0.0]** | 0.82 (9/11) | [0.64, 1.05] | 1.6 (10/6.2) | [1.3, 2.1] | 0.3 | [0.0, 0.6] | 0.75 (9/12) | [0.5, 1.0] |
| Age: (90, 100] | 1 | 1.0 (1/1) | [0.03, 1] | 1.0 (1/1) | [1.0, 1.0] | nan (0/0) | N/A | 1.0 (1/1) | [1.0, 1.0] | 1.6 (1/0.6) | [1.6, 1.6] | nan | N/A | 1.0 (1/1) | [1.0, 1.0] |

**Supplemental Table 10: Stanford HM ACP in Inpatient Oncology: Reliability and Fairness Audit by Age.** Significant differences in prevalence, significantly lower performance, or significantly higher O/E are bolded.

| Group | Sample Size | Prevalence (Fraction) | Prevalence [95% CI] | Sensitivity (Fraction) | Sensitivity [95% CI] | Specificity (Fraction) | Specificity [95% CI] | PPV (Fraction) | PPV [95% CI] | O/E (Fraction) | O/E [95% CI] | AUROC | AUROC [95% CI] | Accuracy (Fraction) | Accuracy [95% CI] |
|---|---|---|---|---|---|---|---|---|---|---|---|---|---|---|---|
| Overall | 114 | 0.69 (79/114) | [0.6, 0.78] | 0.89 (70/79) | [0.82, 0.96] | 0.57 (20/35) | [0.4, 0.74] | 0.82 (70/85) | [0.74, 0.91] | 1.7 (79/46.2) | [1.5, 1.9] | 0.85 | [0.77, 0.94] | 0.79 (90/114) | [0.72, 0.87] |
| Ethnicity: Not Hispanic or Latino, Race: White | 35 | 0.66 (23/35) | [0.48, 0.81] | 0.96 (22/23) | [0.91, 1.06] | 0.5 (6/12) | [0.22, 0.78] | 0.79 (22/28) | [0.64, 0.94] | 1.7 (23/13.7) | [1.3, 2.1] | 0.81 | [0.64, 1.01] | 0.8 (28/35) | [0.69, 0.94] |
| Ethnicity: Not Hispanic or Latino, Race: Asian | 31 | 0.81 (25/31) | [0.63, 0.93] | 0.92 (23/25) | [0.84, 1.04] | 0.67 (4/6) | [0.33, 1.08] | 0.92 (23/25) | [0.84, 1.04] | 1.7 (25/14.8) | [1.4, 2.0] | 0.92 | [0.84, 1.06] | 0.87 (27/31) | [0.77, 1.0] |
| Ethnicity: Hispanic or Latino, Race: Other | 26 | 0.73 (19/26) | [0.52, 0.88] | 0.84 (16/19) | [0.68, 1.03] | 0.71 (5/7) | [0.43, 1.1] | 0.89 (16/18) | [0.78, 1.04] | 1.8 (19/10.3) | [1.5, 2.3] | 0.74 | [0.51, 0.99] | 0.81 (21/26) | [0.65, 0.96] |
| Ethnicity: Not Hispanic or Latino, Race: Other | 13 | 0.62 (8/13) | [0.32, 0.86] | 0.88 (7/8) | [0.75, 1.15] | 0.6 (3/5) | [0.2, 1.06] | 0.78 (7/9) | [0.56, 1.06] | 1.8 (8/4.5) | [1.1, 2.7] | 0.9 | [0.8, 1.13] | 0.77 (10/13) | [0.54, 1.0] |

| | | | | | | | | | | | | | | | |
|---|---|---|---|---|---|---|---|---|---|---|---|---|---|---|---|
| Ethnicity: Hispanic or Latino, Race: White | 3 | 0.33 (1/3) | [0.01, 0.91] | 1.0 (1/1) | [1.0, 1.0] | **0.0 (0/2)** | **[0.0, 0.0]** | **0.33 (1/3)** | **[-0.33, 0.67]** | 0.8 (1/1.3) | [0.2, 3.9] | 1 | [1.0, 1.0] | 0.33 (1/3) | [-0.33, 0.67] |
| Ethnicity: Not Hispanic or Latino, Race: Black or African American | 3 | 0.33 (1/3) | [0.01, 0.91] | **0.0 (0/1)** | **[0.0, 0.0]** | 0.5 (1/2) | [0.0, 1.0] | **0.0 (0/1)** | **[0.0, 0.0]** | 1.8 (1/0.6) | [0.4, 8.8] | 0 | [0.0, 0.0] | 0.33 (1/3) | [-0.33, 0.67] |
| Ethnicity: Hispanic or Latino, Race: American Indian or Alaska Native | 1 | 0.0 (0/1) | [0, 0.98] | nan (0/0) | N/A | 1.0 (1/1) | [1.0, 1.0] | nan (0/0) | N/A | 0.0 (0/0.1) | N/A | nan | N/A | 1.0 (1/1) | [1.0, 1.0] |
| Ethnicity: Not Hispanic or Latino, Race: American Indian or Alaska Native | 1 | 1.0 (1/1) | [0.03, 1] | **0.0 (0/1)** | **[0.0, 0.0]** | nan (0/0) | N/A | nan (0/0) | N/A | **4.1 (1/0.2)** | **[4.1, 4.1]** | **nan** | **N/A** | **0.0 (0/1)** | **[0.0, 0.0]** |

**Supplemental Table 11: Stanford HM ACP in Inpatient Oncology: Reliability and Fairness Audit by Ethnicity/Race.** Significant differences in prevalence, significantly lower performance, or significantly higher O/E are bolded.

| Group | Sample Size | Prevalence (Fraction) | Prevalence [95% CI] | Sensitivity (Fraction) | Sensitivity [95% CI] | Specificity (Fraction) | Specificity [95% CI] | PPV (Fraction) | PPV [95% CI] | O/E (Fraction) | O/E [95% CI] | AUROC | AUROC [95% CI] | Accuracy (Fraction) | Accuracy [95% CI] |
|---|---|---|---|---|---|---|---|---|---|---|---|---|---|---|---|
| Overall | 114 | 0.69 (79/114) | [0.6, 0.78] | 0.89 (70/79) | [0.82, 0.96] | 0.57 (20/35) | [0.4, 0.74] | 0.82 (70/85) | [0.74, 0.91] | 1.7 (79/46.2) | [1.5, 1.9] | 0.85 | [0.77, 0.94] | 0.79 (90/114) | [0.72, 0.87] |
| Ethnicity: Not Hispanic or Latino, Race: Asian, Sex: Male | 19 | 0.84 (16/19) | [0.6, 0.97] | 0.94 (15/16) | [0.88, 1.08] | 0.67 (2/3) | [0.33, 1.33] | 0.94 (15/16) | [0.88, 1.08] | 1.9 (16/8.4) | [1.6, 2.3] | 0.98 | [0.96, 1.07] | 0.89 (17/19) | [0.79, 1.05] |
| Ethnicity: Not Hispanic or Latino, Race: White, Sex: Male | 18 | 0.78 (14/18) | [0.52, 0.94] | 0.93 (13/14) | [0.86, 1.09] | 0.75 (3/4) | [0.5, 1.3] | 0.93 (13/14) | [0.86, 1.07] | 1.9 (14/7.4) | [1.5, 2.4] | 0.95 | [0.89, 1.12] | 0.89 (16/18) | [0.78, 1.06] |
| Ethnicity: Not Hispanic or Latino, Race: White, Sex: Female | 17 | 0.53 (9/17) | [0.28, 0.77] | 1.0 (9/9) | [1.0, 1.0] | 0.38 (3/8) | [-0.03, 0.75] | 0.64 (9/14) | [0.38, 0.9] | 1.4 (9/6.3) | [0.9, 2.2] | 0.75 | [0.5, 1.07] | 0.71 (12/17) | [0.47, 0.94] |

| | | | | | | | | | | | | | | | |
|---|---|---|---|---|---|---|---|---|---|---|---|---|---|---|---|
| Ethnicity: Hispanic or Latino, Race: Other, Sex: Male | 15 | 0.8 (12/15) | [0.52, 0.96] | 0.83 (10/12) | [0.67, 1.07] | 0.67 (2/3) | [0.33, 1.33] | 0.91 (10/11) | [0.82, 1.12] | 1.9 (12/6.2) | [1.5, 2.5] | 0.78 | [0.56, 1.17] | 0.8 (12/15) | [0.6, 1.0] |
| Ethnicity: Not Hispanic or Latino, Race: Asian, Sex: Female | 12 | 0.75 (9/12) | [0.43, 0.95] | 0.89 (8/9) | [0.78, 1.11] | 0.67 (2/3) | [0.33, 1.33] | 0.89 (8/9) | [0.78, 1.11] | 1.4 (9/6.4) | [1.0, 1.9] | 0.85 | [0.7, 1.16] | 0.83 (10/12) | [0.67, 1.08] |
| Ethnicity: Hispanic or Latino, Race: Other, Sex: Female | 11 | 0.64 (7/11) | [0.31, 0.89] | 0.86 (6/7) | [0.71, 1.16] | 0.75 (3/4) | [0.5, 1.25] | 0.86 (6/7) | [0.71, 1.21] | 1.7 (7/4.2) | [1.1, 2.6] | 0.75 | [0.5, 1.17] | 0.82 (9/11) | [0.64, 1.09] |
| Ethnicity: Not Hispanic or Latino, Race: Other, Sex: Male | 8 | 0.38 (3/8) | [0.09, 0.76] | 0.67 (2/3) | [0.33, 1.33] | 0.6 (3/5) | [0.2, 1.2] | 0.5 (2/4) | [0.0, 1.0] | 1.4 (3/2.1) | [0.6, 3.5] | 0.87 | [0.73, 1.27] | 0.62 (5/8) | [0.38, 1.0] |
| Ethnicity: Not Hispanic or Latino, Race: Other, Sex: Female | 5 | 1.0 (5/5) | [0.48, 1] | 1.0 (1/1) | [1.0, 1.0] | nan (0/0) | N/A | 1.0 (1/1) | [1.0, 1.0] | **2.0 (5/2.4)** | **[2.0, 2.0]** | **nan** | **N/A** | **1.0 (5/5)** | **[1.0, 1.0]** |
| Ethnicity: Hispanic or Latino, Race: White, Sex: Male | 2 | 0.0 (0/2) | [0, 0.84] | nan (0/0) | N/A | **0.0 (0/2)** | **[0.0, 0.0]** | **0.0 (0/2)** | **[0.0, 0.0]** | 0.0 (0/0.5) | N/A | nan | N/A | 0.0 (0/2) | [0.0, 0.0] |
| Ethnicity: Not Hispanic or Latino, Race: Black or African American, Sex: Female | 2 | 0.5 (1/2) | [0.01, 0.99] | **0.0 (0/1)** | **[0.0, 0.0]** | **0.0 (0/1)** | **[0.0, 0.0]** | **0.0 (0/1)** | **[0.0, 0.0]** | 2.6 (1/0.4) | [0.7, 10.6] | 0 | [0.0, 0.0] | 0.0 (0/2) | [0.0, 0.0] |
| Ethnicity: Hispanic or Latino, Race: White, Sex: Female | 1 | 1.0 (1/1) | [0.03, 1] | 1.0 (1/1) | [1.0, 1.0] | nan (0/0) | N/A | 1.0 (1/1) | [1.0, 1.0] | 1.4 (1/0.7) | [1.4, 1.4] | nan | N/A | 1.0 (1/1) | [1.0, 1.0] |
| Ethnicity: Not Hispanic or Latino, Race: Black or African American, Sex: Male | 1 | 0.0 (0/1) | [0, 0.98] | nan (0/0) | N/A | 1.0 (1/1) | [1.0, 1.0] | nan (0/0) | N/A | 0.0 (0/0.2) | N/A | nan | N/A | 1.0 (1/1) | [1.0, 1.0] |
| Ethnicity: Hispanic or Latino, Race: American Indian or | 1 | 0.0 (0/1) | [0, 0.98] | nan (0/0) | N/A | 1.0 (1/1) | [1.0, 1.0] | nan (0/0) | N/A | 0.0 (0/0.1) | N/A | nan | N/A | 1.0 (1/1) | [1.0, 1.0] |

| | | | | | | | | | | | | | | | |
|---|---|---|---|---|---|---|---|---|---|---|---|---|---|---|---|
| Alaska Native, Sex: Male | | | | | | | | | | | | | | | |
| Ethnicity: Not Hispanic or Latino, Race: American Indian or Alaska Native, Sex: Male | 1 | 1.0 (1/1) | [0.03, 1] | **0.0 (0/1)** | **[0.0, 0.0]** | nan (0/0) | N/A | nan (0/0) | N/A | **4.1 (1/0.2)** | **[4.1, 4.1]** | **nan** | **N/A** | **0.0 (0/1)** | **[0.0, 0.0]** |

**Supplemental Table 12: Stanford HM ACP in Inpatient Oncology: Reliability and Fairness Audit by Ethnicity/Race and Sex.** Significant differences in prevalence, significantly lower performance, or significantly higher O/E are bolded.

## *Epic EOL High Threshold in Hospital Medicine*

| Group | Sample Size | Prevalence (Fraction) | Prevalence [95% CI] | Sensitivity (Fraction) | Sensitivity [95% CI] | Specificity (Fraction) | Specificity [95% CI] | PPV (Fraction) | PPV [95% CI] | O/E (Fraction) | O/E [95% CI] | AUROC | AUROC [95% CI] | Accuracy (Fraction) | Accuracy [95% CI] |
|---|---|---|---|---|---|---|---|---|---|---|---|---|---|---|---|
| Overall | 305 | 0.44 (133/305) | [0.38, 0.49] | 0.2 (26/133) | [0.12, 0.26] | 0.95 (164/172) | [0.92, 0.99] | 0.76 (26/34) | [0.63, 0.91] | 2.5 (133/53.2) | [2.2, 2.8] | 0.78 | [0.73, 0.83] | 0.62 (190/305) | [0.57, 0.67] |
| Sex: Female | 140 | 0.47 (66/140) | [0.39, 0.56] | 0.26 (17/66) | [0.14, 0.36] | 0.95 (70/74) | [0.9, 1.0] | 0.81 (17/21) | [0.67, 0.99] | 2.6 (66/25.2) | [2.2, 3.1] | 0.82 | [0.76, 0.89] | 0.62 (87/140) | [0.55, 0.71] |
| Sex: Male | 165 | 0.41 (67/165) | [0.33, 0.49] | 0.13 (9/67) | [0.05, 0.21] | 0.96 (94/98) | [0.93, 1.01] | 0.69 (9/13) | [0.45, 0.97] | 2.4 (67/28.0) | [2.0, 2.9] | 0.75 | [0.68, 0.83] | 0.62 (103/165) | [0.55, 0.7] |

**Supplemental Table 13: Epic EOL High Threshold in Hospital Medicine: Reliability and Fairness Audit by Sex.** Significant differences in prevalence, significantly lower performance, or significantly higher O/E are bolded.

| Group | Sample Size | Prevalence (Fraction) | Prevalence [95% CI] | Sensitivity (Fraction) | Sensitivity [95% CI] | Specificity (Fraction) | Specificity [95% CI] | PPV (Fraction) | PPV [95% CI] | O/E (Fraction) | O/E [95% CI] | AUROC | AUROC [95% CI] | Accuracy (Fraction) | Accuracy [95% CI] |
|---|---|---|---|---|---|---|---|---|---|---|---|---|---|---|---|

| | | | | | | | | | | | | | | | |
|---|---|---|---|---|---|---|---|---|---|---|---|---|---|---|---|
| Overall | 305 | 0.44 (133/305) | [0.38, 0.49] | 0.2 (26/133) | [0.12, 0.26] | 0.95 (164/172) | [0.92, 0.99] | 0.76 (26/34) | [0.63, 0.91] | 2.5 (133/53.2) | [2.2, 2.8] | 0.78 | [0.73, 0.83] | 0.62 (190/305) | [0.57, 0.67] |
| Age: (10, 20] | 3 | 0.33 (1/3) | [0.01, 0.91] | **0.0 (0/1)** | **[0.0, 0.0]** | 1.0 (2/2) | [1.0, 1.0] | nan (0/0) | N/A | **inf (1/0.0)** | **[inf, inf]** | **0.5** | **[0.5, 0.5]** | **0.67 (2/3)** | **[0.33, 1.33]** |
| Age: (20, 30] | 24 | **0.12 (3/24)** | **[0.03, 0.32]** | **0.0 (0/3)** | **[0.0, 0.0]** | 1.0 (21/21) | [1.0, 1.0] | nan (0/0) | N/A | 4.5 (3/0.7) | [1.6, 12.9] | 0.98 | [0.97, 1.04] | 0.88 (21/24) | [0.75, 1.04] |
| Age: (30, 40] | 40 | **0.15 (6/40)** | **[0.06, 0.3]** | **0.0 (0/6)** | **[0.0, 0.0]** | 1.0 (34/34) | [1.0, 1.0] | nan (0/0) | N/A | 4.7 (6/1.3) | [2.2, 9.7] | 0.49 | [0.31, 0.65] | 0.85 (34/40) | [0.75, 0.98] |
| Age: (40, 50] | 12 | 0.5 (6/12) | [0.21, 0.79] | 0.17 (1/6) | [-0.17, 0.33] | 1.0 (6/6) | [1.0, 1.0] | 1.0 (1/1) | [1.0, 1.0] | 3.6 (6/1.7) | [2.0, 6.3] | 0.65 | [0.36, 1.05] | 0.58 (7/12) | [0.33, 0.84] |
| Age: (50, 60] | 40 | 0.28 (11/40) | [0.15, 0.44] | **0.0 (0/11)** | **[0.0, 0.0]** | 1.0 (29/29) | [1.0, 1.0] | nan (0/0) | N/A | 3.6 (11/3.1) | [2.2, 5.9] | 0.7 | [0.52, 0.91] | 0.72 (29/40) | [0.6, 0.88] |
| Age: (60, 70] | 72 | 0.44 (32/72) | [0.33, 0.57] | 0.19 (6/32) | [0.04, 0.31] | 0.92 (37/40) | [0.85, 1.01] | 0.67 (6/9) | [0.33, 1.0] | 2.2 (32/14.7) | [1.7, 2.8] | 0.77 | [0.66, 0.89] | 0.6 (43/72) | [0.49, 0.71] |
| Age: (70, 80] | 62 | 0.5 (31/62) | [0.37, 0.63] | 0.32 (10/31) | [0.16, 0.48] | 0.9 (28/31) | [0.81, 1.02] | 0.77 (10/13) | [0.54, 1.04] | 2.2 (31/14.2) | [1.7, 2.8] | 0.61 | [0.46, 0.76] | 0.61 (38/62) | [0.5, 0.74] |
| Age: (80, 90] | 34 | **0.76 (26/34)** | **[0.59, 0.89]** | 0.19 (5/26) | [0.02, 0.34] | 0.88 (7/8) | [0.75, 1.18] | 0.83 (5/6) | [0.67, 1.17] | 2.6 (26/10.0) | [2.2, 3.1] | 0.5 | [0.25, 0.75] | 0.35 (12/34) | [0.18, 0.5] |
| Age: (90, 100] | 18 | **0.94 (17/18)** | **[0.73, 1.0]** | 0.24 (4/17) | [0.03, 0.41] | **0.0 (0/1)** | **[0.0, 0.0]** | 0.8 (4/5) | [0.6, 1.27] | 2.2 (17/7.6) | [2.0, 2.5] | 0 | [0.0, 0.0] | 0.22 (4/18) | [0.0, 0.39] |

**Supplemental Table 14: Epic EOL High Threshold in Hospital Medicine: Reliability and Fairness Audit by Age.** Significant differences in prevalence, significantly lower performance, or significantly higher O/E are bolded.

| Group | Sample Size | Prevalence (Fraction) | Prevalence [95% CI] | Sensitivity (Fraction) | Sensitivity [95% CI] | Specificity (Fraction) | Specificity [95% CI] | PPV (Fraction) | PPV [95% CI] | O/E (Fraction) | O/E [95% CI] | AUROC | AUROC [95% CI] | Accuracy (Fraction) | Accuracy [95% CI] |
|---|---|---|---|---|---|---|---|---|---|---|---|---|---|---|---|
| Overall | 305 | 0.44 (133/305) | [0.38, 0.49] | 0.2 (26/133) | [0.12, 0.26] | 0.95 (164/172) | [0.92, 0.99] | 0.76 (26/34) | [0.63, 0.91] | 2.5 (133/53.2) | [2.2, 2.8] | 0.78 | [0.73, 0.83] | 0.62 (190/305) | [0.57, 0.67] |
| Ethnicity: Not Hispanic or Latino, Race: White | 145 | 0.44 (64/145) | [0.36, 0.53] | 0.09 (6/64) | [0.01, 0.16] | 0.95 (77/81) | [0.91, 1.0] | 0.6 (6/10) | [0.27, 0.95] | 3.1 (64/20.4) | [2.6, 3.8] | 0.76 | [0.68, 0.84] | 0.57 (83/145) | [0.48, 0.66] |

| | | | | | | | | | | | | | | | |
|---|---|---|---|---|---|---|---|---|---|---|---|---|---|---|---|
| Ethnicity: Hispanic or Latino, Race: Other | 44 | **0.18 (8/44)** | **[0.08, 0.33]** | 0.12 (1/8) | [-0.17, 0.25] | 0.94 (34/36) | [0.89, 1.02] | 0.33 (1/3) | [-0.33, 0.67] | 2.0 (8/4.0) | [1.1, 3.7] | 0.67 | [0.47, 0.87] | 0.8 (35/44) | [0.68, 0.91] |
| Ethnicity: Not Hispanic or Latino, Race: Asian | 37 | **0.68 (25/37)** | **[0.5, 0.82]** | 0.32 (8/25) | [0.12, 0.52] | 1.0 (12/12) | [1.0, 1.0] | 1.0 (8/8) | [1.0, 1.0] | 2.1 (25/12.2) | [1.6, 2.6] | 0.91 | [0.84, 1.03] | 0.54 (20/37) | [0.38, 0.73] |
| Ethnicity: Not Hispanic or Latino, Race: Black or African American | 35 | 0.54 (19/35) | [0.37, 0.71] | 0.47 (9/19) | [0.26, 0.7] | 0.88 (14/16) | [0.75, 1.06] | 0.82 (9/11) | [0.64, 1.08] | 1.6 (19/12.2) | [1.2, 2.1] | 0.77 | [0.63, 0.94] | 0.66 (23/35) | [0.51, 0.83] |
| Ethnicity: Not Hispanic or Latino, Race: Other | 16 | 0.5 (8/16) | [0.25, 0.75] | 0.25 (2/8) | [-0.1, 0.5] | 1.0 (8/8) | [1.0, 1.0] | 1.0 (2/2) | [1.0, 1.0] | 4.0 (8/2.0) | [2.4, 6.5] | 0.57 | [0.29, 0.91] | 0.62 (10/16) | [0.38, 0.88] |
| Ethnicity: Hispanic or Latino, Race: White | 13 | 0.23 (3/13) | [0.05, 0.54] | **0.0 (0/3)** | **[0.0, 0.0]** | 1.0 (10/10) | [1.0, 1.0] | nan (0/0) | N/A | 4.4 (3/0.7) | [1.6, 11.9] | 0.92 | [0.83, 1.12] | 0.77 (10/13) | [0.54, 1.0] |
| Ethnicity: Not Hispanic or Latino, Race: Native Hawaiian or Other Pacific Islander | 10 | 0.4 (4/10) | [0.12, 0.74] | **0.0 (0/4)** | **[0.0, 0.0]** | 1.0 (6/6) | [1.0, 1.0] | nan (0/0) | N/A | 3.0 (4/1.3) | [1.4, 6.4] | 0.83 | [0.67, 1.17] | 0.6 (6/10) | [0.3, 0.9] |
| Ethnicity: Not Hispanic or Latino, Race: American Indian or Alaska Native | 1 | 0.0 (0/1) | [0, 0.98] | nan (0/0) | N/A | 1.0 (1/1) | [1.0, 1.0] | nan (0/0) | N/A | 0.0 (0/0.2) | N/A | nan | N/A | 1.0 (1/1) | [1.0, 1.0] |

**Supplemental Table 15: Epic EOL High Threshold in Hospital Medicine: Reliability and Fairness Audit by Ethnicity/Race.** Significant differences in prevalence, significantly lower performance, or significantly higher O/E are bolded.

| Group | Sample Size | Prevalence (Fraction) | Prevalence [95% CI] | Sensitivity (Fraction) | Sensitivity [95% CI] | Specificity (Fraction) | Specificity [95% CI] | PPV (Fraction) | PPV [95% CI] | O/E (Fraction) | O/E [95% CI] | AUROC | AUROC [95% CI] | Accuracy (Fraction) | Accuracy [95% CI] |
|---|---|---|---|---|---|---|---|---|---|---|---|---|---|---|---|
| Overall | 305 | 0.44 (133/305) | [0.38, 0.49] | 0.2 (26/133) | [0.12, 0.26] | 0.95 (164/172) | [0.92, 0.99] | 0.76 (26/34) | [0.63, 0.91] | 2.5 (133/53.2) | [2.2, 2.8] | 0.78 | [0.73, 0.83] | 0.62 (190/305) | [0.57, 0.67] |

| | | | | | | | | | | | | | | | |
|---|---|---|---|---|---|---|---|---|---|---|---|---|---|---|---|
| Ethnicity: Not Hispanic or Latino, Race: White, Sex: Male | 81 | 0.38 (31/81) | [0.28, 0.5] | 0.06 (2/31) | [-0.04, 0.13] | 0.94 (47/50) | [0.88, 1.01] | 0.4 (2/5) | [-0.2, 0.8] | 2.7 (31/11.4) | [2.1, 3.6] | 0.71 | [0.59, 0.84] | 0.6 (49/81) | [0.51, 0.72] |
| Ethnicity: Not Hispanic or Latino, Race: White, Sex: Female | 64 | 0.52 (33/64) | [0.39, 0.64] | 0.12 (4/33) | [0.01, 0.22] | 0.97 (30/31) | [0.94, 1.04] | 0.8 (4/5) | [0.6, 1.27] | **3.7 (33/9.0)** | **[2.9, 4.6]** | **0.85** | **[0.76, 0.95]** | **0.53 (34/64)** | **[0.41, 0.66]** |
| Ethnicity: Hispanic or Latino, Race: Other, Sex: Female | 22 | 0.23 (5/22) | [0.08, 0.45] | 0.2 (1/5) | [-0.27, 0.4] | 0.88 (15/17) | [0.76, 1.05] | 0.33 (1/3) | [-0.33, 0.67] | 1.5 (5/3.4) | [0.7, 3.2] | 0.68 | [0.45, 0.94] | 0.73 (16/22) | [0.55, 0.91] |
| Ethnicity: Hispanic or Latino, Race: Other, Sex: Male | 22 | **0.14 (3/22)** | **[0.03, 0.35]** | **0.0 (0/3)** | **[0.0, 0.0]** | 1.0 (19/19) | [1.0, 1.0] | nan (0/0) | N/A | 4.3 (3/0.7) | [1.5, 12.4] | 0.58 | [0.3, 0.85] | 0.86 (19/22) | [0.73, 1.0] |
| Ethnicity: Not Hispanic or Latino, Race: Asian, Sex: Male | 21 | 0.71 (15/21) | [0.48, 0.89] | 0.13 (2/15) | [-0.07, 0.27] | 1.0 (6/6) | [1.0, 1.0] | 1.0 (2/2) | [1.0, 1.0] | 2.1 (15/7.3) | [1.6, 2.7] | 0.84 | [0.68, 1.06] | 0.38 (8/21) | [0.14, 0.57] |
| Ethnicity: Not Hispanic or Latino, Race: Black or African American, Sex: Male | 20 | 0.45 (9/20) | [0.23, 0.68] | 0.56 (5/9) | [0.24, 0.91] | 0.91 (10/11) | [0.82, 1.15] | 0.83 (5/6) | [0.67, 1.22] | 1.3 (9/6.8) | [0.8, 2.2] | 0.82 | [0.64, 1.05] | 0.75 (15/20) | [0.6, 0.95] |
| Ethnicity: Not Hispanic or Latino, Race: Asian, Sex: Female | 16 | 0.62 (10/16) | [0.35, 0.85] | 0.6 (6/10) | [0.3, 0.91] | 1.0 (6/6) | [1.0, 1.0] | 1.0 (6/6) | [1.0, 1.0] | 2.0 (10/4.9) | [1.4, 3.0] | 1 | [1.0, 1.0] | 0.75 (12/16) | [0.56, 1.0] |
| Ethnicity: Not Hispanic or Latino, Race: Black or African American, Sex: | 15 | 0.67 (10/15) | [0.38, 0.88] | 0.4 (4/10) | [0.09, 0.7] | 0.8 (4/5) | [0.6, 1.27] | 0.8 (4/5) | [0.6, 1.27] | 1.9 (10/5.4) | [1.3, 2.7] | 0.78 | [0.56, 1.12] | 0.53 (8/15) | [0.27, 0.8] |

| | | | | | | | | | | | | | | | |
|---|---|---|---|---|---|---|---|---|---|---|---|---|---|---|---|
| Female | | | | | | | | | | | | | | | |
| Ethnicity: Not Hispanic or Latino, Race: Other, Sex: Male | 9 | 0.56 (5/9) | [0.21, 0.86] | **0.0 (0/5)** | **[0.0, 0.0]** | 1.0 (4/4) | [1.0, 1.0] | nan (0/0) | N/A | **13.9 (5/0.4)** | **[7.7, 24.9]** | **0.12** | **[-0.25, 0.25]** | **0.44 (4/9)** | **[0.11, 0.78]** |
| Ethnicity: Not Hispanic or Latino, Race: Other, Sex: Female | 7 | 0.43 (3/7) | [0.1, 0.82] | 0.67 (2/3) | [0.33, 1.33] | 1.0 (4/4) | [1.0, 1.0] | 1.0 (2/2) | [1.0, 1.0] | 1.8 (3/1.7) | [0.8, 4.3] | 1 | [1.0, 1.0] | 0.86 (6/7) | [0.71, 1.14] |
| Ethnicity: Hispanic or Latino, Race: White, Sex: Male | 7 | 0.14 (1/7) | [0.0, 0.58] | **0.0 (0/1)** | **[0.0, 0.0]** | 1.0 (6/6) | [1.0, 1.0] | nan (0/0) | N/A | 10.0 (1/0.1) | [1.6, 61.4] | 1 | [1.0, 1.0] | 0.86 (6/7) | [0.71, 1.14] |
| Ethnicity: Hispanic or Latino, Race: White, Sex: Female | 6 | 0.33 (2/6) | [0.04, 0.78] | **0.0 (0/2)** | **[0.0, 0.0]** | 1.0 (4/4) | [1.0, 1.0] | nan (0/0) | N/A | 3.4 (2/0.6) | [1.1, 10.7] | 0.94 | [0.88, 1.18] | 0.67 (4/6) | [0.33, 1.0] |
| Ethnicity: Not Hispanic or Latino, Race: Native Hawaiian or Other Pacific Islander, Sex: Female | 6 | 0.17 (1/6) | [0.0, 0.64] | **0.0 (0/1)** | **[0.0, 0.0]** | 1.0 (5/5) | [1.0, 1.0] | nan (0/0) | N/A | 7.7 (1/0.1) | [1.3, 46.0] | 0.4 | [0.3, 0.6] | 0.83 (5/6) | [0.67, 1.17] |
| Ethnicity: Not Hispanic or Latino, Race: Native Hawaiian or Other Pacific Islander, Sex: Male | 4 | 0.75 (3/4) | [0.19, 0.99] | **0.0 (0/3)** | **[0.0, 0.0]** | 1.0 (1/1) | [1.0, 1.0] | nan (0/0) | N/A | 2.5 (3/1.2) | [1.4, 4.4] | 1 | [1.0, 1.0] | 0.25 (1/4) | [-0.25, 0.5] |
| Ethnicity: Not Hispanic or Latino, Race: American Indian | 1 | 0.0 (0/1) | [0, 0.98] | nan (0/0) | N/A | 1.0 (1/1) | [1.0, 1.0] | nan (0/0) | N/A | 0.0 (0/0.2) | N/A | nan | N/A | 1.0 (1/1) | [1.0, 1.0] |

| | | | | | | | | | | | | | | | |
|---|---|---|---|---|---|---|---|---|---|---|---|---|---|---|---|
| or Alaska Native, Sex: Male | | | | | | | | | | | | | | | |

**Supplemental Table 16: Epic EOL High Threshold in Hospital Medicine: Reliability and Fairness Audit by Ethnicity/Race and Sex.** Significant differences in prevalence, significantly lower performance, or significantly higher O/E are bolded.

## *Stanford HM ACP in Hospital Medicine*

| Group | Sample Size | Prevalence (Fraction) | Prevalence [95% CI] | Sensitivity (Fraction) | Sensitivity [95% CI] | Specificity (Fraction) | Specificity [95% CI] | PPV (Fraction) | PPV [95% CI] | O/E (Fraction) | O/E [95% CI] | AUROC | AUROC [95% CI] | Accuracy (Fraction) | Accuracy [95% CI] |
|---|---|---|---|---|---|---|---|---|---|---|---|---|---|---|---|
| Overall | 225 | 0.44 (99/225) | [0.37, 0.51] | 0.69 (68/99) | [0.6, 0.78] | 0.87 (109/126) | [0.81, 0.93] | 0.8 (68/85) | [0.72, 0.89] | 1.5 (99/65.2) | [1.3, 1.8] | 0.86 | [0.81, 0.91] | 0.79 (177/225) | [0.73, 0.84] |
| Sex: Female | 114 | 0.45 (51/114) | [0.35, 0.54] | 0.73 (37/51) | [0.61, 0.86] | 0.83 (52/63) | [0.73, 0.92] | 0.77 (37/48) | [0.66, 0.9] | 1.4 (51/35.7) | [1.2, 1.7] | 0.86 | [0.8, 0.93] | 0.78 (89/114) | [0.71, 0.86] |
| Sex: Male | 111 | 0.43 (48/111) | [0.34, 0.53] | 0.65 (31/48) | [0.51, 0.78] | 0.9 (57/63) | [0.84, 0.98] | 0.84 (31/37) | [0.73, 0.96] | 1.6 (48/29.5) | [1.3, 2.0] | 0.87 | [0.8, 0.94] | 0.79 (88/111) | [0.72, 0.87] |

**Supplemental Table 17: Stanford HM ACP in Hospital Medicine: Reliability and Fairness Audit by Sex.** Significant differences in prevalence, significantly lower performance, or significantly higher O/E are bolded.

| Group | Sample Size | Prevalence (Fraction) | Prevalence [95% CI] | Sensitivity (Fraction) | Sensitivity [95% CI] | Specificity (Fraction) | Specificity [95% CI] | PPV (Fraction) | PPV [95% CI] | O/E (Fraction) | O/E [95% CI] | AUROC | AUROC [95% CI] | Accuracy (Fraction) | Accuracy [95% CI] |
|---|---|---|---|---|---|---|---|---|---|---|---|---|---|---|---|
| Overall | 225 | 0.44 (99/225) | [0.37, 0.51] | 0.69 (68/99) | [0.6, 0.78] | 0.87 (109/126) | [0.81, 0.93] | 0.8 (68/85) | [0.72, 0.89] | 1.5 (99/65.2) | [1.3, 1.8] | 0.86 | [0.81, 0.91] | 0.79 (177/225) | [0.73, 0.84] |
| Age: (10, 20] | 3 | 0.33 (1/3) | [0.01, 0.91] | **0.0 (0/1)** | **[0.0, 0.0]** | 1.0 (2/2) | [1.0, 1.0] | nan (0/0) | N/A | 2.4 (1/0.4) | [0.5, 12.1] | 0.5 | [0.5, 0.5] | 0.67 (2/3) | [0.33, 1.33] |
| Age: (20, 30] | 13 | 0.15 (2/13) | [0.02, 0.45] | 0.5 (1/2) | [0.0, 1.0] | 1.0 (11/11) | [1.0, 1.0] | 1.0 (1/1) | [1.0, 1.0] | 1.0 (2/2.0) | [0.3, 3.7] | 0.77 | [0.55, 1.18] | 0.92 (12/13) | [0.85, 1.08] |

| | | | | | | | | | | | | | | | |
|---|---|---|---|---|---|---|---|---|---|---|---|---|---|---|---|
| Age: (30, 40] | 28 | **0.11 (3/28)** | **[0.02, 0.28]** | **0.0 (0/3)** | **[0.0, 0.0]** | 1.0 (25/25) | [1.0, 1.0] | nan (0/0) | N/A | 0.8 (3/3.7) | [0.3, 2.3] | 0.67 | [0.35, 1.18] | 0.89 (25/28) | [0.82, 1.0] |
| Age: (40, 50] | 15 | 0.4 (6/15) | [0.16, 0.68] | 0.33 (2/6) | [-0.08, 0.67] | 1.0 (9/9) | [1.0, 1.0] | 1.0 (2/2) | [1.0, 1.0] | 1.8 (6/3.4) | [1.0, 3.3] | 0.76 | [0.52, 1.07] | 0.73 (11/15) | [0.53, 0.93] |
| Age: (50, 60] | 25 | 0.24 (6/25) | [0.09, 0.45] | **0.17 (1/6)** | **[-0.17, 0.33]** | 1.0 (19/19) | [1.0, 1.0] | 1.0 (1/1) | [1.0, 1.0] | 1.5 (6/4.1) | [0.7, 2.9] | 0.75 | [0.53, 1.06] | 0.8 (20/25) | [0.64, 0.96] |
| Age: (60, 70] | 50 | 0.36 (18/50) | [0.23, 0.51] | 0.78 (14/18) | [0.6, 0.98] | 0.84 (27/32) | [0.73, 0.98] | 0.74 (14/19) | [0.56, 0.95] | 1.2 (18/14.9) | [0.8, 1.8] | 0.86 | [0.77, 0.98] | 0.82 (41/50) | [0.72, 0.94] |
| Age: (70, 80] | 48 | 0.56 (27/48) | [0.41, 0.71] | 0.81 (22/27) | [0.67, 0.99] | **0.57 (12/21)** | **[0.37, 0.78]** | 0.71 (22/31) | [0.56, 0.88] | 1.3 (27/20.2) | [1.0, 1.7] | 0.86 | [0.77, 0.98] | 0.71 (34/48) | [0.58, 0.85] |
| Age: (80, 90] | 30 | **0.8 (24/30)** | **[0.61, 0.92]** | 0.75 (18/24) | [0.59, 0.93] | 0.5 (3/6) | [0.0, 1.0] | 0.86 (18/21) | [0.71, 1.01] | 2.1 (24/11.7) | [1.7, 2.5] | 0.77 | [0.6, 0.96] | 0.7 (21/30) | [0.53, 0.87] |
| Age: (90, 100] | 13 | **0.92 (12/13)** | **[0.64, 1.0]** | 0.83 (10/12) | [0.67, 1.05] | 1.0 (1/1) | [1.0, 1.0] | 1.0 (10/10) | [1.0, 1.0] | **2.5 (12/4.9)** | **[2.1, 2.9]** | **0.83** | **[0.67, 1.07]** | **0.85 (11/13)** | **[0.69, 1.08]** |

**Supplemental Table 18: Stanford HM ACP in Hospital Medicine: Reliability and Fairness Audit by Age.** Significant differences in prevalence, significantly lower performance, or significantly higher O/E are bolded.

| Group | Sample Size | Prevalence (Fraction) | Prevalence [95% CI] | Sensitivity (Fraction) | Sensitivity [95% CI] | Specificity (Fraction) | Specificity [95% CI] | PPV (Fraction) | PPV [95% CI] | O/E (Fraction) | O/E [95% CI] | AUROC | AUROC [95% CI] | Accuracy (Fraction) | Accuracy [95% CI] |
|---|---|---|---|---|---|---|---|---|---|---|---|---|---|---|---|
| Overall | 225 | 0.44 (99/225) | [0.37, 0.51] | 0.69 (68/99) | [0.6, 0.78] | 0.87 (109/126) | [0.81, 0.93] | 0.8 (68/85) | [0.72, 0.89] | 1.5 (99/65.2) | [1.3, 1.8] | 0.86 | [0.81, 0.91] | 0.79 (177/225) | [0.73, 0.84] |
| Ethnicity: Not Hispanic or Latino, Race: White | 81 | 0.44 (36/81) | [0.33, 0.56] | 0.67 (24/36) | [0.51, 0.81] | 0.91 (41/45) | [0.84, 1.0] | 0.86 (24/28) | [0.74, 1.0] | 1.8 (36/20.2) | [1.4, 2.3] | 0.88 | [0.81, 0.95] | 0.8 (65/81) | [0.72, 0.89] |
| Ethnicity: Hispanic or Latino, Race: Other | 38 | **0.16 (6/38)** | **[0.06, 0.31]** | 0.33 (2/6) | [-0.13, 0.67] | 0.84 (27/32) | [0.72, 0.96] | **0.29 (2/7)** | **[-0.1, 0.57]** | 0.9 (6/7.0) | [0.4, 1.8] | 0.66 | [0.39, 0.94] | 0.76 (29/38) | [0.63, 0.89] |

| | | | | | | | | | | | | | | | |
|---|---|---|---|---|---|---|---|---|---|---|---|---|---|---|---|
| Ethnicity: Not Hispanic or Latino, Race: Asian | 37 | **0.7 (26/37)** | **[0.53, 0.84]** | 0.73 (19/26) | [0.58, 0.91] | 0.91 (10/11) | [0.82, 1.13] | 0.95 (19/20) | [0.9, 1.07] | 1.6 (26/16.1) | [1.3, 2.0] | 0.94 | [0.88, 1.05] | 0.78 (29/37) | [0.68, 0.92] |
| Ethnicity: Not Hispanic or Latino, Race: Black or African American | 21 | 0.43 (9/21) | [0.22, 0.66] | 0.67 (6/9) | [0.33, 1.03] | 0.83 (10/12) | [0.67, 1.05] | 0.75 (6/8) | [0.5, 1.1] | 1.5 (9/6.1) | [0.9, 2.4] | 0.78 | [0.57, 1.04] | 0.76 (16/21) | [0.62, 0.95] |
| Ethnicity: Hispanic or Latino, Race: White | 12 | 0.33 (4/12) | [0.1, 0.65] | 0.75 (3/4) | [0.5, 1.5] | 0.88 (7/8) | [0.75, 1.15] | 0.75 (3/4) | [0.5, 1.26] | 1.5 (4/2.7) | [0.7, 3.3] | 0.81 | [0.62, 1.08] | 0.83 (10/12) | [0.67, 1.08] |
| Ethnicity: Not Hispanic or Latino, Race: Native Hawaiian or Other Pacific Islander | 10 | 0.4 (4/10) | [0.12, 0.74] | 0.75 (3/4) | [0.5, 1.25] | 1.0 (6/6) | [1.0, 1.0] | 1.0 (3/3) | [1.0, 1.0] | 1.8 (4/2.3) | [0.8, 3.8] | 0.79 | [0.58, 1.2] | 0.9 (9/10) | [0.8, 1.1] |
| Ethnicity: Not Hispanic or Latino, Race: Other | 10 | 0.5 (5/10) | [0.19, 0.81] | 0.4 (2/5) | [-0.2, 0.8] | 1.0 (5/5) | [1.0, 1.0] | 1.0 (2/2) | [1.0, 1.0] | 1.4 (5/3.5) | [0.8, 2.6] | 0.78 | [0.56, 1.18] | 0.7 (7/10) | [0.4, 1.0] |

**Supplemental Table 19: Stanford HM ACP in Hospital Medicine: Reliability and Fairness Audit by Ethnicity/Race.** Significant differences in prevalence, significantly lower performance, or significantly higher O/E are bolded.

| Group | Sample Size | Prevalence (Fraction) | Prevalence [95% CI] | Sensitivity (Fraction) | Sensitivity [95% CI] | Specificity (Fraction) | Specificity [95% CI] | PPV (Fraction) | PPV [95% CI] | O/E (Fraction) | O/E [95% CI] | AUROC | AUROC [95% CI] | Accuracy (Fraction) | Accuracy [95% CI] |
|---|---|---|---|---|---|---|---|---|---|---|---|---|---|---|---|
| Overall | 225 | 0.44 (99/225) | [0.37, 0.51] | 0.69 (68/99) | [0.6, 0.78] | 0.87 (109/126) | [0.81, 0.93] | 0.8 (68/85) | [0.72, 0.89] | 1.5 (99/65.2) | [1.3, 1.8] | 0.86 | [0.81, 0.91] | 0.79 (177/225) | [0.73, 0.84] |
| Ethnicity: Not Hispanic or Latino, | 43 | 0.53 (23/43) | [0.38, 0.69] | 0.65 (15/23) | [0.47, 0.85] | 0.85 (17/20) | [0.7, 1.05] | 0.83 (15/18) | [0.67, 1.01] | 1.8 (23/12.5) | [1.4, 2.4] | 0.84 | [0.74, 0.97] | 0.74 (32/43) | [0.63, 0.86] |

| | | | | | | | | | | | | | | | |
|---|---|---|---|---|---|---|---|---|---|---|---|---|---|---|---|
| Race: White, Sex: Female | | | | | | | | | | | | | | | |
| Ethnicity: Not Hispanic or Latino, Race: White, Sex: Male | 38 | 0.34 (13/38) | [0.2, 0.51] | 0.69 (9/13) | [0.46, 0.96] | 0.96 (24/25) | [0.92, 1.06] | 0.9 (9/10) | [0.8, 1.13] | 1.7 (13/7.7) | [1.1, 2.6] | 0.94 | [0.89, 1.03] | 0.87 (33/38) | [0.76, 0.97] |
| Ethnicity: Hispanic or Latino, Race: Other, Sex: Female | 22 | 0.18 (4/22) | [0.05, 0.4] | 0.5 (2/4) | [0.0, 1.0] | 0.83 (15/18) | [0.67, 1.0] | 0.4 (2/5) | [-0.2, 0.8] | 0.8 (4/4.8) | [0.3, 2.0] | 0.83 | [0.69, 1.0] | 0.77 (17/22) | [0.64, 0.95] |
| Ethnicity: Not Hispanic or Latino, Race: Asian, Sex: Male | 21 | **0.81 (17/21)** | **[0.58, 0.95]** | 0.65 (11/17) | [0.42, 0.87] | 0.75 (3/4) | [0.5, 1.25] | 0.92 (11/12) | [0.83, 1.12] | 1.8 (17/9.6) | [1.4, 2.2] | 0.87 | [0.74, 1.14] | 0.67 (14/21) | [0.48, 0.86] |
| Ethnicity: Hispanic or Latino, Race: Other, Sex: Male | 16 | 0.12 (2/16) | [0.02, 0.38] | **0.0 (0/2)** | **[0.0, 0.0]** | 0.86 (12/14) | [0.71, 1.05] | **0.0 (0/2)** | **[0.0, 0.0]** | 0.9 (2/2.2) | [0.3, 3.4] | 0.38 | [0.08, 0.62] | 0.75 (12/16) | [0.56, 0.94] |
| Ethnicity: Not Hispanic or Latino, Race: Asian, Sex: Female | 16 | 0.56 (9/16) | [0.3, 0.8] | 0.89 (8/9) | [0.78, 1.14] | 1.0 (7/7) | [1.0, 1.0] | 1.0 (8/8) | [1.0, 1.0] | 1.4 (9/6.4) | [0.9, 2.2] | 1 | [1.0, 1.0] | 0.94 (15/16) | [0.88, 1.06] |
| Ethnicity: Not Hispanic or Latino, Race: Black or African American, Sex: Female | 12 | 0.67 (8/12) | [0.35, 0.9] | 0.75 (6/8) | [0.5, 1.07] | 0.75 (3/4) | [0.5, 1.3] | 0.86 (6/7) | [0.71, 1.14] | 1.7 (8/4.8) | [1.1, 2.5] | 0.78 | [0.56, 1.12] | 0.75 (9/12) | [0.5, 1.0] |
| Ethnicity: Not Hispanic or Latino, Race: Black or African American, Sex: Male | 9 | 0.11 (1/9) | [0.0, 0.48] | **0.0 (0/1)** | **[0.0, 0.0]** | 0.88 (7/8) | [0.75, 1.12] | **0.0 (0/1)** | **[0.0, 0.0]** | 0.8 (1/1.3) | [0.1, 5.0] | 0.25 | [-0.07, 0.5] | 0.78 (7/9) | [0.56, 1.11] |
| Ethnicity: Hispanic or Latino, Race: White, Sex: Male | 7 | 0.29 (2/7) | [0.04, 0.71] | 0.5 (1/2) | [0.0, 1.0] | 1.0 (5/5) | [1.0, 1.0] | 1.0 (1/1) | [1.0, 1.0] | 2.0 (2/1.0) | [0.6, 6.3] | 0.9 | [0.8, 1.15] | 0.86 (6/7) | [0.71, 1.14] |
| Ethnicity: Not Hispanic or Latino, Race: Native Hawaiian or Other | 6 | 0.17 (1/6) | [0.0, 0.64] | **0.0 (0/1)** | **[0.0, 0.0]** | 1.0 (5/5) | [1.0, 1.0] | nan (0/0) | N/A | 0.8 (1/1.2) | [0.1, 5.0] | 0.2 | [0.0, 0.4] | 0.83 (5/6) | [0.67, 1.17] |

| | | | | | | | | | | | | | | | |
|---|---|---|---|---|---|---|---|---|---|---|---|---|---|---|---|
| Pacific Islander, Sex: Female | | | | | | | | | | | | | | | |
| Ethnicity: Not Hispanic or Latino, Race: Other, Sex: Male | 6 | 0.5 (3/6) | [0.12, 0.88] | **0.0 (0/3)** | **[0.0, 0.0]** | 1.0 (3/3) | [1.0, 1.0] | nan (0/0) | N/A | 2.3 (3/1.3) | [1.0, 5.0] | 0.39 | [-0.22, 0.78] | 0.5 (3/6) | [0.17, 0.83] |
| Ethnicity: Hispanic or Latino, Race: White, Sex: Female | 5 | 0.4 (2/5) | [0.05, 0.85] | 1.0 (2/2) | [1.0, 1.0] | 0.67 (2/3) | [0.33, 1.33] | 0.67 (2/3) | [0.33, 1.33] | 1.2 (2/1.7) | [0.4, 3.4] | 0.67 | [0.33, 1.33] | 0.8 (4/5) | [0.6, 1.2] |
| Ethnicity: Not Hispanic or Latino, Race: Native Hawaiian or Other Pacific Islander, Sex: Male | 4 | 0.75 (3/4) | [0.19, 0.99] | 1.0 (3/3) | [1.0, 1.0] | 1.0 (1/1) | [1.0, 1.0] | 1.0 (3/3) | [1.0, 1.0] | 2.9 (3/1.1) | [1.6, 5.0] | 1 | [1.0, 1.0] | 1.0 (4/4) | [1.0, 1.0] |
| Ethnicity: Not Hispanic or Latino, Race: Other, Sex: Female | 4 | 0.5 (2/4) | [0.07, 0.93] | 1.0 (2/2) | [1.0, 1.0] | 1.0 (2/2) | [1.0, 1.0] | 1.0 (2/2) | [1.0, 1.0] | 0.9 (2/2.2) | [0.3, 2.4] | 1 | [1.0, 1.0] | 1.0 (4/4) | [1.0, 1.0] |

**Supplemental Table 20: Stanford HM ACP in Hospital Medicine: Reliability and Fairness Audit by Ethnicity/Race and Sex.** Significant differences in prevalence, significantly lower performance, or significantly higher O/E are bolded.

*Clinical Decision Maker Survey Responses*

| Theme | Example Response | Response Count |
|---|---|---|
| Primary Care: Excitement | "I was impressed that the PPV and specificity were so high. Very encouraging and exciting! I think it will work well for our intended purpose!" | 2 |
| Primary Care: TrustToUseForPurpose | "The results are really exciting! They make sense clinically and I appreciate that in the context of implementation, I would be able to trust the flag as being accurate/helpful at guiding my decision to have more focused, in-depth ACP discussions with the patient and their family/caregivers. " | 2 |
| Inpatient Oncology: Low Sample Size | "Number too small " | 2 |
| Inpatient Oncology: Results Depend On Threshold | "Reliability depends on the model threshold picked, There may be some signals of differences based on age and race/ethnicity groups, but I wonder if this is in part limited by low power." | 2 |
| Inpatient Oncology: EOL Underpredicted Death Risk | "Epic end of life index underpredicted death risk for cancer patients (~30 out of 150 medonc inpatients being flagged at high risk of death is way too low!). ACP model results looked pretty good. Comparison to physicians' predictions and not actual death data limits conclusions since we don't know how good the clinicians' performance is, whether their results are biased for certain ethnic groups etc." | 1 |
| Inpatient Oncology: Reassured that Reliable/Fair | "It’s reassuring that there are these safeguards to ensure that models are reliable and fair. " | 1 |

| | | |
|---|---|---|
| Safeguards Exist | | |
| Inpatient Oncology: Low Sample Size: Affects Detecting Differences By Race | "Reliability depends on the model threshold picked, There may be some signals of differences based on age and race/ethnicity groups, but I wonder if this is in part limited by low power." | 1 |
| Inpatient Oncology: ACP Not Specific At Threshold | "It was not surprising that the ACP model was not specific and made me wonder if we might adjust that threshold " | 1 |
| Hospital Medicine: Interesting | "Interesting!" | 3 |
| Hospital Medicine: Interesting: Clinician Difference By Race | "I found it was interesting how the model and clinicians agreed at the extremes of populations. It was also interesting to see that as a clinician, our predictions are different across races (ie. LatinX more likely to be surprised and Asians less likely). " | 1 |
| Hospital Medicine: Interesting: Sensitivity Difference By Race | "interesting that there was a difference in sensitivity by race " | 1 |

| | | |
|---|---|---|
| Hospital Medicine: Interesting: Models and Clinicians Agree at Extremes Of Populations | "I found it was interesting how the model and clinicians agreed at the extremes of populations. It was also interesting to see that as a clinician, our predictions are different across races (ie. LatinX more likely to be surprised and Asians less likely). " | 1 |

**Supplemental Table 21: Survey responses to "What are the first thoughts that came to your mind on seeing the results of the reliability and fairness audit?"**

| Theme | Example Response | Response Count |
|---|---|---|
| More reliable race data in EHR | "I wish Epic had more reliable race data" | 2 |
| Link subgroup analysis with population demographics ("Model Performance for subgroup X (X's % of patient population") | "I would like to know how the subgroup analysis - particularly the race/ethnicity analyses - relate to our population demographics as a whole. For example, the model is less likely to identify X type of patients, these patients make up X% of your patient population. " | 1 |
| Model's ranking of important patients | "Some statistic to describe rank order" | 1 |
| Broader audit in collaboration with other centers | "Broader effort with other centers " | 1 |

| | | |
|---|---|---|
| Subgroup analysis by clinical characteristic (cancer type, performance status, etc.) | "Performance for patients with varying clinical characteristics (cancer type, performance status, etc.). Performance for patients newer to Stanford system or more established." | 1 |
| Subgroup analysis by patient's time with health system (newer or more established) | "Performance for patients with varying clinical characteristics (cancer type, performance status, etc.). Performance for patients newer to Stanford system or more established." | 1 |

**Supplemental Table 22: Survey responses to "Is there any other information you would want included in this audit to support your decision on whether to deploy a model? If so, what?"**

| **Drivers to make these reliability and fairness audits standard practice** | **Responses** |
|---|---|
| Findings that AI models are not fair | 10 |
| Findings that AI models are not reliable | 9 |
| Academic medicine's push toward racial equity | 9 |

**Supplemental Table 23: Survey responses to "What are some key drivers to making these reliability and fairness audits standard practice?"**

| **Barriers to make these reliability and fairness audits standard practice** | **Responses** |
|---|---|
| Poor demographic data quality | 8 |
| Poor data quality | 6 |
| Lack of data access | 5 |
| Audits are not built into our incentives | 4 |
| Lack of knowledge about how to do an audit | 3 |
| The reliability of deployed AI models is not prioritized | 3 |
| The fairness of deployed AI models is not prioritized | 3 |
| Lack of data science expertise in my practice setting | 2 |
| Other: I don't see us as designing them, but if teams want to engage providers in helping with these audits, I think the most significant barrier is the time, but if there is incentive/appreciation/protected time to do the audit, I can't think of any other barriers | 1 |
| Other: Death data | 1 |
| I do not see any barriers to making reliability and fairness audits standard practice. | 0 |

**Supplemental Table 24: Survey responses to "What are some key barriers to making these reliability and fairness audits standard practice?"**

| Pros in using AI to support my work | Responses |
|---|---|
| Helps triage patients and identify who would benefit the most | 10 |
| Shared understanding of patients for our whole care team | 9 |
| Reduces work for me | 3 |
| I do not see any pros to using an AI model to support my work. | 0 |

**Supplemental Table 25: Survey responses to "As a clinical decisionmaker, what pros do you see in using an AI model to support your work?"**

| Cons in using AI to support my work | Responses |
|---|---|
| Lack of transparency of the model | 5 |
| Takes effort to maintain | 4 |
| I disagree with the model | 3 |
| Loss of my decisionmaking autonomy | 2 |
| Pressure to act even if I disagree with the model | 1 |
| Other: Worry that the model may miss some patients who might benefit | 1 |

| | |
|---|---|
| Other: The HM model although is more sensitive- so many patients flag. Is it possible to risk stratify who is highest risk (using green, yellow, red) like the Epic AI models | 0 |
| I do not see any cons to using an AI model to support my work. | 1 |

**Supplemental Table 26: Survey responses to "As a clinical decisionmaker, what cons do you see in using an AI model to support your work?"**

## Class-Balanced Analysis

*Epic EOL Low Threshold in Primary Care*

| Group | Sample Size | Prevalence (Fraction) | Prevalence [95% CI] | Sensitivity (Fraction) | Sensitivity [95% CI] | Specificity (Fraction) | Specificity [95% CI] | Positive Predictive Value (Fraction) | Positive Predictive Value [95% CI] | O/E (Fraction) | O/E [95% CI] |
|---|---|---|---|---|---|---|---|---|---|---|---|
| Overall | 540 | 0.5 (270/540) | [0.46, 0.54] | 0.38 (102/270) | [0.32, 0.44] | 0.98 (265/270) | [0.97, 1.0] | 0.95 (102/107) | [0.92, 1.0] | 5.3 (270/50.5) | [4.9, 5.8] |
| Sex: Female | 325 | 0.5 (163/325) | [0.45, 0.56] | 0.35 (57/163) | [0.27, 0.42] | 0.98 (158/162) | [0.96, 1.0] | 0.93 (57/61) | [0.88, 1.01] | 5.6 (163/29.1) | [5.0, 6.2] |
| Sex: Male | 215 | 0.5 (107/215) | [0.43, 0.57] | 0.42 (45/107) | [0.32, 0.51] | 0.99 (107/108) | [0.98, 1.01] | 0.98 (45/46) | [0.96, 1.03] | 5.0 (107/21.4) | [4.4, 5.7] |

**Supplemental Table 27: Epic EOL Low Threshold in Primary Care: Class Balanced Reliability and Fairness Audit by Sex.** Significant differences in prevalence, significantly lower performance, or significantly higher O/E are bolded.

| Group | Sample Size | Prevalence (Fraction) | Prevalence [95% CI] | Sensitivity (Fraction) | Sensitivity [95% CI] | Specificity (Fraction) | Specificity [95% CI] | Positive Predictive Value (Fraction) | Positive Predictive Value [95% CI] | O/E (Fraction) | O/E [95% CI] |
|---|---|---|---|---|---|---|---|---|---|---|---|
| Overall | 540 | 0.5 (270/540) | [0.46, 0.54] | 0.38 (102/270) | [0.32, 0.44] | 0.98 (265/270) | [0.97, 1.0] | 0.95 (102/107) | [0.92, 1.0] | 5.3 (270/50.5) | [4.9, 5.8] |
| Age: (10, 20] | 3 | 0.0 (0/3) | [0, 0.71] | nan (0/0) | N/A | 1.0 (3/3) | [1.0, 1.0] | nan (0/0) | N/A | nan (0/0.0) | N/A |
| Age: (20, 30] | 27 | 0.0 (0/27) | [0, 0.13] | nan (0/0) | N/A | 1.0 (27/27) | [1.0, 1.0] | nan (0/0) | N/A | nan (0/0.0) | N/A |

| | | | | | | | | | | | |
|---|---|---|---|---|---|---|---|---|---|---|---|
| Age: (30, 40] | 61 | 0.0 (0/61) | [0, 0.06] | nan (0/0) | N/A | 1.0 (61/61) | [1.0, 1.0] | nan (0/0) | N/A | 0.0 (0/0.0) | N/A |
| Age: (40, 50] | 54 | **0.3 (16/54)** | **[0.18, 0.44]** | 0.19 (3/16) | [-0.04, 0.38] | 1.0 (38/38) | [1.0, 1.0] | 1.0 (3/3) | [1.0, 1.0] | **15.0 (16/1.1)** | **[9.9, 22.6]** |
| Age: (50, 60] | 53 | **0.13 (7/53)** | **[0.05, 0.25]** | **0.0 (0/7)** | **[0.0, 0.0]** | 1.0 (46/46) | [1.0, 1.0] | nan (0/0) | N/A | **14.0 (7/0.5)** | **[7.0, 27.9]** |
| Age: (60, 70] | 76 | 0.46 (35/76) | [0.35, 0.58] | **0.09 (3/35)** | **[-0.02, 0.17]** | 1.0 (41/41) | [1.0, 1.0] | 1.0 (3/3) | [1.0, 1.0] | **19.3 (35/1.8)** | **[15.2, 24.7]** |
| Age: (70, 80] | 96 | 0.62 (60/96) | [0.52, 0.72] | **0.1 (6/60)** | **[0.02, 0.17]** | 0.97 (35/36) | [0.94, 1.03] | 0.86 (6/7) | [0.71, 1.16] | **9.8 (60/6.2)** | **[8.4, 11.4]** |
| Age: (80, 90] | 95 | **0.84 (80/95)** | **[0.75, 0.91]** | 0.41 (33/80) | [0.31, 0.52] | 0.87 (13/15) | [0.73, 1.05] | 0.94 (33/35) | [0.89, 1.03] | 4.3 (80/18.5) | [4.0, 4.7] |
| Age: (90, 100] | 66 | **0.95 (63/66)** | **[0.87, 0.99]** | 0.76 (48/63) | [0.66, 0.87] | **0.33 (1/3)** | **[-0.33, 0.67]** | 0.96 (48/50) | [0.92, 1.02] | 3.4 (63/18.5) | [3.2, 3.6] |
| Age: (100, 110] | 9 | **1.0 (9/9)** | **[0.66, 1]** | 1.0 (9/9) | [1.0, 1.0] | nan (0/0) | N/A | 1.0 (9/9) | [1.0, 1.0] | 2.3 (9/4.0) | [2.3, 2.3] |

**Supplemental Table 28: Epic EOL Low Threshold in Primary Care: Class Balanced Reliability and Fairness Audit by Age.** Significant differences in prevalence, significantly lower performance, or significantly higher O/E are bolded.

| Group | Sample Size | Prevalence (Fraction) | Prevalence [95% CI] | Sensitivity (Fraction) | Sensitivity [95% CI] | Specificity (Fraction) | Specificity [95% CI] | Positive Predictive Value (Fraction) | Positive Predictive Value [95% CI] | O/E (Fraction) | O/E [95% CI] |
|---|---|---|---|---|---|---|---|---|---|---|---|
| Overall | 540 | 0.5 (270/540) | [0.46, 0.54] | 0.38 (102/270) | [0.32, 0.44] | 0.98 (265/270) | [0.97, 1.0] | 0.95 (102/107) | [0.92, 1.0] | 5.3 (270/50.5) | [4.9, 5.8] |

| | | | | | | | | | | | |
|---|---|---|---|---|---|---|---|---|---|---|---|
| Ethnicity: Not Hispanic or Latino, Race: White | 259 | 0.56 (144/259) | [0.49, 0.62] | 0.44 (63/144) | [0.35, 0.52] | 0.97 (112/115) | [0.95, 1.0] | 0.95 (63/66) | [0.91, 1.01] | 5.0 (144/28.7) | [4.5, 5.6] |
| Ethnicity: Not Hispanic or Latino, Race: Asian | 173 | 0.52 (90/173) | [0.44, 0.6] | 0.32 (29/90) | [0.22, 0.42] | 0.99 (82/83) | [0.98, 1.01] | 0.97 (29/30) | [0.93, 1.05] | 6.4 (90/14.0) | [5.6, 7.4] |
| Ethnicity: Not Hispanic or Latino, Race: Other | 42 | 0.57 (24/42) | [0.41, 0.72] | 0.42 (10/24) | [0.22, 0.61] | 0.94 (17/18) | [0.89, 1.07] | 0.91 (10/11) | [0.82, 1.12] | 3.4 (24/7.0) | [2.7, 4.5] |
| Ethnicity: Hispanic or Latino, Race: Other | 21 | **0.1 (2/21)** | **[0.01, 0.3]** | **0.0 (0/2)** | **[0.0, 0.0]** | 1.0 (19/19) | [1.0, 1.0] | nan (0/0) | N/A | 7.4 (2/0.3) | [2.0, 27.7] |
| Ethnicity: Not Hispanic or Latino, Race: Black or African American | 9 | **0.0 (0/9)** | **[0, 0.34]** | nan (0/0) | N/A | 1.0 (9/9) | [1.0, 1.0] | nan (0/0) | N/A | 0.0 (0/0.2) | N/A |
| Ethnicity: Not Hispanic or Latino, Race: Native Hawaiian or Other Pacific Islander | 9 | 0.78 (7/9) | [0.4, 0.97] | **0.0 (0/7)** | **[0.0, 0.0]** | 1.0 (2/2) | [1.0, 1.0] | nan (0/0) | N/A | **87.5 (7/0.1)** | **[61.7, 124.1]** |
| Ethnicity: Hispanic or Latino, Race: White | 5 | 0.0 (0/5) | [0, 0.52] | nan (0/0) | N/A | 1.0 (5/5) | [1.0, 1.0] | nan (0/0) | N/A | 0.0 (0/0.0) | N/A |
| Ethnicity: Not Hispanic or Latino, Race: Unknown | 3 | 0.0 (0/3) | [0, 0.71] | nan (0/0) | N/A | 1.0 (3/3) | [1.0, 1.0] | nan (0/0) | N/A | 0.0 (0/0.0) | N/A |
| Ethnicity: Hispanic or Latino, Race: Asian | 2 | 0.0 (0/2) | [0, 0.84] | nan (0/0) | N/A | 1.0 (2/2) | [1.0, 1.0] | nan (0/0) | N/A | nan (0/0.0) | N/A |
| Ethnicity: Not Hispanic or Latino, Race: American Indian or Alaska Native | 1 | 0.0 (0/1) | [0, 0.98] | nan (0/0) | N/A | 1.0 (1/1) | [1.0, 1.0] | nan (0/0) | N/A | 0.0 (0/0.0) | N/A |
| Ethnicity: Not Hispanic or Latino, Race: Patient Refused | 1 | 0.0 (0/1) | [0, 0.98] | nan (0/0) | N/A | 1.0 (1/1) | [1.0, 1.0] | nan (0/0) | N/A | nan (0/0.0) | N/A |

**Supplemental Table 29: Epic EOL Low Threshold in Primary Care: Class Balanced Reliability and Fairness Audit by Ethnicity/Race.** Significant differences in prevalence, significantly lower performance, or significantly higher O/E are bolded.

| Group | Sample Size | Prevalence (Fraction) | Prevalence [95% CI] | Sensitivity (Fraction) | Sensitivity [95% CI] | Specificity (Fraction) | Specificity [95% CI] | Positive Predictive Value (Fraction) | Positive Predictive Value [95% CI] | O/E (Fraction) | O/E [95% CI] |
|---|---|---|---|---|---|---|---|---|---|---|---|
| Overall | 540 | 0.5 (270/540) | [0.46, 0.54] | 0.38 (102/270) | [0.32, 0.44] | 0.98 (265/270) | [0.97, 1.0] | 0.95 (102/107) | [0.92, 1.0] | 5.3 (270/50.5) | [4.9, 5.8] |
| Ethnicity: Not Hispanic or Latino, Race: White, Sex: Female | 150 | 0.55 (82/150) | [0.46, 0.63] | 0.45 (37/82) | [0.34, 0.56] | 0.97 (66/68) | [0.94, 1.02] | 0.95 (37/39) | [0.9, 1.03] | 5.5 (82/15.0) | [4.7, 6.3] |
| Ethnicity: Not Hispanic or Latino, Race: White, Sex: Male | 109 | 0.57 (62/109) | [0.47, 0.66] | 0.42 (26/62) | [0.29, 0.54] | 0.98 (46/47) | [0.96, 1.02] | 0.96 (26/27) | [0.93, 1.04] | 4.5 (62/13.7) | [3.8, 5.3] |
| Ethnicity: Not Hispanic or Latino, Race: Asian, Sex: Female | 105 | 0.52 (55/105) | [0.42, 0.62] | 0.27 (15/55) | [0.15, 0.39] | 0.98 (49/50) | [0.96, 1.03] | 0.94 (15/16) | [0.88, 1.09] | 6.0 (55/9.1) | [5.0, 7.3] |
| Ethnicity: Not Hispanic or Latino, Race: Asian, Sex: Male | 68 | 0.51 (35/68) | [0.39, 0.64] | 0.4 (14/35) | [0.24, 0.56] | 1.0 (33/33) | [1.0, 1.0] | 1.0 (14/14) | [1.0, 1.0] | 7.1 (35/5.0) | [5.6, 8.9] |
| Ethnicity: Not Hispanic or Latino, Race: Other, Sex: Female | 29 | 0.55 (16/29) | [0.36, 0.74] | 0.31 (5/16) | [0.05, 0.53] | 0.92 (12/13) | [0.85, 1.08] | 0.83 (5/6) | [0.67, 1.17] | 3.5 (16/4.6) | [2.5, 4.8] |
| Ethnicity: Not Hispanic or Latino, Race: Other, Sex: Male | 13 | 0.62 (8/13) | [0.32, 0.86] | 0.62 (5/8) | [0.25, 0.98] | 1.0 (5/5) | [1.0, 1.0] | 1.0 (5/5) | [1.0, 1.0] | 3.4 (8/2.4) | [2.2, 5.2] |
| Ethnicity: Hispanic or Latino, Race: Other, Sex: Female | 11 | **0.0 (0/11)** | **[0, 0.28]** | nan (0/0) | N/A | 1.0 (11/11) | [1.0, 1.0] | nan (0/0) | N/A | 0.0 (0/0.1) | N/A |
| Ethnicity: Hispanic or Latino, Race: Other, Sex: Male | 10 | 0.2 (2/10) | [0.03, 0.56] | **0.0 (0/2)** | **[0.0, 0.0]** | 1.0 (8/8) | [1.0, 1.0] | nan (0/0) | N/A | 11.8 (2/0.2) | [3.4, 40.6] |

| | | | | | | | | | | | |
|---|---|---|---|---|---|---|---|---|---|---|---|
| Ethnicity: Not Hispanic or Latino, Race: Native Hawaiian or Other Pacific Islander, Sex: Female | 9 | 0.78 (7/9) | [0.4, 0.97] | **0.0 (0/7)** | **[0.0, 0.0]** | 1.0 (2/2) | [1.0, 1.0] | nan (0/0) | N/A | **87.5 (7/0.1)** | **[61.7, 124.1]** |
| Ethnicity: Not Hispanic or Latino, Race: Black or African American, Sex: Female | 7 | **0.0 (0/7)** | **[0, 0.41]** | nan (0/0) | N/A | 1.0 (7/7) | [1.0, 1.0] | nan (0/0) | N/A | 0.0 (0/0.2) | N/A |
| Ethnicity: Hispanic or Latino, Race: White, Sex: Male | 4 | 0.0 (0/4) | [0, 0.6] | nan (0/0) | N/A | 1.0 (4/4) | [1.0, 1.0] | nan (0/0) | N/A | 0.0 (0/0.0) | N/A |
| Ethnicity: Not Hispanic or Latino, Race: Black or African American, Sex: Male | 2 | 0.0 (0/2) | [0, 0.84] | nan (0/0) | N/A | 1.0 (2/2) | [1.0, 1.0] | nan (0/0) | N/A | 0.0 (0/0.0) | N/A |
| Ethnicity: Not Hispanic or Latino, Race: Unknown, Sex: Male | 2 | 0.0 (0/2) | [0, 0.84] | nan (0/0) | N/A | 1.0 (2/2) | [1.0, 1.0] | nan (0/0) | N/A | 0.0 (0/0.0) | N/A |
| Ethnicity: Hispanic or Latino, Race: Asian, Sex: Female | 2 | 0.0 (0/2) | [0, 0.84] | nan (0/0) | N/A | 1.0 (2/2) | [1.0, 1.0] | nan (0/0) | N/A | nan (0/0.0) | N/A |
| Ethnicity: Hispanic or Latino, Race: White, Sex: Female | 1 | 0.0 (0/1) | [0, 0.98] | nan (0/0) | N/A | 1.0 (1/1) | [1.0, 1.0] | nan (0/0) | N/A | nan (0/0.0) | N/A |
| Ethnicity: Not Hispanic or Latino, Race: Unknown, Sex: Female | 1 | 0.0 (0/1) | [0, 0.98] | nan (0/0) | N/A | 1.0 (1/1) | [1.0, 1.0] | nan (0/0) | N/A | nan (0/0.0) | N/A |
| Ethnicity: Not Hispanic or Latino, Race: American Indian or Alaska Native, Sex: Male | 1 | 0.0 (0/1) | [0, 0.98] | nan (0/0) | N/A | 1.0 (1/1) | [1.0, 1.0] | nan (0/0) | N/A | 0.0 (0/0.0) | N/A |
| Ethnicity: Not Hispanic or Latino, Race: Patient Refused, Sex: Male | 1 | 0.0 (0/1) | [0, 0.98] | nan (0/0) | N/A | 1.0 (1/1) | [1.0, 1.0] | nan (0/0) | N/A | nan (0/0.0) | N/A |

**Supplemental Table 30: Epic EOL Low Threshold in Primary Care: Class Balanced Reliability and Fairness Audit by Ethnicity/Race and Sex.** Significant differences in prevalence, significantly lower performance, or significantly higher O/E are bolded.

## *Epic EOL High Threshold in Inpatient Oncology*

| Group | Sample Size | Prevalence (Fraction) | Prevalence [95% CI] | Sensitivity (Fraction) | Sensitivity [95% CI] | Specificity (Fraction) | Specificity [95% CI] | Positive Predictive Value (Fraction) | Positive Predictive Value [95% CI] | O/E (Fraction) | O/E [95% CI] |
|---|---|---|---|---|---|---|---|---|---|---|---|
| Overall | 210 | 0.5 (105/210) | [0.43, 0.57] | 0.27 (28/105) | [0.18, 0.35] | 0.89 (93/105) | [0.82, 0.95] | 0.7 (28/40) | [0.56, 0.84] | 2.4 (105/44.5) | [2.1, 2.7] |
| Sex: Female | 88 | 0.5 (44/88) | [0.39, 0.61] | 0.27 (12/44) | [0.14, 0.4] | 1.0 (44/44) | [1.0, 1.0] | 1.0 (12/12) | [1.0, 1.0] | 3.0 (44/14.7) | [2.4, 3.7] |
| Sex: Male | 122 | 0.5 (61/122) | [0.41, 0.59] | 0.26 (16/61) | [0.15, 0.37] | 0.8 (49/61) | [0.71, 0.92] | 0.57 (16/28) | [0.39, 0.76] | 2.0 (61/29.8) | [1.7, 2.4] |

**Supplemental Table 31: Epic EOL High Threshold in Inpatient Oncology: Class Balanced Reliability and Fairness Audit by Sex.** Prevalence, performance and calibration is presented for the overall cohort and for subgroups with significant differences in prevalence, significantly lower performance, or significantly higher O/E (bolded).

| Group | Sample Size | Prevalence (Fraction) | Prevalence [95% CI] | Sensitivity (Fraction) | Sensitivity [95% CI] | Specificity (Fraction) | Specificity [95% CI] | Positive Predictive Value (Fraction) | Positive Predictive Value [95% CI] | O/E (Fraction) | O/E [95% CI] |
|---|---|---|---|---|---|---|---|---|---|---|---|
| Overall | 210 | 0.5 (105/210) | [0.43, 0.57] | 0.27 (28/105) | [0.18, 0.35] | 0.89 (93/105) | [0.82, 0.95] | 0.7 (28/40) | [0.56, 0.84] | 2.4 (105/44.5) | [2.1, 2.7] |

| | | | | | | | | | | | |
|---|---|---|---|---|---|---|---|---|---|---|---|
| Age: (20, 30] | 28 | **0.11 (3/28)** | **[0.02, 0.28]** | **0.0 (0/3)** | **[0.0, 0.0]** | 1.0 (25/25) | [1.0, 1.0] | nan (0/0) | N/A | 2.3 (3/1.3) | [0.8, 6.7] |
| Age: (30, 40] | 21 | 0.38 (8/21) | [0.18, 0.62] | **0.0 (0/8)** | **[0.0, 0.0]** | 1.0 (13/13) | [1.0, 1.0] | nan (0/0) | N/A | **4.9 (8/1.6)** | **[2.9, 8.5]** |
| Age: (40, 50] | 20 | 0.4 (8/20) | [0.19, 0.64] | 0.12 (1/8) | [-0.15, 0.25] | 1.0 (12/12) | [1.0, 1.0] | 1.0 (1/1) | [1.0, 1.0] | 3.5 (8/2.3) | [2.1, 6.1] |
| Age: (50, 60] | 39 | 0.46 (18/39) | [0.3, 0.63] | 0.28 (5/18) | [0.06, 0.46] | 1.0 (21/21) | [1.0, 1.0] | 1.0 (5/5) | [1.0, 1.0] | 2.4 (18/7.4) | [1.7, 3.4] |
| Age: (60, 70] | 41 | 0.71 (29/41) | [0.54, 0.84] | 0.24 (7/29) | [0.08, 0.39] | **0.33 (4/12)** | **[0.04, 0.58]** | 0.47 (7/15) | [0.21, 0.72] | 2.2 (29/13.5) | [1.8, 2.6] |
| Age: (70, 80] | 41 | 0.59 (24/41) | [0.42, 0.74] | 0.38 (9/24) | [0.17, 0.58] | 0.76 (13/17) | [0.59, 0.98] | 0.69 (9/13) | [0.46, 0.96] | 2.1 (24/11.5) | [1.6, 2.7] |
| Age: (80, 90] | 18 | 0.72 (13/18) | [0.47, 0.9] | 0.38 (5/13) | [0.1, 0.63] | 1.0 (5/5) | [1.0, 1.0] | 1.0 (5/5) | [1.0, 1.0] | 2.2 (13/5.9) | [1.7, 2.9] |
| Age: (90, 100] | 2 | 1.0 (2/2) | [0.16, 1] | 0.5 (1/2) | [0.0, 1.0] | nan (0/0) | N/A | 1.0 (1/1) | [1.0, 1.0] | **1.8 (2/1.1)** | **[1.8, 1.8]** |

**Supplemental Table 32: Epic EOL High Threshold in Inpatient Oncology: Class Balanced Reliability and Fairness Audit by Age.** Significant differences in prevalence, significantly lower performance, or significantly higher O/E are bolded.

| Group | Sample Size | Prevalence (Fraction) | Prevalence [95% CI] | Sensitivity (Fraction) | Sensitivity [95% CI] | Specificity (Fraction) | Specificity [95% CI] | Positive Predictive Value (Fraction) | Positive Predictive Value [95% CI] | O/E (Fraction) | O/E [95% CI] |
|---|---|---|---|---|---|---|---|---|---|---|---|

| | | | | | | | | | | | |
|---|---|---|---|---|---|---|---|---|---|---|---|
| Overall | 210 | 0.5 (105/210) | [0.43, 0.57] | 0.27 (28/105) | [0.18, 0.35] | 0.89 (93/105) | [0.82, 0.95] | 0.7 (28/40) | [0.56, 0.84] | 2.4 (105/44.5) | [2.1, 2.7] |
| Ethnicity: Not Hispanic or Latino, Race: White | 82 | 0.46 (38/82) | [0.35, 0.58] | 0.24 (9/38) | [0.1, 0.36] | 0.8 (35/44) | [0.68, 0.92] | 0.5 (9/18) | [0.27, 0.73] | 1.8 (38/21.2) | [1.4, 2.3] |
| Ethnicity: Not Hispanic or Latino, Race: Asian | 46 | 0.67 (31/46) | [0.52, 0.8] | 0.39 (12/31) | [0.2, 0.55] | 1.0 (15/15) | [1.0, 1.0] | 1.0 (12/12) | [1.0, 1.0] | 2.6 (31/12.1) | [2.1, 3.1] |
| Ethnicity: Hispanic or Latino, Race: Other | 45 | 0.49 (22/45) | [0.34, 0.64] | **0.09 (2/22)** | **[-0.05, 0.18]** | 1.0 (23/23) | [1.0, 1.0] | 1.0 (2/2) | [1.0, 1.0] | **5.3 (22/4.1)** | **[4.0, 7.2]** |
| Ethnicity: Not Hispanic or Latino, Race: Other | 19 | 0.47 (9/19) | [0.24, 0.71] | 0.33 (3/9) | [0.0, 0.67] | 1.0 (10/10) | [1.0, 1.0] | 1.0 (3/3) | [1.0, 1.0] | 2.8 (9/3.2) | [1.8, 4.5] |
| Ethnicity: Not Hispanic or Latino, Race: Black or African American | 9 | 0.22 (2/9) | [0.03, 0.6] | 0.5 (1/2) | [0.0, 1.0] | 0.57 (4/7) | [0.14, 0.98] | **0.25 (1/4)** | **[-0.5, 0.5]** | 0.8 (2/2.6) | [0.2, 2.6] |
| Ethnicity: Hispanic or Latino, Race: White | 5 | 0.2 (1/5) | [0.01, 0.72] | **0.0 (0/1)** | **[0.0, 0.0]** | 1.0 (4/4) | [1.0, 1.0] | nan (0/0) | N/A | 1.5 (1/0.7) | [0.3, 8.5] |
| Ethnicity: Hispanic or Latino, Race: American Indian or Alaska Native | 2 | 0.0 (0/2) | [0, 0.84] | nan (0/0) | N/A | 1.0 (2/2) | [1.0, 1.0] | nan (0/0) | N/A | 0.0 (0/0.0) | N/A |
| Ethnicity: Not Hispanic or Latino, Race: American Indian or Alaska Native | 1 | 1.0 (1/1) | [0.03, 1] | 1.0 (1/1) | [1.0, 1.0] | nan (0/0) | N/A | 1.0 (1/1) | [1.0, 1.0] | 1.6 (1/0.6) | [1.6, 1.6] |

**Supplemental Table 33: Epic EOL High Threshold in Inpatient Oncology: Class Balanced Reliability and Fairness Audit by Ethnicity/Race.** Significant differences in prevalence, significantly lower performance, or significantly higher O/E are bolded.

| Group | Sample | Prevalence | Prevalence [95% | Sensitivity | Sensitivity | Specificity | Specificity | Positive Predictive | Positive Predictive | O/E (Frac | O/E [95% |
|---|---|---|---|---|---|---|---|---|---|---|---|

| | Size | (Fraction) | CI] | (Fraction) | [95% CI] | (Fraction) | [95% CI] | Value (Fraction) | Value [95% CI] | tion) | CI] |
|---|---|---|---|---|---|---|---|---|---|---|---|
| Overall | 210 | 0.5 (105/210) | [0.43, 0.57] | 0.27 (28/105) | [0.18, 0.35] | 0.89 (93/105) | [0.82, 0.95] | 0.7 (28/40) | [0.56, 0.84] | 2.4 (105/44.5) | [2.1, 2.7] |
| Ethnicity: Not Hispanic or Latino, Race: White, Sex: Male | 44 | 0.5 (22/44) | [0.35, 0.65] | 0.23 (5/22) | [0.03, 0.4] | **0.59 (13/22)** | **[0.37, 0.79]** | 0.36 (5/14) | [0.07, 0.61] | 1.6 (22/13.9) | [1.2, 2.1] |
| Ethnicity: Not Hispanic or Latino, Race: White, Sex: Female | 38 | 0.42 (16/38) | [0.26, 0.59] | 0.25 (4/16) | [0.03, 0.44] | 1.0 (22/22) | [1.0, 1.0] | 1.0 (4/4) | [1.0, 1.0] | 2.2 (16/7.3) | [1.5, 3.2] |
| Ethnicity: Not Hispanic or Latino, Race: Asian, Sex: Male | 28 | 0.68 (19/28) | [0.48, 0.84] | 0.37 (7/19) | [0.15, 0.58] | 1.0 (9/9) | [1.0, 1.0] | 1.0 (7/7) | [1.0, 1.0] | 2.4 (19/8.0) | [1.8, 3.1] |
| Ethnicity: Hispanic or Latino, Race: Other, Sex: Female | 23 | 0.39 (9/23) | [0.2, 0.61] | 0.22 (2/9) | [-0.06, 0.44] | 1.0 (14/14) | [1.0, 1.0] | 1.0 (2/2) | [1.0, 1.0] | 4.4 (9/2.1) | [2.6, 7.3] |
| Ethnicity: Hispanic or Latino, Race: Other, Sex: Male | 22 | 0.59 (13/22) | [0.36, 0.79] | **0.0 (0/13)** | **[0.0, 0.0]** | 1.0 (9/9) | [1.0, 1.0] | nan (0/0) | N/A | **6.3 (13/2.1)** | **[4.4, 8.9]** |
| Ethnicity: Not Hispanic or Latino, Race: Asian, Sex: Female | 18 | 0.67 (12/18) | [0.41, 0.87] | 0.42 (5/12) | [0.12, 0.69] | 1.0 (6/6) | [1.0, 1.0] | 1.0 (5/5) | [1.0, 1.0] | 3.0 (12/4.0) | [2.1, 4.1] |
| Ethnicity: Not Hispanic or Latino, Race: Other, Sex: Male | 14 | 0.29 (4/14) | [0.08, 0.58] | 0.5 (2/4) | [0.0, 1.0] | 1.0 (10/10) | [1.0, 1.0] | 1.0 (2/2) | [1.0, 1.0] | 1.8 (4/2.2) | [0.8, 4.2] |
| Ethnicity: Not Hispanic or Latino, Race: Black or African American, Sex: Male | 6 | 0.17 (1/6) | [0.0, 0.64] | 1.0 (1/1) | [1.0, 1.0] | 0.4 (2/5) | [-0.03, 0.8] | **0.25 (1/4)** | **[-0.25, 0.5]** | 0.4 (1/2.4) | [0.1, 2.5] |
| Ethnicity: Not Hispanic or Latino, Race: Other, Sex: Female | 5 | 1.0 (5/5) | [0.48, 1] | 0.2 (1/5) | [-0.2, 0.4] | nan (0/0) | N/A | 1.0 (1/1) | [1.0, 1.0] | 4.9 (5/1.0) | [4.9, 4.9] |
| Ethnicity: Hispanic or Latino, Race: White, Sex: Male | 4 | 0.0 (0/4) | [0, 0.6] | nan (0/0) | N/A | 1.0 (4/4) | [1.0, 1.0] | nan (0/0) | N/A | 0.0 (0/0.7) | N/A |

| | | | | | | | | | | | |
|---|---|---|---|---|---|---|---|---|---|---|---|
| Ethnicity: Not Hispanic or Latino, Race: Black or African American, Sex: Female | 3 | 0.33 (1/3) | [0.01, 0.91] | **0.0 (0/1)** | **[0.0, 0.0]** | 1.0 (2/2) | [1.0, 1.0] | nan (0/0) | N/A | 4.2 (1/0.2) | [0.8, 20.6] |
| Ethnicity: Hispanic or Latino, Race: American Indian or Alaska Native, Sex: Male | 2 | 0.0 (0/2) | [0, 0.84] | nan (0/0) | N/A | 1.0 (2/2) | [1.0, 1.0] | nan (0/0) | N/A | 0.0 (0/0.0) | N/A |
| Ethnicity: Hispanic or Latino, Race: White, Sex: Female | 1 | 1.0 (1/1) | [0.03, 1] | **0.0 (0/1)** | **[0.0, 0.0]** | nan (0/0) | N/A | nan (0/0) | N/A | inf (1/0.0) | [inf, inf] |
| Ethnicity: Not Hispanic or Latino, Race: American Indian or Alaska Native, Sex: Male | 1 | 1.0 (1/1) | [0.03, 1] | 1.0 (1/1) | [1.0, 1.0] | nan (0/0) | N/A | 1.0 (1/1) | [1.0, 1.0] | 1.6 (1/0.6) | [1.6, 1.6] |

**Supplemental Table 34: Epic EOL High Threshold in Inpatient Oncology: Class Balanced Reliability and Fairness Audit by Ethnicity/Race and Sex.** Significant differences in prevalence, significantly lower performance, or significantly higher O/E are bolded.

## *Stanford HM ACP in Inpatient Oncology*

| Group | Sample Size | Prevalence (Fraction) | Prevalence [95% CI] | Sensitivity (Fraction) | Sensitivity [95% CI] | Specificity (Fraction) | Specificity [95% CI] | Positive Predictive Value (Fraction) | Positive Predictive Value [95% CI] | O/E (Fraction) | O/E [95% CI] |
|---|---|---|---|---|---|---|---|---|---|---|---|
| Overall | 158 | 0.5 (79/158) | [0.42, 0.58] | 0.89 (70/79) | [0.82, 0.95] | 0.56 (44/79) | [0.45, 0.67] | 0.67 (70/105) | [0.58, 0.76] | 1.4 (79/57.0) | [1.2, 1.6] |
| Sex: Female | 71 | 0.45 (32/71) | [0.33, 0.57] | 0.91 (29/32) | [0.81, 1.02] | 0.41 (16/39) | [0.25, 0.57] | 0.56 (29/52) | [0.42, 0.7] | 1.2 (32/27.6) | [0.9, 1.5] |
| Sex: Male | 87 | 0.54 (47/87) | [0.43, 0.65] | 0.87 (41/47) | [0.78, 0.97] | 0.7 (28/40) | [0.57, 0.84] | 0.77 (41/53) | [0.67, 0.89] | 1.6 (47/29.3) | [1.3, 1.9] |

**Supplemental Table 35: Stanford HM ACP in Inpatient Oncology: Class Balanced Reliability and Fairness Audit by Sex.** Significant differences in prevalence, significantly lower performance, or significantly higher O/E are bolded.

| Group | Sample Size | Prevalence (Fraction) | Prevalence [95% CI] | Sensitivity (Fraction) | Sensitivity [95% CI] | Specificity (Fraction) | Specificity [95% CI] | Positive Predictive Value (Fraction) | Positive Predictive Value [95% CI] | O/E (Fraction) | O/E [95% CI] |
|---|---|---|---|---|---|---|---|---|---|---|---|
| Overall | 158 | 0.5 (79/158) | [0.42, 0.58] | 0.89 (70/79) | [0.82, 0.95] | 0.56 (44/79) | [0.45, 0.67] | 0.67 (70/105) | [0.58, 0.76] | 1.4 (79/57.0) | [1.2, 1.6] |
| Age: (20, 30] | 19 | **0.16 (3/19)** | **[0.03, 0.4]** | 1.0 (3/3) | [1.0, 1.0] | 0.88 (14/16) | [0.75, 1.06] | 0.6 (3/5) | [0.2, 1.2] | 0.8 (3/3.9) | [0.3, 2.2] |
| Age: (30, 40] | 17 | 0.35 (6/17) | [0.14, 0.62] | 0.83 (5/6) | [0.67, 1.17] | 0.64 (7/11) | [0.36, 0.94] | 0.56 (5/9) | [0.21, 0.89] | 1.2 (6/4.9) | [0.6, 2.4] |
| Age: (40, 50] | 23 | 0.26 (6/23) | [0.1, 0.48] | 0.83 (5/6) | [0.67, 1.17] | **0.18 (3/17)** | **[-0.02, 0.35]** | **0.26 (5/19)** | **[0.07, 0.47]** | 0.8 (6/7.5) | [0.4, 1.6] |
| Age: (50, 60] | 33 | 0.42 (14/33) | [0.25, 0.61] | 0.93 (13/14) | [0.86, 1.09] | 0.74 (14/19) | [0.54, 0.94] | 0.72 (13/18) | [0.51, 0.94] | 1.4 (14/9.8) | [1.0, 2.1] |
| Age: (60, 70] | 27 | **0.85 (23/27)** | **[0.66, 0.96]** | 0.96 (22/23) | [0.91, 1.06] | 0.5 (2/4) | [0.0, 1.0] | 0.92 (22/24) | [0.83, 1.04] | 1.8 (23/13.1) | [1.5, 2.1] |
| Age: (70, 80] | 23 | 0.7 (16/23) | [0.47, 0.87] | 0.75 (12/16) | [0.56, 0.97] | 0.57 (4/7) | [0.14, 0.98] | 0.8 (12/15) | [0.6, 1.03] | 1.7 (16/9.3) | [1.3, 2.3] |
| Age: (80, 90] | 15 | 0.67 (10/15) | [0.38, 0.88] | 0.9 (9/10) | [0.8, 1.1] | **0.0 (0/5)** | **[0.0, 0.0]** | 0.64 (9/14) | [0.42, 0.9] | 1.3 (10/7.9) | [0.9, 1.8] |
| Age: (90, | 1 | 1.0 (1/1) | [0.03, 1] | 1.0 (1/1) | [1.0, 1.0] | nan (0/0) | N/A | 1.0 (1/1) | [1.0, 1.0] | 1.6 (1/0.6) | [1.6, 1.6] |

| | | | | | | | | | | | |
|---|---|---|---|---|---|---|---|---|---|---|---|
| 100] | | | | | | | | | | | |

**Supplemental Table 36: Stanford HM ACP in Inpatient Oncology: Class Balanced Reliability and Fairness Audit by Age.** Significant differences in prevalence, significantly lower performance, or significantly higher O/E are bolded.

| Group | Sample Size | Prevalence (Fraction) | Prevalence [95% CI] | Sensitivity (Fraction) | Sensitivity [95% CI] | Specificity (Fraction) | Specificity [95% CI] | Positive Predictive Value (Fraction) | Positive Predictive Value [95% CI] | O/E (Fraction) | O/E [95% CI] |
|---|---|---|---|---|---|---|---|---|---|---|---|
| Overall | 158 | 0.5 (79/158) | [0.42, 0.58] | 0.89 (70/79) | [0.82, 0.95] | 0.56 (44/79) | [0.45, 0.67] | 0.67 (70/105) | [0.58, 0.76] | 1.4 (79/57.0) | [1.2, 1.6] |
| Ethnicity: Not Hispanic or Latino, Race: White, Sex: Female | 27 | 0.33 (9/27) | [0.17, 0.54] | 1.0 (9/9) | [1.0, 1.0] | 0.33 (6/18) | [0.1, 0.55] | 0.43 (9/21) | [0.21, 0.64] | 0.9 (9/9.7) | [0.5, 1.6] |
| Ethnicity: Not Hispanic or Latino, Race: White, Sex: Male | 21 | 0.67 (14/21) | [0.43, 0.85] | 0.93 (13/14) | [0.86, 1.09] | 0.86 (6/7) | [0.71, 1.21] | 0.93 (13/14) | [0.86, 1.11] | 1.8 (14/7.8) | [1.3, 2.4] |
| Ethnicity: Not Hispanic or Latino, Race: Asian, Sex: Male | 21 | 0.76 (16/21) | [0.53, 0.92] | 0.94 (15/16) | [0.88, 1.08] | 0.8 (4/5) | [0.6, 1.27] | 0.94 (15/16) | [0.88, 1.08] | 1.9 (16/8.6) | [1.5, 2.4] |
| Ethnicity: Hispanic or Latino, Race: Other, Sex: Male | 19 | 0.63 (12/19) | [0.38, 0.84] | 0.83 (10/12) | [0.67, 1.07] | 0.71 (5/7) | [0.43, 1.1] | 0.83 (10/12) | [0.67, 1.07] | 1.7 (12/7.0) | [1.2, 2.4] |
| Ethnicity: Not Hispanic or Latino, Race: Asian, Sex: Female | 18 | 0.5 (9/18) | [0.26, 0.74] | 0.89 (8/9) | [0.78, 1.11] | 0.56 (5/9) | [0.21, 0.91] | 0.67 (8/12) | [0.41, 0.95] | 1.1 (9/8.4) | [0.7, 1.7] |
| Ethnicity: Hispanic or Latino, Race: Other, Sex: Female | 14 | 0.5 (7/14) | [0.23, 0.77] | 0.86 (6/7) | [0.71, 1.21] | 0.71 (5/7) | [0.43, 1.1] | 0.75 (6/8) | [0.5, 1.1] | 1.4 (7/5.0) | [0.8, 2.4] |
| Ethnicity: Not Hispanic or Latino, Race: Other, Sex: Male | 14 | 0.21 (3/14) | [0.05, 0.51] | 0.67 (2/3) | [0.33, 1.33] | 0.55 (6/11) | [0.26, 0.84] | 0.29 (2/7) | [-0.1, 0.57] | 0.9 (3/3.3 | [0.3, 2.5] |

| | | | | | | | | | | | |
|---|---|---|---|---|---|---|---|---|---|---|---|
| | | | | | | | | | | ) | |
| Ethnicity: Not Hispanic or Latino, Race: Black or African American, Sex: Female | 6 | 0.17 (1/6) | [0.0, 0.64] | **0.0 (0/1)** | **[0.0, 0.0]** | **0.0 (0/5)** | **[0.0, 0.0]** | **0.0 (0/5)** | **[0.0, 0.0]** | 0.7 (1/1.4) | [0.1, 4.3] |
| Ethnicity: Not Hispanic or Latino, Race: Other, Sex: Female | 5 | 1.0 (5/5) | [0.48, 1] | 1.0 (5/5) | [1.0, 1.0] | nan (0/0) | N/A | 1.0 (5/5) | [1.0, 1.0] | 2.0 (5/2.4) | [2.0, 2.0] |
| Ethnicity: Hispanic or Latino, Race: American Indian or Alaska Native, Sex: Male | 4 | 0.0 (0/4) | [0, 0.6] | nan (0/0) | N/A | 1.0 (4/4) | [1.0, 1.0] | nan (0/0) | N/A | 0.0 (0/0.4) | N/A |
| Ethnicity: Not Hispanic or Latino, Race: Black or African American, Sex: Male | 3 | 0.0 (0/3) | [0, 0.71] | nan (0/0) | N/A | 1.0 (3/3) | [1.0, 1.0] | nan (0/0) | N/A | 0.0 (0/0.5) | N/A |
| Ethnicity: Hispanic or Latino, Race: White, Sex: Male | 3 | 0.0 (0/3) | [0, 0.71] | nan (0/0) | N/A | **0.0 (0/3)** | **[0.0, 0.0]** | **0.0 (0/3)** | **[0.0, 0.0]** | 0.0 (0/0.8) | N/A |
| Ethnicity: Hispanic or Latino, Race: White, Sex: Female | 1 | 1.0 (1/1) | [0.03, 1] | 1.0 (1/1) | [1.0, 1.0] | nan (0/0) | N/A | 1.0 (1/1) | [1.0, 1.0] | 1.4 (1/0.7) | [1.4, 1.4] |
| Ethnicity: Not Hispanic or Latino, Race: American Indian or Alaska Native, Sex: Male | 1 | 1.0 (1/1) | [0.03, 1] | **0.0 (0/1)** | **[0.0, 0.0]** | nan (0/0) | N/A | nan (0/0) | N/A | **4.1 (1/0.2)** | **[4.1, 4.1]** |

**Supplemental Table 37: Stanford HM ACP in Inpatient Oncology: Class Balanced Reliability and Fairness Audit by Ethnicity/Race.** Significant differences in prevalence, significantly lower performance, or significantly higher O/E are bolded.

| Group | Sample Size | Prevalence (Fraction) | Prevalence [95% CI] | Sensitivity (Fraction) | Sensitivity [95% CI] | Specificity (Fraction) | Specificity [95% CI] | Positive Predictive Value (Fraction) | Positive Predictive Value [95% CI] | O/E (Fraction) | O/E [95% CI] |
|---|---|---|---|---|---|---|---|---|---|---|---|
| Overall | 158 | 0.5 (79/158) | [0.42, 0.58] | 0.89 (70/79) | [0.82, 0.95] | 0.56 (44/79) | [0.45, 0.67] | 0.67 (70/105) | [0.58, 0.76] | 1.4 (79/5 | [1.2, 1.6] |

| | | | | | | | | | | | |
|---|---|---|---|---|---|---|---|---|---|---|---|
| | | | | | | | | | | 7.0) | |
| Ethnicity: Not Hispanic or Latino, Race: White, Sex: Female | 27 | 0.33 (9/27) | [0.17, 0.54] | 1.0 (9/9) | [1.0, 1.0] | 0.33 (6/18) | [0.1, 0.55] | 0.43 (9/21) | [0.21, 0.64] | 0.9 (9/9.7) | [0.5, 1.6] |
| Ethnicity: Not Hispanic or Latino, Race: White, Sex: Male | 21 | 0.67 (14/21) | [0.43, 0.85] | 0.93 (13/14) | [0.86, 1.09] | 0.86 (6/7) | [0.71, 1.21] | 0.93 (13/14) | [0.86, 1.11] | 1.8 (14/7.8) | [1.3, 2.4] |
| Ethnicity: Not Hispanic or Latino, Race: Asian, Sex: Male | 21 | 0.76 (16/21) | [0.53, 0.92] | 0.94 (15/16) | [0.88, 1.08] | 0.8 (4/5) | [0.6, 1.27] | 0.94 (15/16) | [0.88, 1.08] | 1.9 (16/8.6) | [1.5, 2.4] |
| Ethnicity: Hispanic or Latino, Race: Other, Sex: Male | 19 | 0.63 (12/19) | [0.38, 0.84] | 0.83 (10/12) | [0.67, 1.07] | 0.71 (5/7) | [0.43, 1.1] | 0.83 (10/12) | [0.67, 1.07] | 1.7 (12/7.0) | [1.2, 2.4] |
| Ethnicity: Not Hispanic or Latino, Race: Asian, Sex: Female | 18 | 0.5 (9/18) | [0.26, 0.74] | 0.89 (8/9) | [0.78, 1.11] | 0.56 (5/9) | [0.21, 0.91] | 0.67 (8/12) | [0.41, 0.95] | 1.1 (9/8.4) | [0.7, 1.7] |
| Ethnicity: Hispanic or Latino, Race: Other, Sex: Female | 14 | 0.5 (7/14) | [0.23, 0.77] | 0.86 (6/7) | [0.71, 1.21] | 0.71 (5/7) | [0.43, 1.1] | 0.75 (6/8) | [0.5, 1.1] | 1.4 (7/5.0) | [0.8, 2.4] |
| Ethnicity: Not Hispanic or Latino, Race: Other, Sex: Male | 14 | 0.21 (3/14) | [0.05, 0.51] | 0.67 (2/3) | [0.33, 1.33] | 0.55 (6/11) | [0.26, 0.84] | **0.29 (2/7)** | **[-0.1, 0.57]** | 0.9 (3/3.3) | [0.3, 2.5] |
| Ethnicity: Not Hispanic or Latino, Race: Black or African American, Sex: Female | 6 | 0.17 (1/6) | [0.0, 0.64] | **0.0 (0/1)** | **[0.0, 0.0]** | **0.0 (0/5)** | **[0.0, 0.0]** | **0.0 (0/5)** | **[0.0, 0.0]** | 0.7 (1/1.4) | [0.1, 4.3] |
| Ethnicity: Not Hispanic or Latino, Race: Other, Sex: Female | 5 | 1.0 (5/5) | [0.48, 1] | 1.0 (5/5) | [1.0, 1.0] | nan (0/0) | N/A | 1.0 (5/5) | [1.0, 1.0] | **2.0 (5/2.4)** | **[2.0, 2.0]** |
| Ethnicity: Hispanic or Latino, Race: American Indian or Alaska Native, Sex: Male | 4 | 0.0 (0/4) | [0, 0.6] | nan (0/0) | N/A | 1.0 (4/4) | [1.0, 1.0] | nan (0/0) | N/A | 0.0 (0/0.4) | N/A |
| Ethnicity: Not Hispanic or Latino, Race: Black or African American, Sex: Male | 3 | 0.0 (0/3) | [0, 0.71] | nan (0/0) | N/A | 1.0 (3/3) | [1.0, 1.0] | nan (0/0) | N/A | 0.0 (0/0.5) | N/A |

| | | | | | | | | | | | |
|---|---|---|---|---|---|---|---|---|---|---|---|
| Ethnicity: Hispanic or Latino, Race: White, Sex: Male | 3 | 0.0 (0/3) | [0, 0.71] | nan (0/0) | N/A | **0.0 (0/3)** | **[0.0, 0.0]** | **0.0 (0/3)** | **[0.0, 0.0]** | 0.0 (0/0.8) | N/A |
| Ethnicity: Hispanic or Latino, Race: White, Sex: Female | 1 | 1.0 (1/1) | [0.03, 1] | 1.0 (1/1) | [1.0, 1.0] | nan (0/0) | N/A | 1.0 (1/1) | [1.0, 1.0] | 1.4 (1/0.7) | [1.4, 1.4] |
| Ethnicity: Not Hispanic or Latino, Race: American Indian or Alaska Native, Sex: Male | 1 | 1.0 (1/1) | [0.03, 1] | **0.0 (0/1)** | **[0.0, 0.0]** | nan (0/0) | N/A | nan (0/0) | N/A | **4.1 (1/0.2)** | **[4.1, 4.1]** |

**Supplemental Table 38: Stanford HM ACP in Inpatient Oncology: Class Balanced Reliability and Fairness Audit by Ethnicity/Race and Sex.** Significant differences in prevalence, significantly lower performance, or significantly higher O/E are bolded.

## *Epic EOL High Threshold in Hospital Medicine*

| Group | Sample Size | Prevalence (Fraction) | Prevalence [95% CI] | Sensitivity (Fraction) | Sensitivity [95% CI] | Specificity (Fraction) | Specificity [95% CI] | Positive Predictive Value (Fraction) | Positive Predictive Value [95% CI] | O/E (Fraction) | O/E [95% CI] |
|---|---|---|---|---|---|---|---|---|---|---|---|
| Overall | 344 | 0.5 (172/344) | [0.45, 0.55] | 0.21 (36/172) | [0.15, 0.27] | 0.95 (164/172) | [0.92, 0.99] | 0.82 (36/44) | [0.7, 0.93] | 2.6 (172/65.4) | [2.4, 2.9] |
| Sex: Female | 159 | 0.53 (85/159) | [0.45, 0.61] | 0.27 (23/85) | [0.18, 0.37] | 0.95 (70/74) | [0.9, 1.0] | 0.85 (23/27) | [0.73, 1.0] | 2.8 (85/30.8) | [2.4, 3.2] |
| Sex: Male | 185 | 0.47 (87/185) | [0.4, 0.54] | 0.15 (13/87) | [0.07, 0.22] | 0.96 (94/98) | [0.93, 1.0] | 0.76 (13/17) | [0.58, 0.98] | 2.5 (87/34.6) | [2.2, 2.9] |

**Supplemental Table 39: Epic EOL High Threshold in Hospital Medicine: Class Balanced Reliability and Fairness Audit by Sex.** Significant differences in prevalence, significantly lower performance, or significantly higher O/E are bolded.

| Group | Sample Size | Prevalence (Fraction) | Prevalence [95% CI] | Sensitivity (Fraction) | Sensitivity [95% CI] | Specificity (Fraction) | Specificity [95% CI] | Positive Predictive Value (Fraction) | Positive Predictive Value [95% CI] | O/E (Fraction) | O/E [95% CI] |
|---|---|---|---|---|---|---|---|---|---|---|---|
| Overall | 344 | 0.5 (172/344) | [0.45, 0.55] | 0.21 (36/172) | [0.15, 0.27] | 0.95 (164/172) | [0.92, 0.99] | 0.82 (36/44) | [0.7, 0.93] | 2.6 (172/65.4) | [2.4, 2.9] |
| Age: (10, 20] | 3 | 0.33 (1/3) | [0.01, 0.91] | **0.0 (0/1)** | **[0.0, 0.0]** | 1.0 (2/2) | [1.0, 1.0] | nan (0/0) | N/A | inf (1/0.0) | [inf, inf] |
| Age: (20, 30] | 27 | **0.22 (6/27)** | **[0.09, 0.42]** | **0.0 (0/6)** | **[0.0, 0.0]** | 1.0 (21/21) | [1.0, 1.0] | nan (0/0) | N/A | **7.5 (6/0.8)** | **[3.7, 15.2]** |
| Age: (30, 40] | 40 | **0.15 (6/40)** | **[0.06, 0.3]** | **0.0 (0/6)** | **[0.0, 0.0]** | 1.0 (34/34) | [1.0, 1.0] | nan (0/0) | N/A | 4.7 (6/1.3) | [2.2, 9.7] |
| Age: (40, 50] | 14 | 0.57 (8/14) | [0.29, 0.82] | 0.12 (1/8) | [-0.12, 0.25] | 1.0 (6/6) | [1.0, 1.0] | 1.0 (1/1) | [1.0, 1.0] | 4.4 (8/1.8) | [2.8, 6.9] |
| Age: (50, 60] | 45 | 0.36 (16/45) | [0.22, 0.51] | **0.0 (0/16)** | **[0.0, 0.0]** | 1.0 (29/29) | [1.0, 1.0] | nan (0/0) | N/A | 4.1 (16/3.9) | [2.8, 6.1] |
| Age: (60, 70] | 75 | 0.47 (35/75) | [0.35, 0.59] | 0.23 (8/35) | [0.08, 0.35] | 0.92 (37/40) | [0.85, 1.02] | 0.73 (8/11) | [0.45, 1.0] | 2.1 (35/16.5) | [1.7, 2.7] |
| Age: (70, 80] | 72 | 0.57 (41/72) | [0.45, 0.69] | 0.32 (13/41) | [0.17, 0.44] | 0.9 (28/31) | [0.81, 1.03] | 0.81 (13/16) | [0.62, 1.04] | 2.4 (41/17.3) | [1.9, 2.9] |
| Age: (80, 90] | 42 | 0.81 (34/42) | [0.66, 0.91] | 0.18 (6/34) | [0.04, 0.3] | 0.88 (7/8) | [0.75, 1.15] | 0.86 (6/7) | [0.71, 1.21] | 2.8 (34/12.4) | [2.4, 3.2] |

| | | | | | | | | | | | |
|---|---|---|---|---|---|---|---|---|---|---|---|
| Age: (90, 100] | 26 | 0.96 (25/26) | [0.8, 1.0] | 0.32 (8/25) | [0.14, 0.49] | **0.0 (0/1)** | **[0.0, 0.0]** | 0.89 (8/9) | [0.78, 1.14] | 2.2 (25/11.5) | [2.0, 2.4] |

**Supplemental Table 40: Epic EOL High Threshold in Hospital Medicine: Class Balanced Reliability and Fairness Audit by Age.** Significant differences in prevalence, significantly lower performance, or significantly higher O/E are bolded.

| Group | Sample Size | Prevalence (Fraction) | Prevalence [95% CI] | Sensitivity (Fraction) | Sensitivity [95% CI] | Specificity (Fraction) | Specificity [95% CI] | Positive Predictive Value (Fraction) | Positive Predictive Value [95% CI] | O/E (Fraction) | O/E [95% CI] |
|---|---|---|---|---|---|---|---|---|---|---|---|
| Overall | 344 | 0.5 (172/344) | [0.45, 0.55] | 0.21 (36/172) | [0.15, 0.27] | 0.95 (164/172) | [0.92, 0.99] | 0.82 (36/44) | [0.7, 0.93] | 2.6 (172/65.4) | [2.4, 2.9] |
| Ethnicity: Not Hispanic or Latino, Race: White | 159 | 0.49 (78/159) | [0.41, 0.57] | **0.09 (7/78)** | **[0.02, 0.15]** | 0.95 (77/81) | [0.91, 1.0] | 0.64 (7/11) | [0.37, 0.97] | **3.4 (78/22.8)** | **[2.9, 4.0]** |
| Ethnicity: Not Hispanic or Latino, Race: Asian | 50 | **0.76 (38/50)** | **[0.62, 0.87]** | 0.34 (13/38) | [0.18, 0.47] | 1.0 (12/12) | [1.0, 1.0] | 1.0 (13/13) | [1.0, 1.0] | 2.1 (38/17.8) | [1.8, 2.5] |
| Ethnicity: Hispanic or Latino, Race: Other | 47 | **0.23 (11/47)** | **[0.12, 0.38]** | 0.09 (1/11) | [-0.13, 0.18] | 0.94 (34/36) | [0.89, 1.02] | **0.33 (1/3)** | **[-0.33, 0.67]** | 2.6 (11/4.3) | [1.5, 4.3] |
| Ethnicity: Not Hispanic or Latino, Race: Black or African American | 41 | 0.61 (25/41) | [0.45, 0.76] | 0.52 (13/25) | [0.33, 0.71] | 0.88 (14/16) | [0.75, 1.06] | 0.87 (13/15) | [0.73, 1.07] | 1.6 (25/16.1) | [1.2, 2.0] |
| Ethnicity: Not Hispanic or Latino, Race: Other | 17 | 0.53 (9/17) | [0.28, 0.77] | 0.22 (2/9) | [-0.06, 0.44] | 1.0 (8/8) | [1.0, 1.0] | 1.0 (2/2) | [1.0, 1.0] | 4.4 (9/2.0) | [2.8, 6.9] |
| Ethnicity: Hispanic or Latino, Race: White | 14 | 0.29 (4/14) | [0.08, 0.58] | **0.0 (0/4)** | **[0.0, 0.0]** | 1.0 (10/10) | [1.0, 1.0] | nan (0/0) | N/A | 5.5 (4/0.7) | [2.4, 12.5] |

| | | | | | | | | | | | |
|---|---|---|---|---|---|---|---|---|---|---|---|
| Ethnicity: Not Hispanic or Latino, Race: Native Hawaiian or Other Pacific Islander | 10 | 0.4 (4/10) | [0.12, 0.74] | **0.0 (0/4)** | **[0.0, 0.0]** | 1.0 (6/6) | [1.0, 1.0] | nan (0/0) | N/A | 3.0 (4/1.3) | [1.4, 6.4] |
| Ethnicity: Not Hispanic or Latino, Race: American Indian or Alaska Native | 1 | 0.0 (0/1) | [0, 0.98] | nan (0/0) | N/A | 1.0 (1/1) | [1.0, 1.0] | nan (0/0) | N/A | 0.0 (0/0.2) | N/A |

**Supplemental Table 41: Epic EOL High Threshold in Hospital Medicine: Class Balanced Reliability and Fairness Audit by Ethnicity/Race.** Significant differences in prevalence, significantly lower performance, or significantly higher O/E are bolded.

| Group | Sample Size | Prevalence (Fraction) | Prevalence [95% CI] | Sensitivity (Fraction) | Sensitivity [95% CI] | Specificity (Fraction) | Specificity [95% CI] | Positive Predictive Value (Fraction) | Positive Predictive Value [95% CI] | O/E (Fraction) | O/E [95% CI] |
|---|---|---|---|---|---|---|---|---|---|---|---|
| Overall | 344 | 0.5 (172/344) | [0.45, 0.55] | 0.21 (36/172) | [0.15, 0.27] | 0.95 (164/172) | [0.92, 0.99] | 0.82 (36/44) | [0.7, 0.93] | 2.6 (172/65.4) | [2.4, 2.9] |
| Ethnicity: Not Hispanic or Latino, Race: White, Sex: Male | 87 | 0.43 (37/87) | [0.32, 0.54] | 0.08 (3/37) | [-0.01, 0.16] | 0.94 (47/50) | [0.88, 1.01] | 0.5 (3/6) | [0.0, 1.0] | 2.8 (37/13.0) | [2.2, 3.6] |
| Ethnicity: Not Hispanic or Latino, Race: White, Sex: Female | 72 | 0.57 (41/72) | [0.45, 0.69] | 0.1 (4/41) | [0.0, 0.17] | 0.97 (30/31) | [0.94, 1.05] | 0.8 (4/5) | [0.6, 1.31] | **4.2 (41/9.8)** | **[3.4, 5.1]** |
| Ethnicity: Not Hispanic or Latino, Race: Asian, Sex: Male | 29 | **0.79 (23/29)** | **[0.6, 0.92]** | 0.13 (3/23) | [-0.0, 0.26] | 1.0 (6/6) | [1.0, 1.0] | 1.0 (3/3) | [1.0, 1.0] | 2.3 (23/9.9) | [1.9, 2.8] |
| Ethnicity: Hispanic or Latino, Race: Other, Sex: Female | 24 | 0.29 (7/24) | [0.13, 0.51] | 0.14 (1/7) | [-0.21, 0.29] | 0.88 (15/17) | [0.76, 1.06] | **0.33 (1/3)** | **[-0.33, 0.67]** | 2.0 (7/3.6) | [1.0, 3.6] |
| Ethnicity: Hispanic or Latino, Race: Other, Sex: Male | 23 | **0.17 (4/23)** | **[0.05, 0.39]** | **0.0 (0/4)** | **[0.0, 0.0]** | 1.0 (19/19) | [1.0, 1.0] | nan (0/0) | N/A | 5.5 (4/0.7) | [2.2, 13.4] |
| Ethnicity: Not Hispanic or Latino, Race: Black or African American, Sex: Male | 23 | 0.52 (12/23) | [0.31, 0.73] | 0.58 (7/12) | [0.31, 0.87] | 0.91 (10/11) | [0.82, 1.1] | 0.88 (7/8) | [0.75, 1.12] | 1.3 (12/8 | [0.9, 2.0] |

| | | | | | | | | | | | |
|---|---|---|---|---|---|---|---|---|---|---|---|
| | | | | | | | | | | .9) | |
| Ethnicity: Not Hispanic or Latino, Race: Asian, Sex: Female | 21 | 0.71 (15/21) | [0.48, 0.89] | 0.67 (10/15) | [0.46, 0.9] | 1.0 (6/6) | [1.0, 1.0] | 1.0 (10/10) | [1.0, 1.0] | 1.9 (15/7.8) | [1.5, 2.5] |
| Ethnicity: Not Hispanic or Latino, Race: Black or African American, Sex: Female | 18 | 0.72 (13/18) | [0.47, 0.9] | 0.46 (6/13) | [0.17, 0.72] | 0.8 (4/5) | [0.6, 1.27] | 0.86 (6/7) | [0.71, 1.16] | 1.8 (13/7.2) | [1.4, 2.4] |
| Ethnicity: Not Hispanic or Latino, Race: Other, Sex: Male | 10 | 0.6 (6/10) | [0.26, 0.88] | **0.0 (0/6)** | **[0.0, 0.0]** | 1.0 (4/4) | [1.0, 1.0] | nan (0/0) | N/A | **15.8 (6/0.4)** | **[9.5, 26.2]** |
| Ethnicity: Hispanic or Latino, Race: White, Sex: Male | 8 | 0.25 (2/8) | [0.03, 0.65] | **0.0 (0/2)** | **[0.0, 0.0]** | 1.0 (6/6) | [1.0, 1.0] | nan (0/0) | N/A | **13.3 (2/0.2)** | **[4.0, 44.3]** |
| Ethnicity: Not Hispanic or Latino, Race: Other, Sex: Female | 7 | 0.43 (3/7) | [0.1, 0.82] | 0.67 (2/3) | [0.33, 1.33] | 1.0 (4/4) | [1.0, 1.0] | 1.0 (2/2) | [1.0, 1.0] | 1.8 (3/1.7) | [0.8, 4.3] |
| Ethnicity: Hispanic or Latino, Race: White, Sex: Female | 6 | 0.33 (2/6) | [0.04, 0.78] | **0.0 (0/2)** | **[0.0, 0.0]** | 1.0 (4/4) | [1.0, 1.0] | nan (0/0) | N/A | 3.4 (2/0.6) | [1.1, 10.7] |
| Ethnicity: Not Hispanic or Latino, Race: Native Hawaiian or Other Pacific Islander, Sex: Female | 6 | 0.17 (1/6) | [0.0, 0.64] | **0.0 (0/1)** | **[0.0, 0.0]** | 1.0 (5/5) | [1.0, 1.0] | nan (0/0) | N/A | 7.7 (1/0.1) | [1.3, 46.0] |
| Ethnicity: Not Hispanic or Latino, Race: Native Hawaiian or Other Pacific Islander, Sex: Male | 4 | 0.75 (3/4) | [0.19, 0.99] | **0.0 (0/3)** | **[0.0, 0.0]** | 1.0 (1/1) | [1.0, 1.0] | nan (0/0) | N/A | 2.5 (3/1.2) | [1.4, 4.4] |
| Ethnicity: Not Hispanic or Latino, Race: American Indian or Alaska Native, Sex: Male | 1 | 0.0 (0/1) | [0, 0.98] | nan (0/0) | N/A | 1.0 (1/1) | [1.0, 1.0] | nan (0/0) | N/A | 0.0 (0/0.2) | N/A |

**Supplemental Table 42: Epic EOL High Threshold in Hospital Medicine: Class Balanced Reliability and Fairness Audit by Ethnicity/Race and Sex.** Significant differences in prevalence, significantly lower performance, or significantly higher O/E are bolded.

*Stanford HM ACP in Hospital Medicine*

| Group | Sample Size | Prevalence (Fraction) | Prevalence [95% CI] | Sensitivity (Fraction) | Sensitivity [95% CI] | Specificity (Fraction) | Specificity [95% CI] | Positive Predictive Value (Fraction) | Positive Predictive Value [95% CI] | O/E (Fraction) | O/E [95% CI] |
|---|---|---|---|---|---|---|---|---|---|---|---|
| Overall | 252 | 0.5 (126/252) | [0.44, 0.56] | 0.71 (89/126) | [0.63, 0.79] | 0.87 (109/126) | [0.81, 0.93] | 0.84 (89/106) | [0.77, 0.91] | 1.6 (126/77.0) | [1.4, 1.9] |
| Sex: Female | 130 | 0.52 (67/130) | [0.43, 0.6] | 0.7 (47/67) | [0.6, 0.82] | 0.83 (52/63) | [0.73, 0.92] | 0.81 (47/58) | [0.71, 0.92] | 1.6 (67/41.8) | [1.4, 1.9] |
| Sex: Male | 122 | 0.48 (59/122) | [0.39, 0.58] | 0.71 (42/59) | [0.59, 0.83] | 0.9 (57/63) | [0.84, 0.98] | 0.88 (42/48) | [0.79, 0.97] | 1.7 (59/35.2) | [1.4, 2.0] |

**Supplemental Table 43: Stanford HM ACP in Hospital Medicine: Class Balanced Reliability and Fairness Audit by Sex.** Significant differences in prevalence, significantly lower performance, or significantly higher O/E are bolded.

| Group | Sample Size | Prevalence (Fraction) | Prevalence [95% CI] | Sensitivity (Fraction) | Sensitivity [95% CI] | Specificity (Fraction) | Specificity [95% CI] | Positive Predictive Value (Fraction) | Positive Predictive Value [95% CI] | O/E (Fraction) | O/E [95% CI] |
|---|---|---|---|---|---|---|---|---|---|---|---|
| Overall | 252 | 0.5 (126/252) | [0.44, 0.56] | 0.71 (89/126) | [0.63, 0.79] | 0.87 (109/126) | [0.81, 0.93] | 0.84 (89/106) | [0.77, 0.91] | 1.6 (126/77.0) | [1.4, 1.9] |
| Age: (10, 20] | 3 | 0.33 (1/3) | [0.01, 0.91] | **0.0 (0/1)** | **[0.0, 0.0]** | 1.0 (2/2) | [1.0, 1.0] | nan (0/0) | N/A | 2.4 (1/0.4) | [0.5, 12.1] |
| Age: (20, 30] | 16 | 0.31 (5/16) | [0.11, 0.59] | 0.8 (4/5) | [0.6, 1.27] | 1.0 (11/11) | [1.0, 1.0] | 1.0 (4/4) | [1.0, 1.0] | 1.2 (5/4.2) | [0.6, 2.5] |

| | | | | | | | | | | | |
|---|---|---|---|---|---|---|---|---|---|---|---|
| Age: (30, 40] | 29 | **0.14 (4/29)** | **[0.04, 0.32]** | **0.0 (0/4)** | **[0.0, 0.0]** | 1.0 (25/25) | [1.0, 1.0] | nan (0/0) | N/A | 1.0 (4/4.0) | [0.4, 2.5] |
| Age: (40, 50] | 16 | 0.44 (7/16) | [0.2, 0.7] | 0.43 (3/7) | [0.06, 0.86] | 1.0 (9/9) | [1.0, 1.0] | 1.0 (3/3) | [1.0, 1.0] | 1.8 (7/4.0) | [1.0, 3.1] |
| Age: (50, 60] | 25 | 0.24 (6/25) | [0.09, 0.45] | **0.17 (1/6)** | **[-0.17, 0.33]** | 1.0 (19/19) | [1.0, 1.0] | 1.0 (1/1) | [1.0, 1.0] | 1.5 (6/4.1) | [0.7, 2.9] |
| Age: (60, 70] | 55 | 0.42 (23/55) | [0.29, 0.56] | 0.83 (19/23) | [0.69, 0.99] | 0.84 (27/32) | [0.72, 1.0] | 0.79 (19/24) | [0.64, 0.97] | 1.4 (23/16.7) | [1.0, 1.9] |
| Age: (70, 80] | 55 | 0.62 (34/55) | [0.48, 0.75] | 0.82 (28/34) | [0.71, 0.95] | **0.57 (12/21)** | **[0.38, 0.79]** | 0.76 (28/37) | [0.63, 0.89] | 1.4 (34/24.1) | [1.1, 1.7] |
| Age: (80, 90] | 36 | **0.83 (30/36)** | **[0.67, 0.94]** | 0.73 (22/30) | [0.58, 0.9] | 0.5 (3/6) | [0.0, 1.0] | 0.88 (22/25) | [0.76, 1.02] | **2.2 (30/13.7)** | **[1.9, 2.5]** |
| Age: (90, 100] | 17 | **0.94 (16/17)** | **[0.71, 1.0]** | 0.75 (12/16) | [0.56, 0.97] | 1.0 (1/1) | [1.0, 1.0] | 1.0 (12/12) | [1.0, 1.0] | **2.7 (16/6.0)** | **[2.4, 3.0]** |

**Supplemental Table 44: Stanford HM ACP in Hospital Medicine: Class Balanced Reliability and Fairness Audit by Age.** Significant differences in prevalence, significantly lower performance, or significantly higher O/E are bolded.

| Group | Sample Size | Prevalence (Fraction) | Prevalence [95% CI] | Sensitivity (Fraction) | Sensitivity [95% CI] | Specificity (Fraction) | Specificity [95% CI] | Positive Predictive Value (Fraction) | Positive Predictive Value [95% CI] | O/E (Fraction) | O/E [95% CI] |
|---|---|---|---|---|---|---|---|---|---|---|---|
| Overall | 252 | 0.5 (126/252) | [0.44, 0.56] | 0.71 (89/126) | [0.63, 0.79] | 0.87 (109/126) | [0.81, 0.93] | 0.84 (89/106) | [0.77, 0.91] | 1.6 (126/77.0) | [1.4, 1.9] |

| | | | | | | | | | | | |
|---|---|---|---|---|---|---|---|---|---|---|---|
| Ethnicity: Not Hispanic or Latino, Race: White | 91 | 0.51 (46/91) | [0.4, 0.61] | 0.65 (30/46) | [0.52, 0.79] | 0.91 (41/45) | [0.84, 1.0] | 0.88 (30/34) | [0.79, 1.0] | 2.0 (46/23.5) | [1.6, 2.4] |
| Ethnicity: Not Hispanic or Latino, Race: Asian | 41 | **0.73 (30/41)** | **[0.57, 0.86]** | 0.73 (22/30) | [0.59, 0.9] | 0.91 (10/11) | [0.82, 1.13] | 0.96 (22/23) | [0.91, 1.06] | 1.6 (30/18.5) | [1.3, 1.9] |
| Ethnicity: Hispanic or Latino, Race: Other | 39 | **0.18 (7/39)** | **[0.08, 0.34]** | 0.43 (3/7) | [0.02, 0.86] | 0.84 (27/32) | [0.72, 0.98] | **0.38 (3/8)** | **[0.0, 0.75]** | 1.0 (7/7.3) | [0.5, 1.9] |
| Ethnicity: Not Hispanic or Latino, Race: Black or African American | 25 | 0.52 (13/25) | [0.31, 0.72] | 0.69 (9/13) | [0.46, 0.94] | 0.83 (10/12) | [0.67, 1.07] | 0.82 (9/11) | [0.64, 1.08] | 1.6 (13/8.1) | [1.1, 2.3] |
| Ethnicity: Hispanic or Latino, Race: White | 14 | 0.43 (6/14) | [0.18, 0.71] | 0.83 (5/6) | [0.67, 1.17] | 0.88 (7/8) | [0.75, 1.15] | 0.83 (5/6) | [0.67, 1.17] | 1.7 (6/3.5) | [0.9, 3.1] |
| Ethnicity: Not Hispanic or Latino, Race: Native Hawaiian or Other Pacific Islander | 12 | 0.5 (6/12) | [0.21, 0.79] | 0.83 (5/6) | [0.67, 1.17] | 1.0 (6/6) | [1.0, 1.0] | 1.0 (5/5) | [1.0, 1.0] | 2.1 (6/2.8) | [1.2, 3.8] |
| Ethnicity: Not Hispanic or Latino, Race: Other | 10 | 0.5 (5/10) | [0.19, 0.81] | 0.4 (2/5) | [-0.2, 0.8] | 1.0 (5/5) | [1.0, 1.0] | 1.0 (2/2) | [1.0, 1.0] | 1.4 (5/3.5) | [0.8, 2.6] |

**Supplemental Table 45: Stanford HM ACP in Hospital Medicine: Class Balanced Reliability and Fairness Audit by Ethnicity/Race.** Significant differences in prevalence, significantly lower performance, or significantly higher O/E are bolded.

| Group | Sample Size | Prevalence (Fraction) | Prevalence [95% CI] | Sensitivity (Fraction) | Sensitivity [95% CI] | Specificity (Fraction) | Specificity [95% CI] | Positive Predictive Value (Fraction) | Positive Predictive Value [95% CI] | O/E (Fraction) | O/E [95% CI] |
|---|---|---|---|---|---|---|---|---|---|---|---|
| Overall | 252 | 0.5 (126/252) | [0.44, 0.56] | 0.71 (89/126) | [0.63, 0.79] | 0.87 (109/126) | [0.81, 0.93] | 0.84 (89/106) | [0.77, 0.91] | 1.6 (126/77.0) | [1.4, 1.9] |

| | | | | | | | | | | | |
|---|---|---|---|---|---|---|---|---|---|---|---|
| Ethnicity: Not Hispanic or Latino, Race: White, Sex: Female | 51 | 0.61 (31/51) | [0.46, 0.74] | 0.61 (19/31) | [0.44, 0.79] | 0.85 (17/20) | [0.7, 1.02] | 0.86 (19/22) | [0.73, 1.04] | 2.1 (31/15.0) | [1.7, 2.6] |
| Ethnicity: Not Hispanic or Latino, Race: White, Sex: Male | 40 | 0.38 (15/40) | [0.23, 0.54] | 0.73 (11/15) | [0.53, 0.97] | 0.96 (24/25) | [0.92, 1.05] | 0.92 (11/12) | [0.83, 1.12] | 1.8 (15/8.5) | [1.2, 2.6] |
| Ethnicity: Not Hispanic or Latino, Race: Asian, Sex: Male | 24 | **0.83 (20/24)** | **[0.63, 0.95]** | 0.7 (14/20) | [0.5, 0.9] | 0.75 (3/4) | [0.5, 1.5] | 0.93 (14/15) | [0.87, 1.08] | 1.7 (20/11.9) | [1.4, 2.0] |
| Ethnicity: Hispanic or Latino, Race: Other, Sex: Female | 23 | **0.22 (5/23)** | **[0.07, 0.44]** | 0.6 (3/5) | [0.2, 1.2] | 0.83 (15/18) | [0.67, 1.02] | 0.5 (3/6) | [0.0, 1.0] | 1.0 (5/5.1) | [0.5, 2.1] |
| Ethnicity: Not Hispanic or Latino, Race: Asian, Sex: Female | 17 | 0.59 (10/17) | [0.33, 0.82] | 0.8 (8/10) | [0.6, 1.1] | 1.0 (7/7) | [1.0, 1.0] | 1.0 (8/8) | [1.0, 1.0] | 1.5 (10/6.7) | [1.0, 2.2] |
| Ethnicity: Hispanic or Latino, Race: Other, Sex: Male | 16 | **0.12 (2/16)** | **[0.02, 0.38]** | **0.0 (0/2)** | **[0.0, 0.0]** | 0.86 (12/14) | [0.71, 1.05] | **0.0 (0/2)** | **[0.0, 0.0]** | 0.9 (2/2.2) | [0.3, 3.4] |
| Ethnicity: Not Hispanic or Latino, Race: Black or African American, Sex: Female | 16 | 0.75 (12/16) | [0.48, 0.93] | 0.75 (9/12) | [0.5, 1.0] | 0.75 (3/4) | [0.5, 1.3] | 0.9 (9/10) | [0.8, 1.13] | 1.8 (12/6.8) | [1.3, 2.3] |
| Ethnicity: Not Hispanic or Latino, Race: Black or African American, Sex: Male | 9 | 0.11 (1/9) | [0.0, 0.48] | **0.0 (0/1)** | **[0.0, 0.0]** | 0.88 (7/8) | [0.75, 1.12] | **0.0 (0/1)** | **[0.0, 0.0]** | 0.8 (1/1.3) | [0.1, 5.0] |
| Ethnicity: Hispanic or Latino, Race: White, Sex: Male | 8 | 0.38 (3/8) | [0.09, 0.76] | 0.67 (2/3) | [0.33, 1.33] | 1.0 (5/5) | [1.0, 1.0] | 1.0 (2/2) | [1.0, 1.0] | 2.2 (3/1.4) | [0.9, 5.4] |
| Ethnicity: Hispanic or Latino, Race: White, Sex: Female | 6 | 0.5 (3/6) | [0.12, 0.88] | 1.0 (3/3) | [1.0, 1.0] | 0.67 (2/3) | [0.33, 1.33] | 0.75 (3/4) | [0.5, 1.25] | 1.4 (3/2.2) | [0.6, 3.1] |
| Ethnicity: Not Hispanic or Latino, Race: Native Hawaiian or Other Pacific Islander, Sex: Female | 6 | 0.17 (1/6) | [0.0, 0.64] | **0.0 (0/1)** | **[0.0, 0.0]** | 1.0 (5/5) | [1.0, 1.0] | nan (0/0) | N/A | 0.8 (1/1.2) | [0.1, 5.0] |
| Ethnicity: Not Hispanic or Latino, Race: Native Hawaiian or Other Pacific Islander, Sex: Male | 6 | 0.83 (5/6) | [0.36, 1.0] | 1.0 (5/5) | [1.0, 1.0] | 1.0 (1/1) | [1.0, 1.0] | 1.0 (5/5) | [1.0, 1.0] | **3.1 (5/1.6)** | **[2.2, 4.5]** |

| | | | | | | | | | | | |
|---|---|---|---|---|---|---|---|---|---|---|---|
| Ethnicity: Not Hispanic or Latino, Race: Other, Sex: Male | 6 | 0.5 (3/6) | [0.12, 0.88] | **0.0 (0/3)** | **[0.0, 0.0]** | 1.0 (3/3) | [1.0, 1.0] | nan (0/0) | N/A | 2.3 (3/1.3) | [1.0, 5.0] |
| Ethnicity: Not Hispanic or Latino, Race: Other, Sex: Female | 4 | 0.5 (2/4) | [0.07, 0.93] | 1.0 (2/2) | [1.0, 1.0] | 1.0 (2/2) | [1.0, 1.0] | 1.0 (2/2) | [1.0, 1.0] | 0.9 (2/2.2) | [0.3, 2.4] |

**Supplemental Table 46: Stanford HM ACP in Hospital Medicine: Class Balanced Reliability and Fairness Audit by Ethnicity/Race and Sex.** Significant differences in prevalence, significantly lower performance, or significantly higher O/E are bolded.
